# Synthesis and Biological Activity of New 7-Amino-oxazolo[5,4-*d*]Pyrimidine Derivatives

**DOI:** 10.3390/molecules25153558

**Published:** 2020-08-04

**Authors:** Aleksandra Sochacka-Ćwikła, Andrzej Regiec, Michał Zimecki, Jolanta Artym, Ewa Zaczyńska, Maja Kocięba, Iwona Kochanowska, Iwona Bryndal, Anna Pyra, Marcin Mączyński

**Affiliations:** 1 Department of Organic Chemistry, Faculty of Pharmacy, Wroclaw Medical University, 211A Borowska Street, 50-556 Wroclaw, Poland; aleksandra.sochacka-cwikla@umed.wroc.pl (A.S.-Ć.); marcin.maczynski@umed.wroc.pl (M.M.); 2 Department of Experimental Therapy, Institute of Immunology and Experimental Therapy, Polish Academy of Sciences, 12 Rudolf Weigl Street, 53-114 Wroclaw, Poland; michal.zimecki@hirszfeld.pl (M.Z.); jolanta.artym@hirszfeld.pl (J.A.); ewa.zaczynska@hirszfeld.pl (E.Z.); maja.kocieba@hirszfeld.pl (M.K.); iwona.kochanowska@hirszfeld.pl (I.K.); 3 Department of Drug Technology, Faculty of Pharmacy, Wroclaw Medical University, 211A Borowska Street, 50-556 Wroclaw, Poland; iwona.bryndal@umed.wroc.pl; 4 Faculty of Chemistry, University of Wroclaw, 14 Joliot-Curie, 50-383 Wroclaw, Poland; anna.pyra@uwr.edu.pl

**Keywords:** oxazolo[5,4-*d*]pyrimidines, acetamidines, imidates, synthesis, spectral analysis, X-ray crystallography, immunosuppression, proliferation, apoptosis, antiviral activity

## Abstract

The synthesis of a series of novel 7-aminooxazolo[5,4-*d*]pyrimidines **5**, transformations during their synthesis and their physicochemical characteristics have been described. Complete detailed spectral analysis of the intermediates **2**–**4**, the *N′*-cyanooxazolylacetamidine by-products **7** and final compounds **5** has been carried out using MS, IR, 1D and 2D NMR spectroscopy. Theoretical research was carried out to explain the privileged formation of 7-aminooxazolo[5,4-*d*]pyrimidines in relation to the possibility of their isomer formation and the related thermodynamic aspects. Additionally, the single-crystal X-ray diffraction analysis for **5h** was reported. Ten 7-aminooxazolo[5,4-*d*]pyrimidines **5** (**SCM1**–**10**) were biologically tested in vitro to preliminarily evaluate their immunological, antiviral and anticancer activity. Compounds **SCM5** and **SCM9** showed the best immunoregulatory profile. The compounds displayed low-toxicity and strongly inhibited phytohemagglutinin A-induced proliferation of human peripheral blood lymphocytes and lipopolysaccharide-induced proliferation of mouse splenocytes. Compound **SCM9** caused also a moderate suppression of tumor necrosis factor α (TNF-α) production in a human whole blood culture. Of note, the compounds also inhibited the growth of selected tumor cell lines and inhibited replication of human herpes virus type-1 (HHV-1) virus in A-549 cell line. Molecular investigations showed that the compounds exerted differential changes in expression of signaling proteins in Jurkat and WEHI-231 cell lines. The activity of **SCM5** is likely associated with elicitation of cell signaling pathways leading to cell apoptosis. The compounds may be of interest in terms of therapeutic utility as inhibitors of autoimmune disorders, virus replication and antitumor agents.

## 1. Introduction

**Figure 1:**
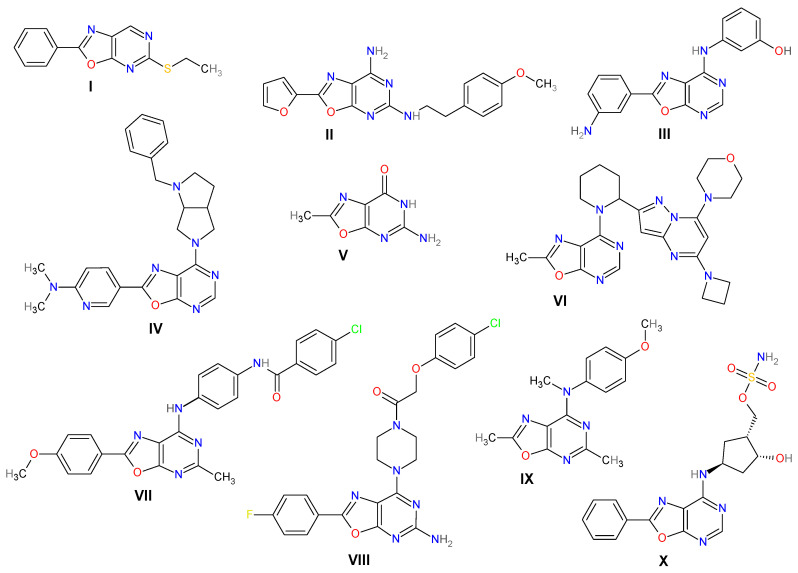
Selected oxazolo[5,4-*d*]pyrimidines showing biological activity.

The oxazolo[5,4-*d*]pyrimidine system was synthesized for the first time by Clapp as a derivative, i.e., 5-(ethylsulfanyl)-2-phenyl[1,3]oxazolo[5,4-*d*]pyrimidine (Structure I in Figure 1), which was described and revealed in 1905 by Johnson [1].

Since that time, many derivatives of oxazolo[5,4-*d*]pyrimidine system have been synthesized and extensively researched for their potential biological activity. Some oxazolo[5,4-*d*]pyrimidines are well known as adenosine receptor antagonists (Structure II in Figure 1) [2], inhibitors of receptor tyrosine kinase (Structure III in Figure 1) [3], inhibitors of adenosine kinase (Structure IV in Figure 1) [4], effective inhibitors of ricin [5] and Shiga toxin inhibitors (Structure V in Figure 1) [6]. The antiviral (Structure VI in Figure 1) [7], anti-angiogenic (Structure VII in Figure 1) [8] and immunosuppressive (Structure VIII in Figure 1) [9] activity of some of them has been also revealed. Besides, some oxazolo[5,4-*d*]pyrimidines can also activate the caspase cascade (Structure IX in Figure 1) [10] and inhibit ubiquitin-activating enzymes (E1 enzymes), particularly NEDD8-activating enzyme (NAE) (Structure X in Figure 1) [11], which may result in their antineoplastic activity.

In our previous search for novel potent immunomodulatory, anti-inflammatory and anticancer agents, many of five-membered heterocycles with two heteroatoms, such as 5-amino-3-methyl-4-isoxazolecarboxylic acid [12], 5-amino-3-methyl-4-isothiazolecarboxylic acid [13,14,15] and 4-amino-1-methyl-5-imidazolecarboxylic acid derivatives [16] were designed and synthesized. The described results revealed that the isoxazole moiety was particularly responsible for the immunological activity of these compounds. These derivatives exhibited a variety of activities, such as immunosuppressive [17,18,19,20,21], immunostimulatory [22,23] and anti-inflammatory [24,25].

The aim of this work was the synthesis of 7-aminooxazolo[5,4-*d*]pyrimidines series with 5-amino-3-methyl-isoxazol-4-yl substituent at position 2 of their structure to find novel derivatives exhibiting potential immunosuppressive activity. It is worth noting that oxazolo[5,4-*d*]pyrimidine system is structurally similar to purine analogs of naturally occurring nucleic acid bases. Such a structure may, in theory, condition antitumor and/or antiviral activity, so for this purpose, several in vitro immunological and antitumor assays have been designed and carried out. Finally, on the basis of the obtained results, an attempt to establish a plausible mechanism of the biological action of these compounds has been made.

## 2. Results and Discussion

### 2.1. Chemistry

#### 2.1.1. Synthesis and Some Physicochemical Properties of New 7-Aminooxazolo[5,4-*d*]Pyrimidines **5**

**Scheme 1:**
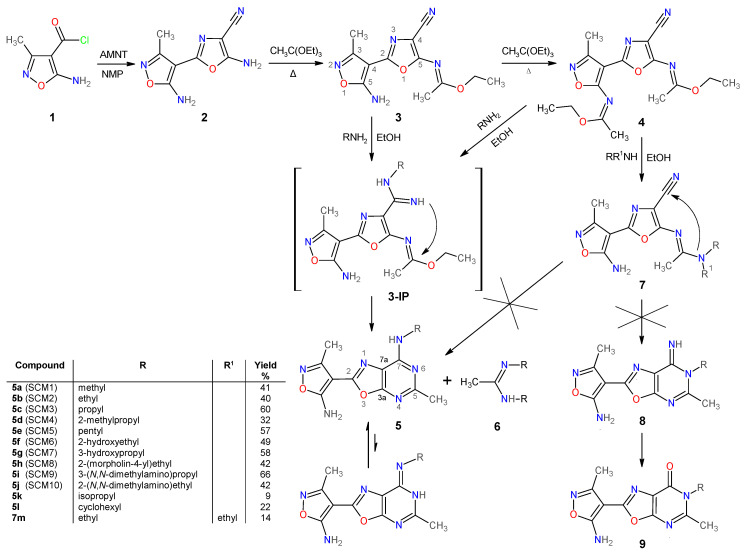
Transformations occurring during the synthesis of 7-aminooxazolo[5,4-*d*]pyrimidines **5**.

In general, there are two known main methods of synthesis of oxazolo[5,4-*d*]pyrimidines, namely, through cyclization of the pyrimidine ring on existing, appropriately substituted, oxazole derivative or through cyclization of the oxazole ring on pyrimidine derivative [26]. For the first time, this system was synthesized just by using the second method [1], where 2-mercapto-5-benzoylamino-4-hydroxypyrimidine was cyclized with phosphoryl trichloride (POCl_3_). Herein, the described series of new oxazolo[5,4-*d*]pyrimidines **5** is synthesized according to the synthetic pathway outlined in Scheme 1.

The following reagents and substrates have been used in the synthesis of the considered compounds: starting materials, such as esters of 2-cyano-3-alkoxy-2-butenoate acids, which were used to synthesize known 5-amino-3-methyl-4-isoxazolecarboxylic acid [27], were prepared according to the highly efficient methods precisely described in [28]. 5-Amino-3-methylisoxazole-4-carbonyl chloride (**1**, Scheme 1) was also prepared using the method described earlier [12]. Similarly to the method described by Freeman and Kim [29], 5-amino-2-(5-amino-3-methylisoxazol-4-yl)-oxazole-4-carbonitrile (**2**) was obtained in the reaction, which was conducted in 1-methyl-2-pyrrolidinone (NMP) at room temperature, between the commercially available aminomalononitrile tosylate (AMNT) and compound **1**. Then, ethyl *N*-{4-cyano-2-[5-(1-ethoxyethylidene)amino-3-methylisoxazol-4-yl]-oxazol-5-yl}ethanimidate (**4**) was obtained through the intermediate imidate, i.e., ethyl *N*-[2-(5-amino-3-methylisoxazol-4-yl)-4-cyanooxazol-5-yl]ethanimidate (**3**), by heating of oxazole **2** in an excess of triethyl orthoacetate under reflux. It is necessary to emphasize that the compound **3** has one and the product **4** has two centers of *Z-E* isomerism, respectively, so there is a possibility that, theoretically, two in the case of **3** or four single isomers in the case of **4** or their mixtures can be created, respectively. Anyway, a spectral analysis revealed that only one isomer was formed in both cases for the compounds **3** and **4**. We have not determined which one, as this fact seems to be of little importance for a course of the next stage of the synthesis. The reactions of the imidate **4** with a 30% ethanolic solution of the appropriate aliphatic amines, conducted at room temperature, resulted in a series of final oxazolo[5,4-*d*]pyrimidines **5**. It should be noted that the molecule of compound **4** has three reactive centers with lowered electron density that are susceptible to attack by nucleophilic agents, the nitrile carbon and the carbons of both imidate moieties. Therefore, three possible competitive reactions can occur and result in various products. Our research indicates that the desired compounds **5** form via the labile intermediate amidines **3-IP** that are formed by the nucleophilic addition of the corresponding amine to the nitrile group of the compound **4** or **3**. In this stage, an excess of the primary aliphatic amine as a strong nucleophile agent causes the removal of the ethoxyethylidene moiety (in the case of the compound **4**) from the amino group of the isoxazole ring with simultaneous cyclization to the pyrimidine ring, while acetamidines **6** (only in the case of primary amines) are produced as by-products. However, secondary aliphatic amines (such as *N,N*-diethylamine, R, R^1^ = Et) did not give the expected products **5** under these conditions but *N′*-cyanooxazolylacetamidine **7** (**7m**, R,R^1^ = Et for *N,N*-diethylamine) in low yield (≈14%) and the mixture of product of the decomposition of the compound **4**, i.e., the compounds **2** and **3**. It is worth mentioning that the ^1^H-NMR spectrum (recorded at 297.8 K ≈ 25 °C) of *N′*-cyanooxazolylacetamidine **7m** (for a detailed description of its spectrum, see Section 3.1.2 of “Preparation and experimental properties of compounds **2**–**5**, **7**” and for the spectrum, see Figure S76 in the Supplementary Material) shows that the corresponding protons of the *N*-ethyl groups are not magnetically equivalent. This suggests that inhibition of rotation around the bond between the quaternary acetamidine carbon atom and the nitrogen atom occur. This supposition has been supported with the theoretically predicted rotational barrier that has been estimated to be about 23 kcal·mol^−1^ (see Section 3.1.4. Computational details). A similar energy barrier is needed for free rotation of the *N,N*-dimethyl group in a known useful aprotic polar solvent, *N,N*-dimethylformamide (DMF), for which the experimentally estimated energy barrier is about 21 kcal·mol^−1^ [30]. In DMF, this inhibition of rotation is caused by fact that bond between carbonyl carbon and nitrogen has partial double bond character, which is connected with a significant contribution of the imidic acid tautomer to the resonance structure. In the case of *N′*-cyanooxazolylacetamidine **7m**, the reason could be similar.

Primary aliphatic amines with rigid bulky steric hindrance (with a secondary aliphatic radical like isopropyl or cyclohexyl) usually gave the mixture of products (like **2, 5** and **6**) with very low abundance of the oxazolo[5,4-*d*]pyrimidines **5**. In the cases of isopropylamine and cyclohexylamine, the yield of appropriate pure oxazolo[5,4-*d*]pyrimidines **5** was about 9% (**5k**) and 22% (**5l**), respectively. However, in the case of *t*-butylamine, only the products of decomposition of substrate **4** were isolated. Similarly, in the case of aromatic amines (such as aniline, R = Ph, R^1^ = H and *p*-toluidine, R = *p*-MePh, R^1^ = H), neither oxazolo[5,4-*d*]pyrimidines **5** nor *N′*-cyanooxazolyl-acetamidines **7** were observed to be formed. In these cases, the imidate **4** underwent only decomposition to the mixture of the compounds **3** and **2** under these conditions, so only the mixture of all of them was isolated. This is probably caused by the much less nucleophilicity of the aromatic amine nitrogen, making aromatic amines behave inertly under these reaction conditions.

It is necessary to underline that during the course of all the reactions of the imidate **4** with amines, the creation of some quantity of the substrate **2** and the intermediate imidate **3** was observed as a result of decomposition of the compound **4**. Besides, it appears that compound **4** is unstable in ethanolic solution and slowly decomposes through the intermediate imidate **3** to the substrate **2**. The isolated, from reaction mixture, intermediate imidate **3** has been also undergone the reactions with the appropriate aliphatic amines (like in the case of the imidate **4**) giving the same final oxazolo[5,4-*d*]pyrimidines **5** as in the case of the imidate **4**, which, in addition to spectral analysis, supports and chemically proves the molecular structure of the compound **3** (for example, preparation of the compound **5i**, see *Method C* in Section 3.1.2 “Preparation and experimental properties of compounds **2**–**5**, **7**”).

Theoretically predicted heats (enthalpies Δ_f_H_298_) of formation, opposite to binding energies, of *N′*-cyanooxazolylacetamidines **7** (see calculation details in 3.1.4. Computational details), are greater than the ones of structurally corresponding isomeric oxazolo[5,4-*d*]pyrimidines **5**, which means that oxazolo[5,4-*d*]pyrimidines **5** should be more thermodynamically stable than *N′*-cyanooxazolyl-acetamidines **7**. This points out that the observed cases of formation of *N′*-cyanooxazolylacetamidines **7** are rather kinetically and/or sterically controlled, especially taking into account that *N′*-cyanooxazolylacetamidines **7** were formed only in the case of reaction with secondary amines, like *N*,*N*-diethylamine, for example. Besides, the formation of *N′*-cyanooxazolylacetamidines **7** suggests that final 7-aminooxazolo[5,4-*d*]pyrimidines **5** can be produced through only unstable intermediate amidines **3-IP** that is formed as a result of the nucleophilic addition of appropriately active amine to cyano group of the imidate **4** or the intermediate imidate **3** because only these amidines **3-IP** can cyclize into oxazolo[5,4-*d*]pyrimidines **5** contrary to *N′*-cyanooxazolylacetamidines **7**. Theoretically, *N′*-cyanooxazolylacetamidines **7** could cyclize into *N*-6-substituted-oxazolo[5,4-*d*]pyrimidin-7-imines **8** but only in the cases where R^1^ = H (see Scheme 1). Formation of such similar imines **8** have been previously described by Ohtsuka [31]. However, imines **8** should be rather unstable, especially, in the presence of water (moisture) which can lead them to decompose into *N*-6-substituted-oxazolo[5,4-*d*]pyrimidin-7-ones **9**. Actually, the formation of imines **8** and/or oxazolo[5,4-*d*]pyrimidin-7-ones **9** was not observed under the applied reaction conditions during the conducted research. Besides, this fact is also supported on the basis of the theoretical research on the example of the compounds **5a** and **5c**, for a simulation carried out for single isolated molecule in a vacuum environment. The calculated difference of energy (ΔE_298_) between thermal-corrected energies of the considered compounds (**5a** and **5c**) and their respective isoforms (**8a** and **8c**) points out that the compounds **5a** and **5c** should be more thermodynamically stable by about 13 kcal·mol^−1^ (E_stab_ = −ΔE_298_ = 13.06 and 13.23 kcal·mol^−1^) than the theoretical compounds **8a** and **8c**, respectively (see calculation details in 3.1.4. Computational details). The calculations for simulated ethanol solutions of considered molecules conducted with the Conductor-like Polarizable Continuum Model (CPCM) [32] give similar results, i.e., E_stab_ = 12.43 and = 12.83 kcal·mol^−1^ for the compounds **5a** and **5c**, respectively. It can be seen that the electrostatic field generated by ethanol molecules has very little effect on the stabilization of the imino isomers **8** compared to vacuum. Finally, the 7-aminooxazolo[5,4-*d*]pyrimidine structure of the final products **5** were unquestionably proven by X-ray crystallography on the basis of single-crystal X-ray diffraction measurement of the compound **5h**.

It should be also noted that compounds **5** may exhibit amine-imine tautomerism (see Scheme 1), similar to purines and pyrimidine bases (e.g., adenine and cytosine), due to the mobility of the proton of the amino group at the seventh position of the oxazolopyrimidine ring. Therefore, there is probability that this proton may easily jump to nitrogen at the sixth position of the pyrimidine ring of the oxazolopyrimidine system due to the fact that the nitrogen atom of amino group at the seventh position, the nitrogen atom of the pyrimidine ring at the sixth position and the carbon atom at seventh position form a semi-cyclic amidine moiety. Theoretical estimation (on the example of the compound **5h**) of free enthalpy (ΔG_trans(298)_ = 10.748 kcal·mol^−1^) and the equilibrium constant (K_trans_ = [imine]/[amine] ≈ 1.323 × 10^−8^ proton transition as a result of amine-imine tautomerism suggests that the amine tautomer of the compound **5h** should dominate with a huge advantage in the state of thermodynamic equilibrium, which could have been expected because of partial loss of aromaticity by oxazolopyrimidine ring system of imine tautomer. It can be figured out that one molecule of imine tautomer of the compound **5h** occurs with over 75.5 million molecules of its amine tautomer in the equilibrium (see calculation details in 3.1.4. Computational details). For ethanol environment simulation, the results (ΔG_trans(298)_ = 10.752 kcal·mol^−1^ and K_trans_ ≈ 1.313 × 10^−8^) are very similar (insignificant difference) to those of a vacuum one what would suggests that that electric field generating by the solvent does not influence on the equilibrium in this case. Unfortunately, the CPCM calculations do not take into account the possibility that hydrogen bonds may form between the protic solvent, which is ethanol, and the molecules of the considered compounds, which is a disadvantage of this method.

Taking into account some chemical instability of the compound **4** and the formation of by-products (**2, 6** and **7**) during the reaction, the yields (calculated for purified products) of expected 7-aminooxazolo[5,4-*d*]pyrimidines **5** have ranged from about 9% (for isopropyl **5k** derivatives) to 66% (for 3-(*N,N*-dimethylamino)propyl derivative **5i**) depending on the primary aliphatic amine used. Since the compound **4** slowly decomposes in ethanol solution, reactions with some amines in aprotic carbon tetrachloride (CCl_4_) were also carried out (see *Method B* for the compound **5c** and **5i** in Section 3.1.2 “Preparation and experimental properties of compounds **2**–**5, 7”**). However, it appeared that the yields of final products **5** are much lower in this case. For example, for the crude compound **5i** (R = 3-(*N,N*-dimethylamino)propyl) and **5c** (R = propyl), the yield was only 31% and 13%, respectively, which indicates that CCl_4_ has proven to be a worse solvent than ethanol to carry out for these reactions. Therefore, research on the selection of optimal conditions for these reactions will be continued in order to obtain better yields. The structures of all the new compounds were proven by ESI-MS, IR, ^1^H, ^13^C-NMR and 2D NMR COSY spectroscopy, with single-crystal X-ray diffraction measurement (on the example of the compound **5h**) and, additionally, through appropriate chemical transformations. 2D NMR spectra were particularly useful in the unambiguous assignment of chemical shifts to the corresponding protons and to some quaternary carbon atoms. The investigated compounds **5** are highly melting (with decomposition) solids, insoluble in chloroform and water, slightly soluble in methanol and ethanol but well soluble in DMSO (at room temperature).

#### 2.1.2. Crystal Structure of the Compound **5h**

**Figure 2:**
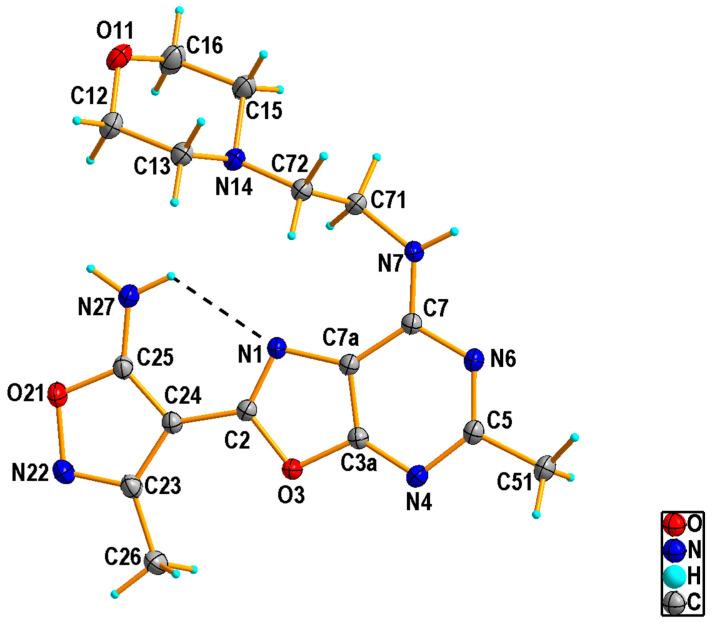
X-ray crystal structure of **5h**, with atom-numbering scheme. Displacement ellipsoids are drawn at the 30% probability level. H atoms are shown as small spheres of arbitrary radii.

The structure of **5h** was confirmed unambiguously by X-ray single-crystal analysis. Selected geometrical parameters and hydrogen bonds are given in Tables S25 and S26 (Supplementary Materials). **5h** crystallizes in the *P* $\bar{1}$ space group, with one molecule in the asymmetric unit. The molecular conformation of each molecule is stabilized by an intramolecular N—H···N hydrogen bond [N···N = 2.889 (2) Å] (Table S2), which closes six-membered ring (forming S(6) motif), as shown in Figure 2.

The oxazolo[5,4-*d*]pyrimidine system is essentially planar [r.m.s. deviation 0.007 Å] and forms a dihedral angle of 0.936(11)° with the 1,2-oxazole group [r.m.s. deviation 0.002 Å] attached to atom C2. Additionally, due to the quite short C2–C24 bond length [1.426(2) Å], one can conclude that the aromatic systems are conjugated. However, there was a major localization of the double bonds in the pyrimidine ring of the oxazolo[5,4-*d*]pyrimidine system. The C4–N3a bond length is 1.322(2) Å against 1.347(2) and 1.358(2) Å for the C5-N4 and C7–N6 bonds, respectively. This difference is also observed in the C–C bond distances [1.407(2) and 1.372(2) Å for C7–C7a and C3a–C7a, respectively]. These values in connection with the shortening of the C7-N7 bond [1.341(2) Å] may be associated with the partial aromaticity loss by the oxazolo[5,4-*d*]pyrimidine ring system. The morpholine ring adopts a chair conformation. The orientation of the chain substituent attached to the atom C7 with respect to the plane defined by oxazolo[5,4-*d*]pyrimidine ring system may be reflected in C71-N7-C7-C7a and C7-N7-C71-C72 torsion angles of −9.0(3)° and 86.5(2)°, respectively.

**Figure 3:**
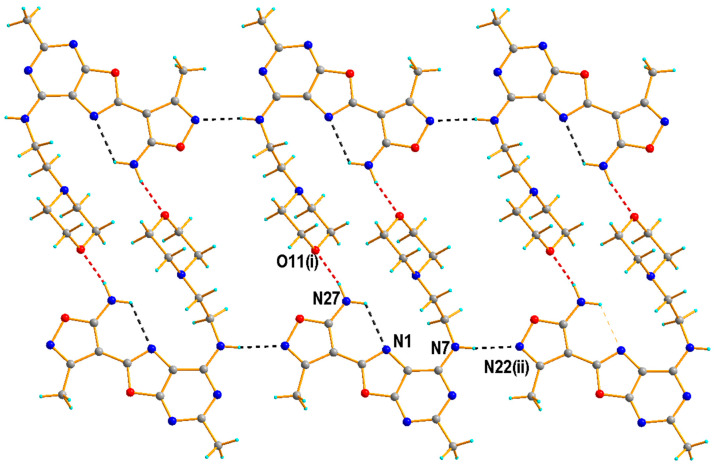
Crystal packing diagram of **5h**, showing dominating N—H···N and N—H···O hydrogen bond interactions, viewed along the ***a*** axis. (The dashed lines indicates N—H···N (black) and N—H···O (red) hydrogen bonds. Symmetry codes: (i) −x, −y + 1, −z; (ii) x + 1, y−1, z.).

The packing of **5h** is mainly dictated by N—H···O and N—H···N hydrogen bonds (Table S2). The nitrogen atom (N27) from the amine group acts as hydrogen-bond donor via H atoms to the oxygen atom (O11) from the morpholine ring of adjacent molecules, forming the N27—H27···O11^i^ hydrogen bond of 2.889(2) Å [symmetry code: (i) −*x*, −*y* + 1, −*z*], which leads to a dimer formation. On the other hand, molecules are linked through N7—H7···N22^ii^ hydrogen bond of 2.959(3) Å [symmetry code: (ii) *x* + 1, *y* − 1, *z*] between the second amine group as a donor and the nitrogen atom of the 1,2-oxazole ring as an acceptor. The combination of these interactions results a ladder-like chain structure (Figure 3).

### 2.2. Biological Activity of Some New 7-Aminooxazolo[5,4-d]Pyrimidines ***5a***–***5j*** (***SCM1***–***10***)

The ten compounds **5a**–**5j** subjected to biological testing have been labelled as **SCM1**–**10** (see Scheme 1).

#### 2.2.1. Effects of Tested Compounds on Viability of A-549 Reference Cell Line, Lymphocyte Proliferation and Growth of Tumor Cell Lines

##### Estimation of Cytotoxicity of Tested Compounds on A-549 Reference Cell Line

Selection of therapeutically useful compounds, in order that they could be potential drugs, depends not only on their significant biological actions but also on bioavailability and possibly lowest cytotoxic properties. The compounds were therefore evaluated for their cytotoxic action by application of a reference A-549 cell line at a concentration range of 62.5–15.6 µM using respective dilutions of the solvent (DMSO) in the culture medium as control cultures (Table 1).

##### Influence of the Tested Compounds on Lymphocyte Proliferation

The ability to suppress mitogen-induced lymphocyte proliferation by the compounds **SCM1**–**10** was evaluated in the model of PHA-induced proliferation of human PBMC, in which the compounds exhibited differentiated suppressive activity with best results for **SCM5** and **SCM9** (Table 2). At concentration of 50 µM, the suppressive acitivity of **SCM5**, **SCM9** and teriflunomide were 69.8%, 60.7% and 37.9%, respectively. Therefore, these two compounds with strongest suppressive effects with regard to PHA-induced PBMC proliferation at a lower concentration range were selected for the subsequent more advanced experiments.

**Figure 4:**
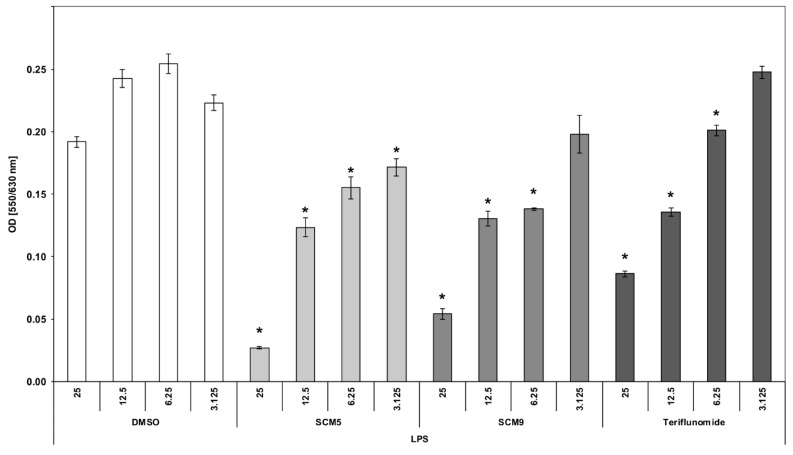
The effect of **SCM** compounds on LPS-induced mouse splenocyte proliferation. The compounds were added to the cultures at concentrations of 25, 12.5, 6.25 and 3.125 μM, and LPS (50 μg/mL) as inducer of cell proliferation. After 3-day incubation, the cell proliferation was determined using a colorimetric MTT assay. Teriflunomide served as a reference drug. The results are presented as the OD values from quadruplicate determinations (mean ±SE). * indicates statistical significance when *p* < 0.05, versus DMSO control.

The tested compounds **SCM5** and **SCM9** appeared to be even more efficient in inhibition of LPS-stimulated mouse splenocytes (Figure 4) which reflects B cell proliferation [33]. This suppressive effect was more profound in comparison with that one of teriflunomide.

##### Influence of the Tested Compounds on Growth of Tumor Cell Lines

To evaluate potential antitumor activity of the compounds, A-431 epidermal, HT-29 colon and L-1210 leukemia cell lines (Table 3) were used. Cisplatin was the reference anticancer drug in the study. The results showed that the compounds displayed differential ability to inhibit growth of these cell lines. The best results were observed for HT-29 cell line where **SCM9** caused (still significant) 30% growth inhibition at a concentration of 12.5 μM, albeit the activity was weaker than the one of reference cisplatin.

#### 2.2.2. Effect of the Compounds on TNF α Production by LPS-Stimulated Human Whole Blood Cultures

Testing the ability of the compounds to inhibit LPS-induced TNF α production in the human whole blood culture (Table 4) revealed that **SCM5** was inactive whereas **SCM9** demonstrated dose-dependent suppression with a significant inhibition only at 25 μM concentration.

#### 2.2.3. Antiviral Properties of the Compounds

**Figure 5:**
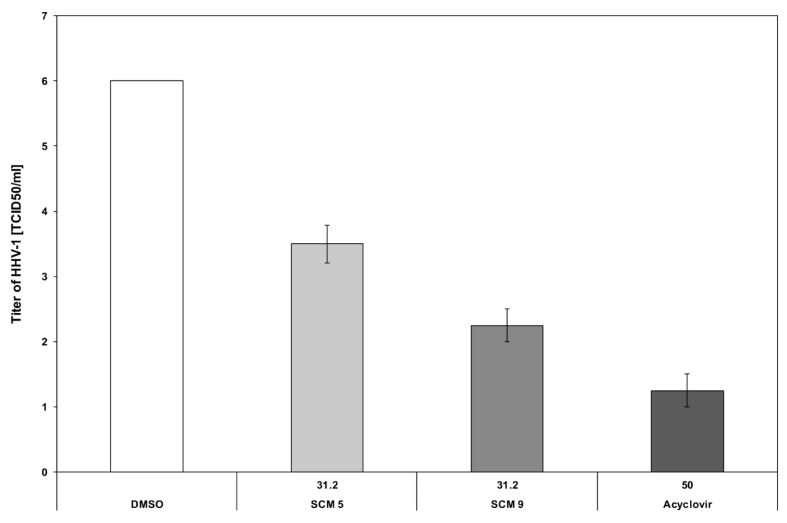
The virus-inhibiting properties of **SCM** compounds against human HHV-1 in A-549 cell line.

The next aim of this study was to examine the antiviral activity of **SCM5** and **SCM9** in vitro against HHV-1. The treatment of A-549 cells infected with HHV-1 with **SCM** compounds resulted in significant reductions in virus yields, reaching up to a 3.5-log_10_ decrease for **SCM5** and a 2.2-log_10_ decrease for **SCM9** in comparison with appropriate DMSO control as it is shown in Figure 5. A 4-log_10_ reduction of virus titer demonstrates the ability of the tested compounds to inactivation of viruses to a level acceptable by the European Standards. In addition, **SCM9**, and to a lesser degree **SCM5**, significantly enhanced the viability of A-549 cells infected with the virus (data are not shown).

A-549 cells were infected with HHV-1 and after 1h of virus absorption at 37 °C, inoculum of virus was removed and the infected cells were incubated for 48 h with **SCM5**, **SCM9** compounds (31.2 µM) and DMSO as the solvent control. Acyclovir (50 µM) was used as a reference drug. The viral titer was expressed with reference to the TCID_50_ value, which is based on the cytopathic effect caused by this virus in approximately 50% of infected cells. The results, which were obtained from quadruplicate determinations (mean ±SE), are presented as the log_10_ TDIC50/mL values in relation with appropriate DMSO control. The level of virus was defined according to the requirements presented in PN-EN 14476:2013+A1:2015.

#### 2.2.4. Influence of the Compounds on Expression of Signaling Molecules in Cell Lines

The researched compounds were tested for their influence on expression of signaling molecules playing roles in apoptosis in Jurkat T cell model. The expression of the following signaling proteins was analyzed: caspases3, 7, 8, 9, Bcl-2, Fas, NFκB and p53. Of interest, the compounds did not alter expression of these proteins except one instance, namely, **SCM5** caused a complete block in Bcl-2 expression (data are not shown). Since the expression of MAP kinases may also predict a fate of cells (activation, differentiation or apoptosis) the expression of ERK, JNK and p38 in Jurkat cells have been we additionally measured. The results (Table 5) show that the compounds have differently affected expression of signaling molecules. **SCM5** strongly suppressed the expression of ERK1, ERK2, JNK and to a lesser degree p38 subunits. On the other hand, the compound **SCM9** did not significantly affect the expression of ERK, but elevated the expression of JNK, p38α and p38β.

The determination of MAP kinase expression in WEHI-231 cells, which were exposed to the compounds, revealed no significant changes in the case of **SCM9** with except 2-fold increase in the level of p38δ (Table 6). On the other hand, **SCM5** caused a strong decrease in expression of ERK-1, ERK-2 and to a lesser degree of JNK and p38 subunits. No significant changes were also observed with regard to MAP kinase expression in WEHI 213 cells in the case of **SCM9** apart from 2× increase in p38δ (Table 6). More marked changes were noted in the case of **SCM5** which increased the expression levels of p38γ (2.7×) and p38δ (3.7).

In contrast, more significant changes in expression of signaling molecules, associated with apoptosis, were found in WEHI-231 cells incubated with **SCM5**. The changes (Table 7) included strong elevations in expression of caspase 8 (8×), caspase 9 (23×), NFκB (17.9×), Bcl-2 (10×) and Fas (8.8×). **SCM9**, on the other hand, did not significantly change the expression of these molecules.

The results, presented above, revealed in vitro immunosuppressive activity of the selected compounds (**SCM5** and **SCM9**), which was, amongst the other, manifested by inhibition of cell proliferation and tumor cell growth. Both compounds exhibited the stronger antiproliferative effect on B cells in the mouse model than on T cells in the human peripheral blood cell population. Interestingly, only the compound **SCM9** inhibited LPS-induced TNF α production which suggests its anti-inflammatory character. The changes in the expression of signaling molecules by **SCM9** in Jurkat cells, such as 2× increase in JNK and an increase in p38α may indicate a proapoptotic action of the compound towards T cells associated with changes in MAP kinase expression [34,35]. However, it is intriguing that the signaling pathways induced by the compound did not involve caspase, NFκB, BcL-2 and Fas pathway despite very strong immunosuppressive properties of **SCM9** towards WEHI-231. It is possible that the suppressive and anti-inflammatory action of **SCM9** may engage still another signaling pathway, which could not be identified by the limited application of signaling molecules used in this study. In the case of **SCM5**, its immunosuppressive mechanism of action is more much easier to explain taking into consideration elicitation of signaling pathways by the compound. Firstly, the compound abrogated cell activation as evidenced by a strong, although differential decrease of expression of all MAP kinases, such as: ERK1 and ERK2, JNK and p38 subunits, which concerted involvement in cell differentiation and activation is essential [36]. More importantly, in the model of WEHI-231 cells, the compound induced strong amplification in expression of all molecules involved in apoptosis such as: caspase 8 [37], caspase 9 [38], NFκB [37,38], Bcl-2 [39] and Fas [40]. The level of cytotoxicity against A-549 cells at concentration of 62.5 µM is in accordance with our results on cell signaling showing that the proapoptotic action of **SCM5** is more evident than that of **SCM9**. Of interest, the antiproliferative activity of both compounds on B cells, was stronger than this one observed with PBMC (predominantly T cells), since it was also high at lower concentration. For example, **SCM5** at 25 µM/mL concentration, inhibited PBMC and splenocyte proliferation by 28% and 81.5%, respectively. This phenomenon may be of importance when potential therapeutic applications of the compounds are considered, for example in autoimmune disorders, particularly involving B cells and in neoplastic diseases. The ability of the compounds to interfere with the virus replication would require further research in terms of mechanism of action. Interestingly, stimulation of expression of p38 γ and p38 δ in WEHI 231 cells by the compounds may have significance in inhibition of virus replication [41]. Such properties, comparable to that of Acyclovir^®^, may enable application possibility of **SCM9** as a potential antiviral drug on condition further advanced and positive pharmacological research.

The preliminary analysis of structure/activity relationship of the considered compounds **5a**–**5j** (**SCM1-10**) led us to the following general conclusions. Changes of substituents at position 7 in the structure of 7-aminooxazolo[5,4-*d*]pyrimidines **5** have resulted in varying degrees of biological activity. Amongst all the compounds that were biologically tested, the most active compounds, **5e** (**SCM5**) and **5i** (**SCM9**), have lipophilic chains with five linearly arranged non-hydrogen atoms, i.e., 7*-N*-pentyl and 7-(*N,N*-dimethylamino)propyl, respectively. In the opposition, there are the compounds with more hydrophilic substituents, such as 2-(morpholin-4-yl)ethyl in a molecule of the compound **5h** (**SCM8**) or hydroxylalkyl ones in a molecule of the compounds **5f** (**SCM6**) and **5g** (**SCM7**), that showed no significant biological activity in the conducted tests.

## 3. Experimental

### 3.1. Chemistry

#### 3.1.1. Materials and Methods

Commercially available reagents, i.e., aminomalononitrile tosylate (AMNT), triethyl orthoacetate and aliphatic amines were purchased from following suppliers such as TCI (Tokyo, Japan) and Sigma-Aldrich (Merck Group, Darmstadt, Germany), and were used without further purification. Thin layer chromatography (TLC) analyses were conducted using Alugram SIL G/UV 254nm plates (Macherey-Nagel, Düren, Germany) and visualized by ultraviolet (UV) light at 254 nm (UV A. KRÜSS Optronic GmbH, Hamburg, Germany). Column chromatography was carried out using Silicagel 60 and the solvent system of eluent was composed of chloroform and ethyl acetate (9:1 or 1:1) or chloroform and methanol (9:1). Melting points were measured on uniMELT 2 apparatus (LLG, Meckenheim, Germany) and were uncorrected. Electrospray Ionization Mass Spectroscopy (ESI-MS) spectra were recorded with a compact^TM^ Electrospray Ionisation-Quadrupole-Time of Flight (ESI-Q-TOF) apparatus (Bruker Daltonik GmbH, Bremen, Germany). All compounds for ESI-MS experiments were dissolved in methanol and detected as positive ions. Theoretical monoisotopic masses of ions were calculated (calc.) using Bruker Compass Data Analysis 4.2 software (Bruker Daltonik GmbH, Bremen, Germany). The Attenuated Total Reflectance IR (ATR-FT-IR) spectra (4000–450 cm^−1^) were recorded on a Nicolet iS50 FT-IR spectrophotometer (Thermo Fisher Scientific Inc., Waltham, MA, USA). using clean solid forms of the compounds (on the diamond crystal surface, 32 scans, resolution: 1 cm^−1^, measurement temperature: 20–25 °C). IR Spectra were recorded with ATR intensity correction. It is known, that the relative intensity of bands in an ATR spectrum increases with wavelength causing the distortion of relative peak intensities in comparison with the classical transmission experiment [42]. Omnic Specta software (Thermo Fisher Scientific Inc., Waltham, MA, USA) was used for IR spectra analysis. Baselines of all IR spectra were corrected with autocorrection (fit order 2) and then all the spectra were normalized. ^1^H-NMR (300.15 MHz), ^13^C-NMR (75.47 MHz, broadband full decoupling method), ^1^H-^1^H COSY (300.15 MHz), ^1^H-^13^C 2D COSY NMR (channel F2: 300.15 MHz, channel F1: 75.47 MHz), 2D ^1^H-^13^C HSQC (Heteronuclear Single Quantum Coherence = Heteronuclear Single Quantum Correlation, channel F2: 300.15 MHz, channel F1: 75.47 MHz) and 2D ^1^H-^13^C HMBC (Heteronuclear Multiple Bond Correlation) spectra were recorded using 5 mm tubes and concentration about 20 mg of tested compounds in 0.6 mL (≈90–130 mM solutions) of appropriate deuterated solvent (such as dimethyl sulfoxide (DMSO-d_6_)_,_ chloroform (CDCl_3_) or methanol (CD_3_OD) with a Bruker ARX-300 spectrometer (Bruker Analytische Messtechnik GmbH, Rheinstetten, Germany). 2D Spectra enabled the unambiguous assignment of chemical shifts to the corresponding protons and to some quaternary carbon atoms. The reported data contain chemical shift in parts per million (ppm) units, signal multiplicities (abbreviations used: br—broad, s—singlet, d—doublet; t—triplet, q—quartet, m—multiplet), and the number of protons. The values of coupling constant were reported as *J* in Hz. All the NMR, IR and MS measurements were carried out in the Laboratory of Elemental Analysis and Structural Research, Faculty of Pharmacy, Wroclaw Medical University.

#### 3.1.2. Preparation and Experimental Properties of Compounds **2**–**5** and **7**

##### *5-Amino-2-(5-Amino-3-Methyl-Isoxazol-4-yl)-Oxazole-4-Carbonitrile* (**2**)

The reaction was carried out according to the protocol described by Freeman and Kim with slight modifications [29]. A solution of aminomalononitrile tosylate (AMNT, 0.8 g, 3.2 mmol) in 1-methyl-2-pyrrolidinone (NMP, 8.0 mL) was stirred with freshly prepared 5-amino-3-methylisoxazole-4-carbonyl chloride (1.02 g, 6.4 mmol, 2 equiv) at room temperature for 7 days. The course of the reaction was controlled by TLC. The reaction was conducted as long as the starting reagent AMNT was present. Then, the final mixture was cooled to 5–10 °C and diluted with ice-cold water. The formed precipitate was filtered off and dried to give the crude compound **2** as a purple solid that was crystallized from methanol. Yield 0.306 g, 47.2%; m.p. 235–237 °C (dec.); HR-ESI-MS: *m/z*[u/e] calc. for C_8_H_7_N_5_O_2_Na [M + Na]^+^ 228.0491, found 228.0499 (see Figure S1 in the Supplementary Material); ATR- FTIR: ν_max_ (cm^−1^) 3369, 3159 (NH_2_), 2210 (CN), 1636, 1592, 1534, 1488, 1062 (see Figure S24 in the Supplementary Material); ^1^H-NMR (DMSO-d_6_, 300 MHz): δ (ppm) 2.24 (s, 3H, methyl group of isoxazole ring), 7.40 (brs, 2H, -NH_2_), 7.58 (brs, 2H, -NH_2_) (see Figure S22 in the Supplementary Material); ^13^C-NMR (DMSO-d_6_, 75.5 MHz): δ (ppm) 11.3, 81.2, 82.4, 115.8, 145.3, 157.1, 160.7, 168.2 (see Figure S23 in the Supplementary Material).

##### *Ethyl N-{4-Cyano-2-[5-(1-Ethoxyethylidene)Amino-3-Methylisoxazol-4-yl]-Oxazol-5-yl}Ethanimidate* (**4**)

Compound **3** (2.54 g, 7.35 mmol) and triethyl orthoacetate (32 mL) were heated with stirring at boiling temperature (145 °C) for 15 h. On completion of the reaction as monitored by TLC, the excess of triethyl orthoacetate was evaporated under vacuum. The crude product was purified by chromatography on silica gel (CHCl_3_:EtOAc, 9:1) to give compound **4** as a yellow solid. Yield 3.93 g, 91.9%; m.p. 81–83 °C (dec.); ESI-MS: *m/z*[u/e] calc. for C_16_H_20_N_5_O_4_ [M + H]^+^ 346.1510, found 346.1445 (see Figure S3 in the Supplementary Material); ATR-FTIR: ν_max_ (cm^−1^) 2994, 2231 (CN), 1659, 1635, 1464, 1378, 1298, 1043 (see Figure S30 in the Supplementary Material); ^1^H-NMR (CDCl_3_, 300 MHz): δ (ppm) 1.37 (t, ^3^*J* = 7 Hz, 3H, methyl of ethoxyl group of ethanimidate moiety in isoxazole ring), 1.39 (t, ^3^*J* = 7 Hz, 3H, methyl of ethoxyl group of ethanimidate moiety in oxazole ring), 2.10 (s, 3H, methyl group of ethanimidate moiety in isoxazole ring), 2.24 (s, 3H, methyl group of ethanimidate moiety in oxazole ring), 2.50 (s, 3H, methyl group of isoxazole ring), 4.36 (q, ^3^*J* = 7 Hz, 2H, methylene of ethoxyl group of ethanimidate moiety in isoxazole ring); 4.37 (q, ^3^*J* = 7 Hz, 2H, methylene of ethoxyl group of ethanimidate moiety in oxazole ring) (see Figure S28 in the Supplementary Material). ^13^C-NMR (CDCl_3_, 75.4 MHz): δ (ppm) 12.42, 13.84, 13.87, 19.20, 19.24, 64.07, 64.31, 93.58, 100.07, 113.70, 150.62, 158.30, 159.49, 168.14, 168.87, 169.10 (see Figure S29 in the Supplementary Material).

##### *Ethyl N-[2-(5-Amino-3-Methylisoxazol-4-yl)-4-Cyanooxazol-5-yl]Ethanimidate* (**3**)

Method A (in ethanol)

Compound **4** (0.3 g, 0.87 mmol) and ethanol (6 mL) were heated with stirring at boiling temperature (78 °C) for 26 h. On completion of the reaction as monitored by TLC, the ethanol was evaporated under vacuum. The crude product was purified by chromatography on silica gel to give the compound **3** as the beige solid. Yield 0.037 g, 15.5%; m.p. 181–182 °C (dec.); ESI-MS: *m/z*[u/e] calc. for C_12_H_13_N_5_O_3_Na [M + Na]^+^ 298.0911, found 298.0975 (see Figure S2 in the Supplementary Material); ATR-FTIR: ν_max_ (cm^−1^) 3389, 3129, 2992, 2230 (CN), 1640, 1611, 1382, 1294, 1032, 953, 744 (see Figure S27 in the Supplementary Material); ^1^H-NMR (CDCl_3_, 300 MHz): δ (ppm) 1.38 (t, ^3^*J* = 7.1 Hz, 3H, methyl of ethoxyl group of ethanimidate moiety in the oxazole ring), 2.27 (s, 3H, methyl group of ethanimidate moiety in the oxazole ring), 2.41 (s, 3H, methyl group of isoxazole ring), 4.36 (q, ^3^*J* = 7.1 Hz, 2H, methylene of ethoxyl group of ethanimidate moiety in the oxazole ring), 6.03 (s, 2H, -NH_2_ of isoxazole ring) (see Figure S25 in the Supplementary Material); ^13^C-NMR (CDCl_3_, 75.5 MHz): δ (ppm) 11.7, 13.9, 19.3, 64.3, 83.3, 99.2, 113.7, 152.4, 157.4, 157.5, 168.1, 168.6 (see Figure S26 in the Supplementary Material).

##### *2-(5-Amino-3-Methylisoxazol-4-yl)-7-N-Methylamino-5-Methyloxazolo[5,4-d]Pyrimidine* (**5a, SCM1**)

Method A (in ethanol)

To compound **4** (0.2 g, 0.58 mmol), a 30% solution of methylamine in ethanol (1 mL) was added in one portion. The reaction mixture was cooled to 5–10 °C and stirred for 0.5 h. When the reaction was complete, as shown by TLC, the precipitate formed was filtered off, dried and crystallized from ethanol. The compound **5a** (0.062 g, 41.0%) was obtained as a white crystals: m.p. 246–247 °C (dec.); HR-ESI-MS: *m/z*[u/e] calc. for C_11_H_13_N_6_O_2_ [M + H]^+^ 261.1094, found 261.1081 (see Figure S4 in the Supplementary Material); ATR-FTIR: ν_max_ (cm^−1^) 3365, 3300, 3120, 1662, 1617, 1532, 1322, 1200, 1054 (see Figure S34 in the Supplementary Material); ^1^H-NMR (DMSO-d_6_, 300 MHz): δ (ppm) 2.34 (s, 3H, methyl group of isoxazole moiety), 2.50 (s, 3H, signal covered with solvent, methyl group at position 5 of the oxazolopyrimidine system), 3.53 (s, 3H, *N*-methyl group), 7.26 (brs, 1H, -NH), 7.87 (brs, 2H,-NH_2_) (see Figure S31 in the Supplementary Material); ^13^C-NMR (DMSO-d_6_, 75.5 MHz): δ (ppm) 11.3 (carbon of methyl group of the isoxazole moiety), 23.4 (carbon of methyl group at position 5 of the oxazolopyrimidine system), 31.9 (carbon of *N*-methyl group at position 7 of the oxazolopyrimidine system), 81.6 (quaternary carbon C4 of the isoxazole ring), 116.7 (quaternary bridgehead carbon C7a of the oxazolopyrimidine system), 153.6, 154.4, 154.9, 157.0 (quaternary carbon C5 of the oxazolopyrimidine system), 157.2 (quaternary carbon C3 of the isoxazole ring), 169.2 (quaternary carbon C5 of the isoxazole ring) (see Figure S32 in the Supplementary Material). ^1^H-^13^C 2D COSY NMR spectrum of **5a** (see Figure S33 in the Supplementary Material).

##### *2-(5-Amino-3-Methylisoxazol-4-yl)-7-N-Ethylamino-5-Methyloxazolo[5,4-d]Pyrimidine* (**5b, SCM2**)

Method A (in ethanol)

To compound **4** (0.2 g, 0.58 mmol), a 30% solution of ethylamine in ethanol (1 mL) was added in one portion. The reaction mixture was cooled to 5–10 °C and stirred for 0.5 h. When the reaction was complete, as shown by TLC, the formed precipitate was filtered off, dried and crystallized from ethanol. The compound **5b** (0.063 g, 39.6%) was obtained as a white crystals: m.p. 245–245.5 °C (dec.); HR-ESI-MS: *m/z*[u/e] calc. for C_12_H_15_N_6_O_2_ [M + H]^+^ 275.1251, found 275.1246 (see Figure S5 in the Supplementary Material); ATR-FTIR: ν_max_ (cm^−1^) 3410, 3084, 1669, 1639, 1533, 1316, 1169, 1040 (see Figure S38 in the Supplementary Material); ^1^H-NMR (DMSO-d_6_, 300 MHz): δ (ppm) 1.24 (t, ^3^*J* = 7.0 Hz, 3H), 2.33 (s, 3H, methyl group of isoxazole moiety), 2.55 (s, 3H, methyl group at position 5 of the oxazolopyrimidine system), 4.17 (q, ^3^*J* = 6.9 Hz, 2H, *N*-methylene group of the ethyl chain), 7.23 (brs, 1H, -NH), 7.86 (brs, 2H, -NH_2_) (see Figure S35 in the Supplementary Material); ^13^C-NMR (DMSO-d_6_, 75.5 MHz): δ (ppm) 11.4, 12.2, 22.8, 39.8 (signal covered with solvent, carbon of *N*-methylene group of ethyl chain at position 7 of the oxazolopyrimidine system), 81.6 (quaternary carbon C4 of the isoxazole ring), 117.0 (quaternary bridgehead carbon C7a of the oxazolopyrimidine system), 153.4, 153.8, 155.1, 156.3 (quaternary carbon 5 of the oxazolopyrimidine system), 157.2 (quaternary carbon C3 of the isoxazole ring), 169.4 (quaternary carbon C5 of the isoxazole ring) (see Figure S36 in the Supplementary Material). ^1^H-^13^C 2D HSQC spectrum of **4b** (see Figure S37 in the Supplementary Material).

##### *2-(5-Amino-3-Methylisoxazol-4-yl)-7-N-Propylamino-5-Methyloxazolo[5,4-d]Pyrimidine* (**5c, SCM3**)

Method A (in ethanol)

To compound **4** (0.2 g, 0.58 mmol), a 30% solution of propylamine in ethanol (1 mL) was added in one portion. The reaction mixture was cooled to 5–10 °C and stirred for 1 h. Then, the mixture was warmed to room temperature and maintained with stirring for 1.5 h. When the reaction was complete, as shown by TLC, the formed precipitate was filtered off, dried and crystallized from ethanol. The compound **5c** (0.104 g, 60.5%) was obtained as a white solid: m.p. 209–210 °C (dec.); HR-ESI-MS: *m/z*[u/e] calc. for C_13_H_17_N_6_O_2_ [M + H]^+^ 289.1408, found 289.1407 (see Figure S6 in the Supplementary Material); ATR-FTIR: ν_max_ (cm^−1^) 3397, 3306, 2979, 1684, 1662, 1617, 1599, 1443, 1353, 1181, 1043, 792 (see Figure S41 in the Supplementary Material); ^1^H-NMR (DMSO-d_6_, 300 MHz): δ (ppm) 0.93 (t, ^3^*J_32_* = 7.3 Hz, 3H, terminal chain methyl), 1.69 (m, ^3^*J_23_* = 7.3 Hz, ^3^*J_21_* = 7.8 Hz, 2H, middle chain methylene), 2.33 (s, 3H, methyl group of the isoxazole moiety), 2.54 (s, 3H, methyl group at position 5 of the oxazolopyrimidine system), 4.05 (t, ^3^*J_12_* = 7.8 Hz, 2H, chain methylene connected with amine nitrogen at the 7th position), 7.25 (brs, 1H, -NH), 7.87 (brs, 2H, -NH_2_) (see Figure S39 in the Supplementary Material); ^13^C-NMR (DMSO-d_6_, 75.5 MHz): δ (ppm) 11.03 (carbon of terminal methyl of propyl chain at position 7 of the oxazolopyrimidine system), 11.39 (carbon of methyl group of the isoxazole moiety), 19.91 (carbon of middle methylene group of propyl chain at position 7 of the oxazolopyrimidine system), 22.95 (carbon of methyl group at position 5 of the oxazolopyrimidine system), 46.28 (carbon of *N*-methylene group of the propyl chain), 81.58 (carbon C4 of the isoxazole ring), 117.06 (quaternary bridgehead carbon C7a of the oxazolopyrimidine system), 153.70, 153.75, 154.84, 156.38 (quaternary carbon C5 of the oxazolopyrimidine system), 157.23 (quaternary carbon C3 of the isoxazole ring), 169.33 (quaternary carbon C5 of the isoxazole ring) (see Figure S40 in the Supplementary Material).

Method B (in tetrachloromethane)

To compound **4** (0.14 g, 0.40 mmol), a 30% solution of propylamine in CCl_4_ (1 mL) was added in one portion. The reaction mixture was cooled to 5–10 °C and stirred for 1 h. Then, the mixture was warmed to room temperature and maintained with stirring for 47 h. When the reaction was complete, as shown by TLC, the formed precipitate was filtered off. The solution after filtration was evaporated in a vacuum and the residue was dilution with ethanol. The formed precipitate was filtered off and dried to give the compound **5c** as a white solid (0.015 g, 12.8%): m.p. 208–209 °C (dec.); ESI-MS: *m/z*[u/e] calc. for C_13_H_17_N_6_O_2_ [M + H]^+^ 289.1408, found 289.1454 (see Figure S7 in the Supplementary Material). Other spectral analyzes (IR, NMR) confirm that the product is identical to that obtained by Method A.

##### *2-(5-Amino-3-Methylisoxazol-4-yl)-7-N-(2-Methylpropyl)Amino-5-Methyloxazolo[5,4-d]Pyrimidine* (**5d, SCM4**)

Method A (in ethanol)

To compound **4** (0.2 g, 0.58 mmol), a 30% solution of 2-methylpropylamine in ethanol (1 mL) was added in one portion. The reaction mixture was cooled to 5–10 °C and stirred for 1 h. Then, the mixture was warmed to room temperature and maintained with stirring for 3.5 h. When the reaction was complete, as shown by TLC, the formed precipitate was filtered off, dried and crystallized from ethanol. The compound **5d** (0.057 g, 32.5%) was obtained as a white solid: m.p. 183–184 °C (dec.); HR-ESI-MS: *m/z*[u/e] calc. for C_14_H_19_N_6_O_2_ [M + H]^+^ 303.1564, found 303.1556 (see Figure S8 in the Supplementary Material); ATR-FTIR: ν_max_ (cm^−1^) 3388, 3301, 3146, 2963, 1663, 1616, 1538, 1188, 1034, 818 (see Figure S46 in the Supplementary Material); ^1^H-NMR (DMSO-d_6_, 300 MHz): δ (ppm) 0.90 (d, ^3^*J*_32_ = 6.6 Hz, 6H, two terminal methyl groups of the isobutyl chain), 2.30 (m, ^3^*J*_23_ ≈ 6.9 Hz, ^3^*J*_21_ ≈ 4.4 Hz 1H, isobutyl methanetriyl (methine) group, signal partially covered and disturbed by the isoxazole methyl group), 2.33 (s, 3H, methyl group of the isoxazole moiety), 2.52 (s, 3H), 3.98 (br apparent singlet, 2H, chain methylene connected with amine nitrogen at the 7th position), 7.33 (brs, 1H, -NH), 7.88 (brs, 2H, -NH_2_) (see Figure S44 in the Supplementary Material); ^13^C-NMR (DMSO-d_6_, 75.5 MHz): δ (ppm) 11.4, 19.7, 23.5, 26.1, 50.9, 81.6, 117.2, 153.7, 154.3, 154.7, 156.7, 157.2, 169.3 (see Figure S45 in the Supplementary Material).

##### *2-(5-Amino-3-Methylisoxazol-4-yl)-7-N-Pentylamino-5-Methyloxazolo[5,4-d]Pyrimidine* (**5e, SCM5**)

Method A (in ethanol)

To compound **4** (0.2 g, 0.58 mmol), a 30% solution of pentylamine in ethanol (1 mL) was added in one portion. The reaction mixture was cooled to 5–10 °C and stirred for 1 h. Then, the mixture was warmed to room temperature and maintained with stirring for 1.5 h. When the reaction was complete, as shown by TLC, the formed precipitate was filtered off, dried and crystallized from ethanol. The compound **5e** (0.104 g, 56.8%) was obtained as a white solid: m.p. 188–189 °C (dec.); HR-ESI-MS: *m/z*[u/e] calc. for C_15_H_21_N_6_O_2_ [M + H]^+^ 317.1720, found 317.1717 (see Figure S9 in the Supplementary Material); ATR-FTIR: ν_max_ (cm^−1^) 3407, 2959, 1664, 1616, 1592, 1521, 1350, 1178, 1043, 790 (see Figure S49 in the Supplementary Material); ^1^H-NMR (DMSO-d_6_, 300 MHz): δ (ppm) 0.89 (t, ^5^*J* ≈ 6.5 Hz, 3H, terminal methyl group of the pentyl chain), 1.30–1.35 (m, 4H, two methylene groups of pentyl chain), 1.66 (m, 2H, methylene group of the pentyl chain), 2.33 (s, 3H, methyl group of the isoxazole moiety), 2.53 (s, 3H), 4.08 (t, ^3^*J* ≈ 7.8 Hz, 2H, methylene group of the pentyl chain connected with amine nitrogen at position 7), 7.23 (brs, 1H, -NH), 7.86 (brs, 2H, -NH_2_) (see Figure S47 in the Supplementary Material); ^13^C-NMR (DMSO-d_6_, 75.5 MHz): δ (ppm) 11.4, 13.8, 21.8, 22.9, 26.2, 28.5, 44.7, 81.6, 117.0, 153.7, 153.8, 154.8, 156.3, 157.2, 169.3 (see Figure S48 in the Supplementary Material).

##### *2-(5-Amino-3-Methylisoxazol-4-yl)-7-N-(2-Hydroxyethyl)Amino-5-Methyloxazolo[5,4-d]Pyrimidine* (**5f, SCM6**)

Method A (in ethanol)

To compound **4** (0.2 g, 0.58 mmol), a 30% solution of ethanolamine in ethanol (1 mL) was added in one portion. The reaction mixture was cooled to 5–10 °C and stirred for 1 h. Then, the mixture was warmed to room temperature and maintained with stirring for 1.5 h. When the reaction was complete, as shown by TLC, the formed precipitate was filtered off, dried and crystallized from ethanol. The compound **5f** (0.083 g, 49.4%) was obtained as a white solid: m.p. 251–252 °C (dec.); HR-ESI-MS: *m/z*[u/e] calc. for C_12_H_15_N_6_O_3_ [M + H]^+^ 291.1200, found 291.1195 (see Figure S10 in the Supplementary Material); ATR-FTIR: ν_max_ (cm^−1^) 3324, 3259, 1662, 1622, 1592, 1531, 1442, 1352, 1270, 1166, 1050 (see Figure S52 in the Supplementary Material); ^1^H-NMR (DMSO-d_6_, 300 MHz): δ (ppm) 2.34 (s, 3H, methyl group of the isoxazole moiety), 2.59 (s, 3H, methyl group at position 5 of the oxazolopyrimidine system), 3.72 (t, ^3^*J* = 5.2 Hz, 2H, methylene group connected with the amine nitrogen at position 7), 4.20 (t, ^3^*J* = 5.4 Hz, 2H, methylene group connected hydroxyl group), 5.03 (brs, 1H, -OH), 7.23 (brs, 1H, -NH), 7.87 (brs, 2H, -NH_2_) (see Figure S50 in the Supplementary Material); ^13^C-NMR (DMSO-d_6_, 75.5 MHz): δ (ppm) 11.2, 23.5, 47.3, 57.7, 81.6, 117.0, 153.5, 154.0, 154.8, 157.1, 157.2, 169.2 (see Figure S51 in the Supplementary Material).

##### *2-(5-Amino-3-Methylisoxazol-4-yl)-7-N-(3-Hydroxypropyl)Amino-5-Methyloxazolo[5,4-d]Pyrimidine* (**5g, SCM7**)

Method A (in ethanol)

To compound **4** (0.2 g, 0.58 mmol), a 30% solution of 3-aminopropan-1-ol in ethanol (1 mL) was added in one portion. The reaction mixture was cooled to 5–10 °C and stirred for 1 h. Then, the mixture was warmed to room temperature and maintained with stirring for 1.5 h. When the reaction was complete, as shown by TLC, the formed precipitate was filtered off, dried and crystallized from ethanol. The compound **5g** (0.103 g, 58.5%) was obtained as a white solid: m.p. 224–226 °C (dec.); HR-ESI-MS: *m/z*[u/e] calc. for C_13_H_17_N_6_O_3_ [M + H]^+^ 305.1357, found 305.1352 (see Figure S11 in the Supplementary Material); ATR-FTIR: ν_max_ (cm^−1^) 3243, 3140, 1663, 1616, 1594, 1538, 1351, 1168, 1086, 1050, 795 (see Figure S55 in the Supplementary Material); ^1^H-NMR (DMSO-d_6_, 300 MHz): δ (ppm) 1.84 (m, ^3^*J* ≈ 6.3–6.5 Hz, 2H, middle methylene group of the propyl chain), 2.34 (s, 3H, methyl group of the isoxazole moiety), 2.56 (s, 3H, methyl group at position 5 of the oxazolopyrimidine system), 3.44 (heavily distorted triplet t, ^3^*J* is difficult to estimate, 2H, methylene group connected with amine nitrogen at position 7 of the oxazolopyrimidine system), 4.19 (t, ^3^*J* = 6.9 Hz, 2H, methylene group connected hydroxyl group), 4.98 (brs, 1H, -OH), 7.24 (brs, 1H, -NH), 7.88 (brs, 2H, -NH_2_) (see Figure S53 in the Supplementary Material); ^13^C-NMR (DMSO-d_6_, 75.5 MHz): δ (ppm) 11.4, 22.9, 30.2, 42.4, 57.7, 81.6, 117.0, 153.8, 154.3, 155.1, 156.6, 157.2, 169.4 (see Figure S54 in the Supplementary Material).

##### *2-(5-Amino-3-Methylisoxazol-4-yl)-7-N-[2-(Morpholin-4-yl)Ethyl]amino-5-Methyloxazolo[5,4-d]Pyrimidine* (**5h, SCM8**)

Method A (in ethanol)

To compound **4** (0.2 g, 0.58 mmol), a 30% solution of 2-(morpholin-4-yl)ethanamine in ethanol (1 mL) was added in one portion. The reaction mixture was cooled to 5–10 °C and stirred for 1 h. Then, the mixture was warmed to room temperature and maintained with stirring for 3.5 h. When the reaction was complete, as shown by TLC, the formed precipitate was filtered off, dried and crystallized from ethanol. The compound **5h** (0.087 g, 41.8%) was obtained as a white solid: m.p. 207–209.5 °C (dec.); HR-ESI-MS: *m/z*[u/e] calc. for C_16_H_22_N_7_O_3_ [M + H]^+^ 360.1779, found 360.1768 (see Figure S12 in the Supplementary Material); ATR-FTIR: ν_max_ (cm^−1^) 3332, 2960, 2801, 1661, 1615, 1593, 1532, 1352, 1282, 1166, 1115, 1054, 1009, 868, 782 (see Figure S58 in the Supplementary Material); ^1^H-NMR (DMSO-d_6_, 300 MHz): δ (ppm) 2.33 (s, 3H, methyl group of the isoxazole moiety), 2.46 (t, ^3^*J* ≈ 4.5 Hz, 4H, two morpholine ring methylene group connected directly with the morpholine ring nitrogen), 2.59 (s, 3H, methyl group at position 5 of the oxazolopyrimidine system), 2.61 (t, ^3^*J* ≈ 6.8 Hz, 2H, methylene group of ethyl chain directly connected with the morpholine ring nitrogen), 3.56 (t, ^3^*J ≈* 4.5 Hz, 4H, two morpholine ring methylene group connected directly with the morpholine ring oxygen), 4.23 (t, ^3^*J* ≈ 6.5 Hz, 2H, methylene group of the ethyl chain directly connected with the amine nitrogen at position 7 of the oxazolopyrimidine system), 7.26 (brs, 1H, -NH), 7.87 (brs, 2H, -NH_2_) (see Figure S56 in the Supplementary Material); ^13^C-NMR (DMSO-d_6_, 75.5 MHz): δ (ppm) 11.4 (carbon of the methyl group of the isoxazole moiety), 23.2 (carbon of the methyl group at position 5 of the oxazolopyrimidine system), 42.5 (carbon of the ethyl chain methylene group directly connected with the amine nitrogen at position 7 of the oxazolopyrimidine system), 53.6 (carbons of two morpholine ring methylene group connected directly with the morpholine ring nitrogen), 54.9 (carbon of the ethyl chain methylene group directly connected with the morpholine ring nitrogen), 66.2 (carbons of two morpholine ring methylene group connected directly with the morpholine ring oxygen), 81.6 (quaternary carbon C4 of the isoxazole ring), 117.0 (quaternary bridgehead carbon C7a of the oxazolopyrimidine system), 153.7 (quaternary bridgehead carbon C3a of the oxazolopyrimidine system, uncertain assignment), 153.8 (quaternary carbon C2 of the oxazolopyrimidine system), 154.8 (quaternary carbon C7 of the oxazolopyrimidine system, uncertain assignment), 156.8 (quaternary carbon C5 of the oxazolopyrimidine system), 157.2 (quaternary carbon C3 of the isoxazole ring), 169.3 (quaternary carbon C5 of the isoxazole ring) (see Figure S57 in the Supplementary Material).

##### *2-(5-Amino-3-Methylisoxazol-4-yl)-7-N-[3-(N,N-Dimethylamino)Propyl]Amino-5-Methyloxazolo[5,4-d]Pyrimidine* (**5i, SCM9**)

Method A (in ethanol)

To compound **4** (0.2 g, 0.58 mmol), a 30% solution of *N*,*N*-dimethylpropane-1,3-diamine in ethanol (1 mL) was added in one portion. The reaction mixture was cooled to 5–10 °C and stirred for 1 h. Then, the mixture was warmed to room temperature and maintained with stirring for 4 h. When the reaction was complete, as shown by TLC, the formed precipitate was filtered off, dried and crystallized from ethanol. The compound **5i** (0.127 g, 66.1%) was obtained as a white solid: m.p. 209.5–210 °C (dec.); HR-ESI-MS: *m/z*[u/e] calc. for C_15_H_22_N_7_O_2_ [M + H]^+^ 332.1829, found 332.1821 (see Figure S13 in the Supplementary Material); ATR-FTIR: ν_max_ (cm^−1^) 3469, 3364, 2774, 1674, 1615, 1595, 1529, 1440, 1346, 1183, 1041 (see Figure S61 in the Supplementary Material); ^1^H-NMR (DMSO-d_6_, 300 MHz): δ (ppm) 1.82 (m, ^3^*J_23_* = 6.6 Hz, ^3^*J_21_* = 7.6 Hz, 2H, middle methylene group of the propyl chain), 2.14 (s, 6H, two methyls of the dimethylamino group), 2.28 (t, ^3^*J_32_* = 6.6 Hz, 2H, methylene of the propyl chain directly connected to nitrogen of the dimethylamino group), 2.33 (s, 3H, methyl group of the isoxazole moiety), 2.55 (s, 3H, methyl group at position 5 of the oxazolopyrimidine system), 4.11 (t, ^3^*J_12_* = 7.5 Hz, 2H, methylene of the propyl chain directly connected to the amine nitrogen at position 7 of the oxazolopyrimidine system), 7.23 (brs, 1H, -NH), 7.86 (brs, 2H, -NH_2_) (see Figure S59 in the Supplementary Material); ^13^C-NMR (DMSO-d_6_, 75.5 MHz): δ (ppm) 11.4, 22.9, 24.4, 43.5, 45.1, 56.3, 81.6, 117.1, 153.6, 153.8, 154.8, 156.4, 157.2, 169.3 (see Figure S60 in the Supplementary Material).

Method B (in tetrachloromethane)

To compound **4** (0.2 g, 0.58 mmol), 30% solution of *N*,*N*-dimethylpropane-1,3-diamine in CCl_4_ (1 mL) was added in one portion. The reaction mixture was cooled to 5–10 °C and stirred for 1 h. Then, the mixture was warmed to room temperature and maintained with stirring for 47 h. When the reaction was complete, as shown by TLC, the reaction mixture was evaporated in a vacuum and purified by chromatography on silica gel (CHCl_3_: AcOEt, 9:1). The compound **5i** (0.06 g, 30.6%) was obtained as a beige solid: m.p. 202–204 °C (dec.); HR-ESI-MS: *m/z* [M + H]^+^ calc. for C_15_H_22_N_7_O_2_ 332.1829, found 332.1824 (see Figure S14 in the Supplementary Material). Other spectral analyses (IR, NMR) confirmed that the product is identical to that obtained by Method A.

Method C

To compound **3** (0.05 g, 0.18 mmol), a 30% solution of *N*,*N*-dimethylpropane-1,3-diamine in ethanol (0.25 mL) was added in one portion. The reaction mixture was cooled to 5–10 °C and stirred for 1 h. Then, the mixture was warmed to room temperature and maintained with stirring for 4 h. When the reaction was complete, as shown by TLC, the formed precipitate was filtered off, dried and crystallized from ethanol. The compound **5i** (0.007 g, 11.6%) was obtained as a white solid: m.p. 210–211 °C (dec.); ESI-MS: *m/z*[u/e] calc. for C_15_H_22_N_7_O_2_ [M + H]^+^ 332.1829, found 332.1848 (see Figure S15 in the Supplementary Material). Other spectral analyses (IR, NMR) confirmed that the product is identical to that obtained by Methods A and B.

##### *2-(5-Amino-3-Methylisoxazol-4-yl)-7-N-[2-(N,N-Dimethylamino)Ethyl]amino-5-Methyloxazolo[5,4-d]Pyrimidine* (**5j, SCM10**)

Method A (in ethanol)

To compound **4** (0.2 g, 0.58 mmol), a 30% solution of *N*,*N*-dimethylethane-1,2-diamine in ethanol (1 mL) was added in one portion. The reaction mixture was cooled to 5–10 °C and stirred for 1 h. Then, the mixture was warmed to room temperature and maintained with stirring for 2 h. When the reaction was complete, as shown by TLC, the formed precipitate was filtered off, dried and crystallized from ethanol. The compound **5j** (0.078 g, 42.4%) was obtained as a white solid: m.p. 222.5–223 °C (dec.); HR-ESI-MS: *m/z*[u/e] calc. for C_14_H_20_N_7_O_2_ [M + H]^+^ 318.1673, found 318.1664 (see Figure S16 in the Supplementary Material); ATR-FTIR: ν_max_ (cm^−1^) 3226, 2832, 2780, 1665, 1616, 1593, 1529, 1450, 1352, 1184, 1027 (see Figure S69 in the Supplementary Material); ^1^H-NMR (DMSO-d_6_, 300 MHz): δ (ppm) 2.22 (s, 6H, dimethylamino group), 2.33 (s, 3H, methyl group of the isoxazole moiety), 2.54 (heavily distorted and partly covered with solvent triplet t, ^3^*J_21_* is difficult to estimate, 2H, methylene of the propyl chain directly connected to the nitrogen of the dimethylamino group) 2.58 (s, 3H, methyl group at position 5 of the oxazolopyrimidine system), 4.20 (t, ^3^*J_12_* = 6.7 Hz, 2H, methylene of the ethyl chain directly connected to the amine nitrogen at position 7 of the oxazolopyrimidine system), 7.25 (brs, 1H, -NH), 7.87 (brs, 2H, -NH_2_) (see Figure S67 in the Supplementary Material); ^13^C-NMR (DMSO-d_6_, 75.5 MHz): δ (ppm) 11.4, 23.1, 43.1, 45.5, 55.8, 81.6, 117.0, 153.6, 153.8, 154.7, 156.8, 157.2, 169.3 (see Figure S68 in the Supplementary Material).

##### *2-(5-Amino-3-Methylisoxazol-4-yl)-7-N-Ispropylamino-5-Methyloxazolo[5,4-d]Pyrimidine* (**5k**)

Method A (in ethanol)

To compound **4** (0.29 g, 0.84 mmol), a 30% solution of isopropylamine in ethanol (1 mL) was added in one portion. The reaction mixture was cooled to 5–10 °C and stirred for 1 h. Then, the mixture was warmed to room temperature and maintained with stirring for 3.5 h. When the reaction was complete, as shown by TLC, the formed precipitate was filtered off. The solution after filtration was evaporated in a vacuum and crystallized from ethanol. The compound **5k** (0.022 g, 9.1%) was obtained as a beige solid: m.p. 161–162 °C (dec.); HR-ESI-MS: *m/z*[u/e] calc. for C_13_H_17_N_6_O_2_ [M + H]^+^ 289.1408, found 289.1396 (see Figure S17 in the Supplementary Material); ATR-FTIR: ν_max_ (cm^−1^) 3085, 2925, 1633, 1604, 1315, 1042, 744 (see Figure S72 in the Supplementary Material); ^1^H-NMR (DMSO-d_6_, 300 MHz): δ (ppm) 1.62 (d, ^3^*J* = 6.7 Hz, 6H, two methyl groups of the isopropyl chain), 2.32 (s, 3H, methyl group of the isoxazole moiety), 2.54 (s, 3H, methyl group at position 5 of the oxazolopyrimidine system), 4.69 (br m, very low intensity, 1H, methanetriyl (methine) group of the isopropyl chain), 7.32 (brs, 1H, -NH), 7.85 (brs, 2H, -NH_2_) (see Figure S70 in the Supplementary Material); ^13^C-NMR δ (ppm) 11.4 (carbon of methyl group of the isoxazole moiety), 18.5 (carbons of the two methyl groups of the isopropyl moiety), 24.3 (carbon of the methyl group at position 5 of the oxazolopyrimidine system), 53.4 (methanetriyl (methine) group of the isopropyl chain), 81.6 (C4 of the isoxazole ring), 118.1 (quaternary bridgehead carbon C7a of the oxazolopyrimidine system), 153.6, 154.1, 154.5 (three quaternary carbons C2, C3a and C7 of the oxazolopyrimidine system), 156.6 (quaternary carbon C5 of the oxazolopyrimidine system), 157.2 (quaternary carbon C3 of the isoxazole ring), 169.3 (quaternary carbon C5 of the isoxazole ring) (see Figure S71 in the Supplementary Material).

##### *2-(5-Amino-3-Methylisoxazol-4-yl)-7-N-Cyclohexylamino-5-Methyloxazolo[5,4-d]Pyrimidine* (**5l**)

Method A (in ethanol)

To compound **4** (0.3 g, 0.87 mmol), a 30% solution of cyclohexylamine in ethanol (1 mL) was added in one portion. The reaction mixture was cooled to 5–10 °C and stirred for 1 h. Then, the mixture was warmed to room temperature and maintained with stirring for 24 h. When the reaction was complete, as shown by TLC, the formed precipitate was filtered off and washed with chloroform. CHCl_3_-insoluble solid was filtered off. The filtrate was evaporated in a vacuum and purified by chromatography on silica gel (CHCl_3_:MeOH, 9:1). Compound **5l** (0.064 g, 22.4%) was obtained as a light beige solid: m.p. 206-208 °C (dec.); HR-ESI-MS: *m/z*[u/e] calc. for C_16_H_21_N_6_O_2_ [M + H]^+^ 329.1720, found 329.1706 (see Figure S19 in the Supplementary Material); ATR-FTIR: ν_max_ (cm^−1^) 3299, 3122, 2936, 1675, 1646, 1533, 1431, 1317, 1164, 1046 (see Figure S75 in the Supplementary Material); ^1^H-NMR (CDCl_3_, 300 MHz): δ (ppm) 1.21–1.82 (two multiplets, 8H, methylene protons of the cyclohexyl ring), 2.32 (s, 3H, methyl group of the isoxazole moiety), 2.62 (s, 3H, methyl group of the acetamidine moiety), 2.98 (brs, 2H, -CH_2_-, methylene protons connected to C4 of the cyclohexyl ring), 4.22 (br m, low intensity, 1H, methanetriyl (methine) group of the cyclohexyl ring), 7.80 (low intensity, 1H, -NH), 7.96 (brs, 2H, -NH_2_) (see Figure S73 in the Supplementary Material); ^13^C-NMR (DMSO-d_6_, 75.5 MHz): δ (ppm) 11.4 (carbon of methyl group of the isoxazole moiety), 24.5 (carbon of methyl group at position 5 of the oxazolopyrimidine system), 25.9, 26.5, 29.0 (carbons of cyclohexyl methylene groups -CH_2_-), 62.1 (methanetriyl (methine) carbon (-CH-N) of the *N*-cyclohexyl group at position 7 of the oxazolopyrimidine system), 81.5 (quaternary carbon C4 of the isoxazole ring), 118.3 (quaternary bridgehead carbon C7a of the oxazolopyrimidine system), 154.0, 154.3, 154.9 (three quaternary carbons C2, C3a and C7 of the oxazolopyrimidine system), 156.8 (quaternary carbon C5 of the oxazolopyrimidine system), 157.2 (quaternary carbon C3 of the isoxazole ring), 169.6 (quaternary carbon C5 of the isoxazole ring) (see Figure S74 in the Supplementary Material).

##### *N’-[2-(5-Amino-3-Methylisoxazol-4-yl)-4-Cyanooxazol-5-yl]-N,N-Diethylacetamidine* (**7m**)

To compound **4** (0.15 g, 0.43 mmol), a 50% solution of *N*,*N*-diethylamine in ethanol (1 mL) was added in one portion. The reaction mixture was cooled to 5–10 °C and stirred for 1 h. Then, the mixture was warmed to room temperature and maintained with stirring for 4 days. When the reaction was complete, as shown by TLC, the reaction mixture was evaporated and purified by chromatography on silica gel (CHCl_3_:EtOAc, 1:1). Compound **7m** (0.019 g, 14.5%) was obtained as a white solid: m.p. 98–99 °C (dec.); HR-ESI-MS: *m/z*[u/e] calc. for C_28_H_36_N_12_O_4_Na [2M + Na]^+^ 627.2875, found 627.2885 (see Figure S24 in the Supplementary Material); ATR-FTIR: ν_max_ (cm^−1^) 3409, 3118, 2976, 2934, 2215 (CN), 1632, 1600, 1559, 1429, 1359, 1291, 1026 (see Figure S78 in the Supplementary Material); ^1^H-NMR (CDCl_3_, 300 MHz): δ (ppm) 1.23 (t, *J* = 7.1Hz, 3H, CH_3_ connected with CH_2_ at 3.62 ppm), 1.25 (t, *J* = 7.2Hz, 3H, CH_3_ connected with CH_2_ at 3.42 ppm), 2.26 (s, 3H. methyl group of the acetamidine moiety, located in space closer to *N*-ethyl group that is characterized with following chemical shifts 1.25 (-CH_3_) and 3.65 (-CH_2_-) ppm), 2.41 (s, 3H, methyl group of the isoxazole moiety), 3.42 (q, *J* = 7.1Hz, 2H, -CH_2_-), 3.65 (q, *J* = 7.0Hz, 2H, -CH_2_-), 5.96 (brs, 2H, NH_2_) (see Figure S76 in the Supplementary Material); ^13^C-NMR (CDCl_3_): δ (ppm) 11.7 (CH_3_, isoxazole), 12.4 (CH_3_CH_2_N, methyl carbon connected with the methylene carbon at 43.24 ppm), 14.0 (CH_3_CH_2_N, methyl carbon connected with the methylene carbon at 43.97 ppm), 16.7 (CH_3,_ acetamidine), 43.1 (N-CH_2_CH_3_), 43.8 (N-CH_2_CH_3_), 83.8 (quaternary carbon C4 of the isoxazole ring), 94.6 (quaternary carbon C4 of the oxazole ring), 115.8 (CN), 150.3 (quaternary carbon C2 of the oxazole ring), 157.7 (quaternary carbon C5 of the oxazole ring), 160.3 (quaternary carbon C3 of the isoxazole ring), 161.2 (quaternary carbon of the acetamidine moiety), 168.1 (quaternary carbon C5 of the isoxazole ring) (see Figure S77 in the Supplementary Material).

#### 3.1.3. Single Crystal X-ray Diffraction Measurement of the Compound **5h**

The single crystals of the oxazolo[5,4-*d*]pyrimidine derivative **5h** suitable for X-ray diffraction analysis were obtained as follows: a small amount of the compound **5h** was dissolved in methanol and the solution was stirred for about 10 min at 40 °C to ensure homogeneity and then cooled. After dissolving, the solution sample was sealed with a perforated film and placed under ambient conditions for slow evaporation. During the recrystallization, 3–4 drops of 2-propanol and methanol were added several times. Colorless needle-shaped crystals, suitable for X-ray diffraction experiments, were obtained after several weeks.

The X-ray diffraction data for **5h** were obtained with an Xcalibur automated four-circle diffractometer with Ruby CCD camera using Enhance Source Cu-Kα radiation (λ = 1.5418 Å). The data were collected at 200(2) K by using an Oxford-Cryosystems cooler. Data collection, cell refinement, data reduction and analysis were carried out with CrysAlisPro [43]. Diffraction data have been corrected for absorption effects by multiple scans [43]. The crystal structure was solved using SHELXS [44]. Refinements were performed using SHELXL-2014/7 [45] based on F^2^ through the full-matrix least-squares method. All H atoms were found in difference Fourier maps, and in the final refinement cycle they were repositioned in their calculated positions and refined using a riding model, with the length of C–H = 0.98–0.99 Å and with U_iso_(H) = 1.2 U_eq_(C) for CH_2_ and 1.5 U_eq_(C) for CH_3_; and N-H = 0.88 Å and with U_iso_(H) = 1.2 U_eq_(N) for NH and NH_2_. All non-hydrogen atoms were refined with anisotropic atomic displacement parameters. The structure plots for **5h** were prepared with DIAMOND [46].

CCDC No. 2,005,200 contains the supplementary crystallographic data for this paper. These data can be obtained free of charge via www.ccdc.cam.ac.uk, or from the Cambridge Crystallographic Data Centre, 12 Union Road, Cambridge CB2 1EZ, UK; fax: (+44) 1223-336-033; or e-mail: deposit@ccdc.cam.ac.uk.

Crystal data for **5h**

C_16_H_21_N_7_O_3_, M = 359.40, colorless needle, crystal dimensions: 0.14 × 0.02 × 0.01 mm; triclinic, space group *P* $\bar{1}$; *a* = 8.524 (2) Å, *b* = 10.246 (3) Å, *c* = 10.775 (3) Å, *α* = 83.65 (5)°, *β* = 89.60 (4)°, *γ* = 69.34 (5)°; V = 874.6 (5) Å^3^; T = 200(2) K; Z = 2; *ρ*_calc_ = 1.365 g cm^−3^; *μ* = 0.82 mm^−1^ (for Cu-Kα, λ = 1.54184 Å); F(000) = 380; reflections collected = 6213; reflections independent = 3137 [Rint = 0.027]; reflections observed = 2549 [I > 2σ(I)]; θ range 4.1–67.8°; *h*, *k*, *l* range: −7 ≤ h ≤ 10, −12 ≤ l ≤ 11, −12 ≤ k ≤ 12; parameters = 237; restraints = 0; *R_1_* = 0.039; wR_2_ = 0.103 [*F*^2^ > 2σ(*F*^2^)]; GooF = S = 1.02; largest difference in peak and hole, Δ*ρ*_max_ and Δ*ρ*_min_ = 0.27 and −0.18 e Å^−3^; absorption corrections: empirical correction (multi scan), transmission factors: T_min_ = 0.924, T_max_ = 1.000.

#### 3.1.4. Computational Details

A full geometry optimization and the thermochemistry of the following compounds has been performed on the basis of ab initio quantum mechanical DFT (density functional theory) method using the B3LYP [47,48] hybrid density functional with the extended 6-311++G(*df,pd*) basis set:

(a) **5a**, **5c** and their respective theoretical isoforms **8a**, **8c**;
(b) **5h** and its imine tautomer;
(c) *N′*-Cyanooxazolylacetamidine **7m** and its corresponding theoretical structurally isomeric 7-*N*,*N*-diethyloxazolo[5,4-*d*]pyrimidine;
(d) Theoretical *N′*-cyanooxazolylacetamidine **7** (where R = Me, R^1^ = H) (see Scheme 1)

The molecules of the compounds **5a**, **5c**, **8a, 8c**, **5h**, **7m** and their theoretical isomers (tautomers) were treated as single isolated molecules in vacuum environment. Additionally, in the case of the compounds **5a, 5c**, **8a, 8c**, **5h** and its imine tautomer, the Conductor-like Polarizable Continuum Model (CPCM) [32] was used for simulation of ethanol environment with Gaussian 16 default set of parameters (Scaling factor for van der Waals radius for all atoms, i.e., alpha = 1.1). All the calculations were computed using the Gaussian 2016 revision C.01 software [49]. To possibly get most accurate approximation the following keywords, i.e., Fopt = (Tight,CalcAll), SCF = (Direct,VeryTight) and Integral(Grid = SuperFineGrid) were used in the calculations. The confirmation that the final structure and presented results correspond to a minimum on the potential energy surface is the absence of imaginary wavenumbers in the results of frequency calculations. The thermochemistry calculations were carried out for standard conditions of temperature and pressure (i.e., T = 298.15 K and p = 1 atm). Stabilization energy (presented here as E_stab_ = −ΔE = 13.06 kcal mol^−1^ for vacuum conditions) of the compound **5a** relative to its isomeric **8a** was calculated as the difference (ΔE_298_ = −13.06 kcal·mol^−1^) between thermal-corrected energies (E = E_elec._ + ZPE + E_trans._ + E_rot._ + E_vib._) of the compound **5a** and its isoform **8a**. Taking into account the ethanol environment, the appropriate values of E_stab_ are 12.43 kcal·mol^−1^ for the compounds (**5a** versus **8a**). For the compound **5c** and its isomeric **8c**, ΔE_298_ = −13.23 kcal·mol^−1^, which gives E_stab_ = 13.23 kcal·mol^−1^ for vacuum and ΔE_298_ = −12.83 kcal·mol^−1^, which gives E_stab_ = 12.83 kcal·mol^−1^ for ethanol environment.

Free enthalpy (ΔG_trans(298)_ = 10.748 kcal·mol^−1^) of transition of proton between amine-imine tautomers of the compound **5h** was calculated as difference between free enthalpy (Δ_f_G_imine_) of formation of its imine tautomer and free enthalpy (Δ_f_G_amine_) of formation of its amine tautomer. Because an equilibrium constant K is related to the standard free enthalpy change ΔG^°^ through the following expression ΔG^°^ = −RT·lnK, where R is molar gas constant (1.98720425864083·10^−3^ kcal·K^−1^·mol^−1^) and T is absolute temperature (298.15 K), thus, using this relationship, the equilibrium constant of amine-imine transition K_trans(298)_, which express a ratio of moles (or molar concentration) of imine tautomer to moles (or molar concentration) of amine one, can be easily estimated from ΔG_trans(298)_ = −RT·ln K_trans_, it follows that K_trans(298)_ ≈ 1.323 × 10^−8^ which can be expressed that one molecule of less stable imine tautomer of the compound **5h** falls to (occurs with) over 75,500,000 (75.5 million) molecules of more stable amine tautomer of the compound **5h** in the equilibrium. Taking into account the ethanol environment, the appropriate values are ΔG_trans(298)_ = 10.752 kcal·mol^−1^ and K_trans(298)_ ≈ 1.313 × 10^−8^ which can be expressed that one molecule of less stable imine tautomer of the compound **5h** falls to (occurs with) over 7,600,000 (76 million) molecules of more stable amine tautomer of the compound **5h** in the equilibrium.

Theoretically estimated enthalpies of formation (Δ_f_H_298_) at 298.15 K in gas phase for *N′*-cyanooxazolylacetamidine **7m** and its corresponding theoretical 7-*N*,*N*-diethylaminooxazolo[5,4-*d*]pyrimidine isomer are 62.2 kcal·mol^−1^ and 38.7 kcal·mol^−1^ respectively. For compound **5a** (where R = Me) and its corresponding theoretical *N′*-cyanooxazolylacetamidine **7** (where R = Me, R^1^ = H), appropriate values of enthalpies of formation (Δ_f_H_298_) are as following 38.4 kcal mol^−1^ and 63.4 kcal mol^−1^, respectively.

Potential Energy Surface (PES) was scanned through the change of dihedral angle **C**H_3_-**C**-**N**-**C**H_2_CH_3_ of acetamidine moiety of *N′*-cyanooxazolylacetamidine **7m** in the full range of −180–180° as a result of rotation about C-N bond to find highest energy conformers, which were needed to estimate the rotational energy barrier. As a result, two maxima of energy were found, which are connected with two conformers, for which appropriate dihedral angle **C**H_3_-**C**-**N**-**C**H_2_CH_3_ was as follows −80.839° and 105.162°, respectively. Predicted energy (E_rot_ = 23.4 kcal·mol^−1^) needed to overcome a rotational barrier to internal free rotation around bond CH_3_C—N(Et)_2_ of acetamidine moiety at fifth position of oxazole ring of *N′*-cyanooxazolylacetamidine **7m** has been calculated as difference between free enthalpy of most energetically unstable (where appropriate dihedral angle **C**H_3_-**C**-**N**-**C**H_2_CH_3_ is −80.839°) and most energetically stable conformer (where appropriate dihedral angle is −3.838°). Details on how to calculate enthalpies and Gibbs free energy of formation with using Gaussian can be found in the paper “Thermochemistry in Gaussian” [50].

### 3.2. Experimental Biological Section

#### 3.2.1. General Information Concerning Preparation for Tests and Statistics

##### Cell Lines and Reagents

WEHI-231—a murine B cell line (ATCC, CRL-1702), Jurkat—a human T cell line (ATCC, TIB-152), A-549—a human adenocarcinoma lung cells (ATCC CCL185), L-1210 (ATCC^®^ CCL-219™)—a mouse leukemia, HT-29 (ATCC^®^ HTB-38™)—a human colon tumor and A-431 (ATCC^®^ CRL-1555™)—a human epidermal cell lines derived from the Collection of Cell Lines of The Institute of Immunology and Experimental Therapy (Wrocław, Poland). Dulbecco′s Modified Eagle’s Medium (DMEM) was obtained from the American Type Culture Collection—ATCC (Manassas, VA, USA), RPMI-1640 and Hanks’ medium were purchased from Biowest (Nuaillé, France). Fetal calf serum (FCS) was from HyClone (Pittsburgh, PA, USA). Other reagents such as L-glutamine, penicillin and streptomycin solution, 2-mercaptoethanol, lipopolysaccharide (LPS) from *Escherichia coli* strain O111:B4 (L4130), teriflunomide (SML0936) and MTT (3-[4,5-dimethylthiazol-2-yl]-2,5-diphenyltetrazoliumbromide) were purchased from Sigma-Aldrich (St. Louis, MO, USA). Human herpes virus type-1 (HHV-1), McIntyre strain, *Herpesviridae*,** DNA enveloped virus was from the Laboratory of Virology, Institute of Immunology and Experimental Therapy. TNF α levels in the supernatants were determined by ELISA kit from eBioscience (cat. 88-7346-88, Thermo Fisher Scientific Inc., Waltham, MA, USA).

##### Mice

Two month-old, specific pathogen free BALB/c male mice were provided by the Center of Experimental Medicine, Medical University (Białystok, Poland), The mice were kept in 12/12 h light/dark cycles with a free access to filtered tap water and commercial food. The animals were used only as organ donors what does not require approval of ethics committee according to Directive 2010/63/EU of the European Union Parliament and of the Council of 22 September 2010 on the protection of animals used for scientific purposes.

##### Preparation of the Oxazolo[5,4-*d*]Pyrimidine SCM Derivatives for In Vitro Experiments

The compounds **SCM1**–**10** were initially dissolved in DMSO (6.25 mM). Then, the solutions were further diluted in the culture medium. In parallel, respective DMSO solutions were also prepared.

##### Statistics

The results were subjected to statistical analysis using analysis of variance (one-way ANOVA) in STATISTICA 7 for Windows (TIBCO Software Inc., Palo Alto, CA, USA). Brown-Forsyth′s test was used to determine the homogeneity of variance between groups. When the variance was homogenous, analysis of variance (one-way ANOVA) was applied, followed by post hoc comparisons with the Tukey’s test to estimate the significance of the difference between groups. Nonparametric data were evaluated with the Kruskal-Wallis analysis of variance. Significance was determined at *p* < 0.05. The results are presented as mean values ±SE. Two/three independent assays were conducted and the results are presented as mean values from quadruplicate determinations ±SE, taking into account only one representative assay.

#### 3.2.2. Biological Tests

##### Determination of the Toxicity of the SCM Compounds against the A-549 Cell Line

The cytotoxicity of the compounds was determined by measuring growth of human lung epithelial A-549 cells (Criteria of toxicity effect see Table 8). The cells were suspended in a density of 2 × 10^5^/mL in a culture medium, consisting of RPMI-1640 with the addition of 2% FCS, 100 U/mL of penicillin, 100 μg/mL streptomycin, and 2 mM L-glutamine (referred to below as “the culture medium”). The compounds were tested at a concentration range of 62.5–15.6 μM. The test was performed in 96-flat bottom plates containing 2 × 10^4^/100 µL /well cells incubated initially for 24 h in a cell culture incubator. Then, the supernatants were removed and appropriate dilutions of the compounds (0.2 mL) were added to the cells in a cell culture medium. The cultures were incubated for 72 h followed by determination of cell viability by a colorimetric MTT method measuring optical density (OD). In parallel, control cultures containing appropriate dilutions of the solvent (DMSO) were also incubated. The results are given in percentage inhibition in relation with appropriate DMSO controls (see Table 1). Data were shown as mean ±SE.

##### Isolation of the Peripheral Blood Mononuclear Cells (PBMCs)

Venous blood from a single donor was withdrawn into heparinized syringes and diluted twice with phosphate buffered saline (PBS). PBMCs were isolated by centrifugation on a Ficoll-Uropoline gradient (density 1.077 g/mL) at 400× *g* for 20 min at 4 °C. The interphase cells were then washed three times with Hanks’ medium and re-suspended in the culture medium at a density of 2 × 10^6^ cells/mL.

##### PHA-Induced Proliferation of Human PBMC

PBMC were distributed into 96-well flat-bottom plates in 100 μL aliquots (2 × 10^5^ cells/well). PHA was added at a concentration of 5 μg/mL. The compounds were tested at the following concentrations of: 50, 25 and 12.5 μM. DMSO at appropriate dilutions served as the control. After three days of incubation in a cell culture incubator, the proliferative response of the cells was determined by colorimetric MTT [51]. The results were obtained as optical density (OD) at 550/630 nm values and presented as mean values from quadruplicate determinations ±SE.

##### LPS-Induced Proliferation of Mouse Splenocytes

The spleens were pressed against a plastic screen into 0.83% NH_4_Cl solution to lyze erythrocytes (5 min incubation at room temperature). The cells were then washed twice with Hanks’ medium, passed through a glass wool column to remove debris, and re-suspended in the culture medium. The cells were then distributed into 96-well flat-bottom tissue culture plates at a density of 2 × 10^5^/100 µL/well. 50 μg/mL of LPS was applied to induce cell proliferation. The compounds were added to the cultures at concentrations of: 25, 12.5, 6.25 and 3.125 μM. After a 3-day incubation, the cell proliferation was determined using a colorimetric MTT colorimetric assay. The results are presented as the mean OD (Optical density) at 550/630 nm from quadruplicate determinations ±SE.

##### LPS-Induced TNF-α Production in Whole Blood Cell Cultures

Human whole blood was diluted 10 times with RPMI-1640 medium and distributed to 24-well culture plates in 1 mL aliquots. The cultures were stimulated with LPS (100 ng/mL), and the studied compounds were added at concentrations of: 25, 10 and 1 μM. The control cultures contained DMSO in appropriate concentrations. After an overnight incubation, the supernatants were harvested and frozen at −80 °C until cytokine determination. TNF α levels in the supernatants were determined by ELISA kit and the results are shown as percentage of inhibition versus DMSO control (mean values ±SE).

##### Growth Inhibition of Tumor Cell Lines

The cells were re-suspended in the culture medium and distributed into 96-well flat-bottom plates. L-1210 was present at 1.5 × 10^4^ cells/well while HT-29 and A-431 at 2.5 × 10^4^ cells/well. The preparations were added to the wells at the concentration range of 50–12.5 μM. Cisplatin was used as a reference drug in the same concentrations. After 3-day incubation in a cell culture incubator, the proliferation was determined using MTT colorimetric method. The data are presented as a mean OD (Optical density) values from quadruplicate wells ±SE.

##### Antiviral Activity of the Compounds

In order to determine the antiviral activities, **SCM5** and **SCM9** compounds were tested using A-549 cells infected with HHV-1. Briefly, the A-549 cells were seeded in 96-well culture plates at a density of 1 × 10^5^ cells/mL and then incubated in 37 °C in a humidified atmosphere containing CO_2_ for 24h to produce a semi-confluent monolayer. Afterwards, A-549 cells were infected with HHV-1. After incubation for 1h in 37 °C/CO_2_ atmosphere to allow virus adsorption to the cells, the medium (virus inoculum) was removed and tested compounds or control solvent (DMSO) were added to the infected cells. Acyclovir was used as a reference drug. After 48 h incubation, the supernatants were collected and stored at −80 °C for virus titration using a standard Tissue Culture Infective Dose 50 (TCID_50_) method with a two-fold serial dilution. Briefly, the supernatants were diluted and adsorbed to A-549 cells at density 1 × 10^5^ cells/mL in 96-well plates. The plates were incubated in 37 °C/CO_2_ atmosphere. After 48 h of incubation viral cytopathic effect (CPE) was analyzed in an inverted microscope. The TCID50 was calculated by determining the end-point dilution of virus where 50% of the infected cells display CPE. The antiviral activity of tested compounds was determined by comparing the logarithmic reduction factor (log 10) of the viral titer with DMSO control. The antiviral activity virus was defined according to the requirements presented in PN-EN 14476:2013+A1:2015.

##### Colorimetric MTT Assay for Cell Growth and Kill

Briefly, 25 µL of MTT (5 mg/mL) stock solution was added per well at the end of cell incubation period and the plates were incubated for additional 3 h in a cell culture incubator. Then, 100 µL of the extraction buffer (20% SDS with 50% DMF, pH 4.7) was added. After an overnight incubation the optical density (OD) was measured at 550 nm with the reference wavelength of 630 nm in a Dynatech 5000 spectrophotometer (Dynatech Laboratories Inc., Alexandria, VA, USA).

##### Cultures of Jurkat and WEHI-231 Cells and Total RNA Isolation

WEHI-231 cells were cultured in medium consisting of DMEM supplemented with 10% FCS, l-glutamine, 2-mercaptoethanol and antibiotics, at a density of 10^6^ cells/mL. Jurkat cells were cultured in RMPI 1640 supplemented with 10% FCS, l-glutamine, and antibiotics, at a density of 10^5^ cells/mL. The cells cultures were exposed overnight to 10 μg/mL of the compounds. Then, the cells were centrifuged at 300× *g* for 10 min.

Total RNA isolation was performed with TRIzol Reagent (Ambion, Austin, TX, USA) accordingly to manufacturer’s recommendations. The cell pellet (2 × 10^6^ cells) was resuspended in 1 mL of TRIzol reagent, shaken, incubated for 10 min at room temperature (RT), supplemented with 0.2 mL of chloroform, shaken vigorously for 15 s, incubated for 3 min at RT and centrifuged at 12,000× *g* for 15 min at 4 °C. The water phase was collected, transferred to a new tube, supplemented with 0.5 mL of isopropanol, incubated at RT for 10 min and centrifuged at 12,000× *g* for 10 min at 4 °C. The RNA pellet was washed with 1 mL of 75% ethanol, dried in air and dissolved in 20–30 μL of sterile diethylpyrocarbonate-treated Mili-Q water. RNA samples were stored at −20 °C.

##### Reverse Transcription

Single stranded complementary DNA (cDNA) was synthesized with oligo (dT)12-18 primers from 5 μg of total RNA using a VerteKit (Novazym, Poznan, Poland) according to the manufacturer’s instruction.

##### Quantification of Gene Expression by Real Time PCR

Expression of genes for GAPDH, caspases 3, 7, 8 and 9, Bcl-2, Fas, NFκB1 and p53 was measured using AmpliQ 5×HOT EvaGreen^®^ qPCR Mix Plus (noROX) (Novazym) in “The CFX Connect Real-Time PCR Detection System” (BIORAD, Hercules, CA, USA) starting the reaction with 15 min of preincubation at 95 °C followed by 35 amplification cycles consisting of: denaturation in 95 °C for 15 s; annealing in 53 °C for 30 s and elongation in 72 °C for 15 s. GAPDH was used as a housekeeping gene for arbitrary unit calculation for every tested gene. The sequences of primers are enclosed in the Supplementary Material (Table S27).

## Figures and Tables

**Table 1:** The cytotoxic effect of **SCM** compounds on A-549 cell line. The cells were treated with different concentrations (62.5, 31.2 and 15.6 µM) of **SCM** compounds for 72 h. The raw results, which were obtained as OD (Optical Density) values from quadruplicate wells, were then transformed into the final results that are presented as % of dead cells (mean values ±SE) in relation with appropriate DMSO controls. The level of toxicity was defined according to the requirements presented in EN ISO 10993-5:2009(see Table 8).

	Concentration [µM]								
	62.5			31.2			15.6		
Compound	Mean	±SE	Grade Toxicity	Mean	±SE	Grade Toxicity	Mean	±SE	Grade Toxicity
**SCM1**	6.1	0.8	0 lack	3.0	1.5	0 lack	2.5	1.4	0 lack
**SCM2**	0.5	0.5	0 lack	0.0	0.0	0 lack	0.0	0.0	0 lack
**SCM3**	15.6	1.9	1 weak	14.3	1.4	1 weak	6.2	0.3	0 lack
**SCM4**	18.0	1.8	1 weak	1.2	0.8	0 lack	0.5	0.5	0 lack
**SCM5**	31.8	0.5	2 moderate	8.3	3.0	0 lack	2.0	1.2	0 lack
**SCM6**	15.2	0.2	1 weak	4.9	0.4	0 lack	4.3	2.2	0 lack
**SCM7**	14.3	1.2	1 weak	8.2	0.6	0 lack	2.6	2.6	0 lack
**SCM8**	16.5	2.7	1 weak	15.3	0.8	1 weak	12.7	1.5	1 weak
**SCM9**	15.7	0.7	1 weak	7.5	1.1	0 lack	0.1	0.1	0 lack
**SCM 10**	1.9	1.0	0 lack	1.2	0.5	0 lack	0.2	0.2	0 lack

**Table 2:** The effects of the compounds on PHA-induced proliferation of PBMC. The compounds were added to the cultures at concentrations of 50, 25 and 12.5 μM. The proliferative response of the cells in 72 h culture was determined by colorimetric MTT method. Teriflunomide was used as a reference drug. The results are presented as the OD (Optical Density) values from quadruplicate determinations (mean ±SE).

Compound	Concentration [µM]											
	50				25				12.5			
	Optical Density			Inhibition	Optical Density			Inhibition	Optical Density			Inhibition
	Mean		±SE	[%]	Mean		±SE	[%]	Mean		±SE	[%]
**DMSO**	0.364		0.01	0	0.388		0.00	0	0.385		0.01	0
**SCM1**	0.312	*	0.01	14.3	0.353		0.01	9.0	0.401		0.01	−4.2
**SCM2**	0.259	*	0.01	28.8	0.370		0.01	4.6	0.380		0.01	1.3
**SCM3**	0.238	*	0.01	34.6	0.365		0.01	5.9	0.382		0.00	0.8
**SCM4**	0.193	*	0.00	47.0	0.341	*	0.01	12.1	0.383		0.01	0.5
**SCM5**	0.110	*	0.00	69.8	0.279	*	0.00	28.1	0.377		0.01	2.1
**SCM6**	0.351		0.00	3.6	0.383		0.01	1.3	0.371		0.01	3.6
**SCM7**	0.336		0.01	7.7	0.365		0.01	5.9	0.352		0.01	8.6
**SCM8**	0.297	*	0.01	18.4	0.350		0.00	9.8	0.361		0.01	6.2
**SCM9**	0.143	*	0.00	60.7	0.278	*	0.00	28.4	0.322	*	0.01	16.4
**SCM10**	0.228	*	0.01	37.4	0.360		0.01	7.2	0.371		0.01	3.6
**Teriflunomide**	0.226	*	0.01	37.9	0.228	*	0.00	41.2	0.291	*	0.00	24.4

* indicates statistical significance when *p* < 0.05, versus DMSO control. The proliferation inhibition expressed in % was calculated with following formula Inh% = (1 − OD_comp._/OD_DMSO_) × 100.

**Table 3:** Effect of **SCM** compounds on growth of tumor lines. The compounds were added to the cultures at concentrations of 50, 25 and 12.5 μM. The cell lines were incubated for 3 days and cell viability was determined using a MTT colorimetric method. The data are presented as optical density (OD) values from quadruplicate wells ±SE. Control cell cultures contained cisplatin (dissolved in medium only).

		Concentration [μM]											
Cell line	Compound	50				25				12.5			
		Optical Density			Inhibition	Optical Density			Inhibition	Optical Density			Inhibition
		Mean		±SE	[%]	Mean		±SE	[%]	Mean		±SE	[%]
A-431	**Control**	1.020		0.020									
	**DMSO control**	0.459		0.016	0	0.809		0.024	0	0.875		0.020	0
	**SCM5**	0.396		0.013	13.7	0.557	*	0.015	31.1	0.688	*	0.009	21.4
	**SCM9**	0.478		0.022	−4.1	0.641	*	0.027	20.8	0.726	*	0.006	17.0
	**Cisplatin**	0.022	*	0.000	97.8	0.025	*	0.001	97.6	0.066	*	0.002	93.5
HT-29	**Control**	1.147		0.031									
	**DMSO control**	0.685		0.017	0	0.871		0.028	0	1.131		0.039	0
	**SCM5**	0.483	*	0.013	29.5	0.630	*	0.039	27.7	0.853	*	0.034	24.6
	**SCM9**	0.301	*	0.010	56.1	0.533	*	0.021	38.8	0.789	*	0.004	30.2
	**Cisplatin**	0.197	*	0.009	82.8	0.451	*	0.012	60.7	0.675	*	0.026	41.2
L-1210	**Control**	0.769		0.008									
	**DMSO control**	1.172		0.029	0	0.910		0.036	0	1.160		0.051	0
	**SCM5**	0.308	*	0.007	73.7	0.834		0.050	8.4	1.008	*	0.010	13.1
	**SCM9**	0.411	*	0.015	64.9	0.894		0.018	1.8	1.043		0.028	10.1
	**Cisplatin**	0.018	*	0.000	97.7	0.020	*	0.000	97.4	0.030	*	0.001	96.1

* indicates statistical significance when *p* < 0.05, versus DMSO control. The growth cancer cell inhibition expressed in % was calculated with following formula Inh% = (1 − OD_comp._/OD_DMSO_) × 100.

**Table 4:** The effects of **SCM** compounds on TNF α production by LPS-stimulated human whole blood cultures. Human whole blood cultures were incubated overnight with the compounds at concentrations of 25, 10 and 1 μM and 100 ng/mL of LPS. TNF α levels in the supernatants were determined by ELISA kit. The results are shown as percentage of inhibition versus DMSO control (mean values from three independent experiments ±SE).

Compound	Concentration [µM]	% Inhibition		
		Mean	*p*	±SE
**SCM5**	25	-		-
	10	-		-
	1	-		-
**SCM9**	25	21.3	*	2.7
	10	6.9		3.1
	1	-		-

* indicates statistical significance when *p* < 0.05, versus DMSO control; (-) denotes lack of inhibition.

**Table 5:** Effect of **SCM** compounds on MAP kinase expression in Jurkat cells. Glyceraldehyde-3-phosphate dehydrogenase (GAPDH) was used as a housekeeping gene to normalize values obtained for the target genes. The dimensionless values presented in the table reflect relative fold changes in response of the cells to **SCM** compounds, when the GAPDH gene expression was a control and assumed as equal to one.

Compound	ERK-1	ERK-2	p38α	p38β	p38γ	p38δ	JNK
**SCM5**	0.09	0.13	0.84	0.48	0.33	0.54	0.35
**SCM9**	0.80	0.68	1.45	1.40	0.93	0.67	2.04

**Table 6:** Effect of **SCM** compounds on MAP kinase expression in WEHI 231 cells. For description see Table 5 legend.

Compound	ERK-1	ERK-2	p38α	p38β	p38γ	p38δ	JNK
**SCM 5**	1.04	0.85	1.38	1.20	2.68	3.74	1.20
**SCM 9**	1.01	0.52	0.65	1.01	1.36	1.98	0.65

**Table 7:** Effect of **SCM** compounds on expression of selected signaling molecules in WEHI 231 cells. For description see Table 5 legend.

Compound	Casp-3	Casp-8	Casp-9	NFκB	Bcl-2	Fas
**SCM 5**	0.97	8.20	23.13	17.91	10.32	8.82
**SCM 9**	1.16	0.80	0.86	0.62	0.74	0.61

**Table 8:** Criteria of toxicity effect based on changes in cell morphology (according to: EN ISO 10993-5:2009. Biological evaluation. Part 5: Test for in vitro cytotoxicity. International Organization for Standardization, Geneva, Switzerland, 2009).

Grade	Toxicity	Cell Morphology
**0**	lack	Discrete intracytoplasmic granules, no evidence cell lysis, lack of inhibition of cell growth
**1**	weak	Not more than 20% of rounded, shrunk cells, separating from the substrate without densities of cytoplasm, individual cells disrupted
**2**	moderate	Not more than 50% of rounded cells, no evidence of granules, vast cell lysis and empty spaces between cells
**3**	average	Not more than 70% of rounded cells, cells underwent lysis
**4**	strong	Almost completely or completely damage cell culture

## Supplementary Materials

The Supplementary Materials are available online. Such as detailed analysis of ESI-MS spectra, visualizations of IR and NMR spectra and other additional information associated with this paper can be found in the Supplementary Material file (pdf). CCDC 2005200 contains the supplementary crystallographic data for this paper. These data can be obtained free of charge via www.ccdc.cam.ac.uk/structures/ (or from the Cambridge Crystallographic Data Centre, 12, Union Road, Cambridge CB2 1EZ, UK; fax: +44 1223 336033) or e-mail: deposit@ccdc.cam.ac.uk.

## References

[1] Johnson T.B. (1905). Researches on pyrimidines: 2-ethylmercapto-5-amino-6-oxypyrimidine. Am. Chem. J..

[2] Block M.H., Harrison A., Hargreaves R.B. (1993). Preparation of Furyl-Substituted Purines, Oxazolopyrimidines and Pteridines as Adenosine Antagonist. European Patent.

[3] Martin-Kohler A., Widmer J., Bold G., Meyer T., Séquin U., Traxler P. (2004). Furo [2,3-*d*] pyrimidines and oxazolo [5,4-*d*] pyrimidines as inhibitors of receptor tyrosine kinases (RTK). Helv. Chim. Acta.

[4] Bauser M., Delapierre G., Hauswald M., Flessner T., D’Urso D., Hermann A., Beyreuther B., De Vry J., Spreyer P., Reissmüller E. (2004). Discovery and opitimalization of 2-aryl oxazolo-pyrimidines as adenosine kinase inhibitors using liquid phase paralel synthesis. Bioorg. Med. Chem. Lett..

[5] Robertus J., Kerwin S.M., Yan X. (2001). Ricin Inhibitors and Methods for Use Thereof. WO Patent.

[6] Miller D.J., Ravikumar K., Shen H., Suh J.-K., Kerwin S.M., Robertus J.D. (2002). Structure-based design and characterization of novel platforms for ricin and Shiga toxin inhibition. J. Med. Chem..

[7] De Jonghe S., Gao L.-J., Herdewijn P., Herman J., Jang M., Leyssen P., Louat T., Neyts J., Pannecouque C., Vanderhoydonck B. (2011). Preparation of Bicyclic Heterocycles as Antiviral Agents. WO Patent.

[8] Liu J., Deng Y.H., Yang L., Chen Y., Lawali M., Sun L.P., Liu Y. (2015). CPU-12, a novel synthesized oxazolo [5,4-*d*] pyrimidine derivative, showed superior anti-angiogenic activity. J. Pharmacol. Sci..

[9] Jang M.-Y., Lin Y., De Jonghe S., Gao L.-J., Vanderhoydonck B., Froeyen M., Rozenski J., Herman J., Louat T., Van Belle K. (2011). Discovery of 7-*N*-piperazinylthiazolo [5,4-*d*] pyrimidine analogues as a novel class of immunosuppressive agents with in vivo biological activity. J. Med. Chem..

[10] Cai S.X., Kemnitzer W.E., Sirisoma S., Zhang H.-Z. (2008). *N*-Aryl-Isoxazolopyrimidin-4-Amines and Related Compounds as Activators of Caspases and Inducers of Apoptosis and the Use Thereof. WO Patent.

[11] Claiborne C.F., Critchley S., Langston S.P., Olhava E.J., Peluso S., Weatherhead G.S., Vyskocil S., Visiers I., Mizutani H., Cullis C. (2008). Heteroaryl Compounds Useful as Inhibitors of E1 Activating Enzymes. WO Patent.

[12] Regiec A., Wojciechowski P., Pietraszko A., Mączyński M. (2018). Infrared spectra and other properties predictions of 5-amino-3-methyl-4-isoxazolecarbohydrazide with electric field simulation using CPC model. J. Mol. Struct..

[13] Regiec A., Machoń Z., Międzybrodzki R., Szymaniec S. (2006). New isothiazole derivatives: Synthesis, reactivity, physicochemical properties and pharmacological activity. Arch. Pharm. Chem. Life Sci..

[14] Lipnicka U., Regiec A., Sułkowski E., Zimecki M. (2005). New amides of 5-acylamino-3-methyl-4-isothiazolecarboxylic acid and their immunotropic activity. Arch. Pharm. Chem. Life Sci..

[15] Lipnicka U., Regiec A., Machoń Z. (1994). Synthesis and cytotoxic activity of new isothiazolo [5,4-*d*] pyrimidine derivatives. Pharmazie.

[16] Regiec A., Mastalarz H., Międzybrodzki R., Śmietańska K., Jasztold-Howorko R. (2006). *N*-Arylamides of 4-amino-1-methyl-5-imidazolecarboxylic acid-their synthesis and biological activity. Lett. Drug Des. Discov..

[17] Mączyński M., Zimecki M., Taraszkiewicz M., Ryng S. (2008). Synthesis, immunological activity and computational study of 5-amino-3-methyl-4-isoxazolecarboxylic acid semicarbazides and thiosemicarbazides. Acta Pol. Pharm..

[18] Mączyński M., Ryng S., Artym J., Kocięba M., Zimecki M., Brudnik K., Jodkowski J.T. (2014). New lead structures in the isoxazole system: Relationship between quantum chemical parameters and immunological activity. Acta Pol. Pharm..

[19] Drynda A., Mączyński M., Ryng S., Obmińska-Mrukowicz B. (2014). In vitro immunomodulatory effects of 5-amino-3-methyl-4-isoxazolecarboxylic acid hydrazide on the cellular immune response. Immunopharmacol. Immunotoxicol..

[20] Drynda A., Obmińska-Mrukowicz B., Mączyński M., Ryng S. (2015). The effect of 5-amino-3-methyl-4-isoxazolecarboxylic acid hydrazide on lymphocyte subsets and humoral immune response in SRBC-immunized mice. Immunopharmacol. Immunotoxicol..

[21] Mączyński M., Artym J., Kocięba M., Sochacka-Ćwikła A., Drozd-Szczygieł E., Ryng S., Zimecki M. (2016). Synthesis and immunoregulatory properties of selected 5-amino-3-methyl-4 isoxazolecarboxylic acid benzylamides. Acta Pol. Pharm..

[22] Zimecki M., Artym J., Kocięba M., Obmińska-Mrukowicz B., Mączyński M., Ryng S. (2012). Restoration of immune system function is accelerated in immunocompromised mice by the B-cell-tropic isoxazole R-11. Pharmacol. Rep..

[23] Zimecki M., Mączyński M., Artym J., Ryng S. (2008). Closely related isoxazoles may exhibit opposite immunological activities. Acta Pol. Pharm..

[24] Mączyński M., Artym J., Kocięba M., Kochanowska I., Ryng S., Zimecki M. (2016). Anti-inflammatory properties of an isoxazole derivative—MZO-2. Pharmacol. Rep..

[25] Zimecki M., Ryng S., Mączyński M., Chodaczek G., Kocięba M., Kuryszko J., Kaleta K. (2006). Immunosuppressory activity of an isoxazolo [5,4-e] triazepine-compound RM-33 II. Effects on the carrageenan-induced inflammation. Pharmacol. Rep..

[26] De Coen L.M., Roman B.I., Movsisyan M., Heugebaert T.S.A., Stevens C.V. (2018). Synthesis and biological activity of oxazolopyrimidines. Eur. J. Org. Chem..

[27] Shaw G., Sugowdz G. (1954). The hydrogenation of 5-aminoizooxazoles. A new synthesis of pyrimidines. J. Chem. Soc..

[28] Regiec A., Gadziński P. (2014). New Methods for Preparing of Esters of 2-Cyano-3-Alkoxy-2-Butenoate Acids. Polish Patent.

[29] Freeman F., Kim D.S.H.L. (1989). Preparation of 2-alkyl- and 2-aryl-5-amino-4-cyano-1,3-oxazoles. Tetrahedron Lett..

[30] Günther H. (1980). NMR Spectroscopy. An Introduction.

[31] Ohtsuka Y. (1973). Study on oxazolopyrimidines. VI. Formation of 3-substitutes xanthines via 7(6H)-iminooxazolopirymidines. Bull. Chem. Soc. Jpn..

[32] Cossi M., Rega N., Scalmani G., Barone V. (2003). Energies, structures, and electronic properties of molecules in solution with the C-PCM solvation model. J. Comp. Chem..

[33] Williamson S.I., Wannemuehler M.J., Jirillo E., Pritchard D.G., Michalek S.M., McGhee J.R. (1984). LPS regulation of the immune response: Separate mechanisms for murine B cell activation by lipid A (direct) and polysaccharide (macrophage-dependent) derived from Bacteroides LPS. J. Immunol..

[34] Harrington E.O., Smeglin A., Parks N., Newton J., Rounds S. (2000). Adenosine induces endothelial apoptosis by activating protein tyrosine phosphatase: A possible role of p38alpha. Am. J. Physiol. Lung Cell. Mol. Physiol..

[35] Wu Y.J., Su T.R., Dai G.F., Su J.H., Liu C.I. (2019). Flaccidoxide-13-acetate-induced apoptosis in human bladder cancer cells is through activation of p38/JNK, mitochondrial dysfunction, and endoplasmic reticulum stress regulated pathway. Mar. Drugs.

[36] Sutherland C.L., Heath A.W., Pelech S.L., Young P.R., Gold M.R. (1996). Differential activation of the ERK, JNK, and p38 mitogen-activated protein kinases by CD40 and the B cell antigen receptor. J. Immunol..

[37] Chaudhary P.M., Eby M.T., Jasmin A., Kumar A., Liu L., Hood L. (2000). Activation of the NF-kappaB pathway by caspase 8 and its homologs. Oncogene.

[38] Zhao Y., Lei M., Wang Z., Qiao G., Yang T., Zhang J. (2014). TCR-induced, PKC-θ-mediated NF-κB activation is regulated by a caspase-8-caspase-9-caspase-3 cascade. Biochem. Biophys. Res. Commun..

[39] Fan M., Goodwin M., Vu T., Brantley-Finley C., Gaarde W.A., Chambers T.C. (2000). Vinblastine-induced phosphorylation of Bcl-2 and Bcl-XL is mediated by JNK and occurs in parallel with inactivation of the Raf-1/MEK/ERK cascade. J. Biol. Chem..

[40] Juo P., Kuo C.J., Yuan J., Blenis J. (1998). Essential requirement for caspase-8/FLICE in the initiation of the Fas-induced apoptotic cascade. Curr. Biol..

[41] Gutierrez-Sanmartin D., Varela-Ledo E., Aguilera A., Romero-Yuste S., Romero-Jung P., Gomez-Tato A., Regueiro B.J. (2008). Implication of p38 mitogen-activated protein kinase isoforms (alpha, beta, gamma and delta) in CD4+ T-cell infection with human immunodeficiency virus type I. J. Gen. Virol..

[42] Nunn S., Nishikida K. (2008). Advanced ATR Correction Algorithm.

[43] (2018). CrysAlis, Pro.

[44] Sheldrick G.M. (2008). A short history of SHELX. Acta Crystallogr. Sect. A.

[45] Sheldrick G.M. (2015). Crystal structure refinement with SHELXL. Acta Crystallogr. Sect. C.

[46] Brandenburg K. (2012). Diamond. Version 3.2i.

[47] Becke A.D. (1996). Density-functional thermochemistry. IV. A new dynamical correlation functional and implications for exact-exchange mixing. J. Chem. Phys..

[48] Lee C.T., Yang W.T., Parr R.G. (1988). Development of the Colle-Salvetti correlation-energy formula into a functional of the electron density. Phys. Rev. B.

[49] Frisch M.J., Trucks G.W., Schlegel H.B., Scuseria G.E., Robb M.A., Cheeseman J.R., Scalmani G., Barone V., Petersson G.A., Nakatsuji H. (2016). Gaussian 16 (Revision, A.03).

[50] Ochterski J.W. (2000). Thermochemistry in Gaussian.

[51] Hansen M.B., Nielsen S.E., Berg K. (1989). Reexamination and further development of a precise and rapid dye method for measuring cell growth/cell kill. J. Immunol. Methods.

