# Supplementary material for: Synthesis and Biological Activity of New 7-Amino-oxazolo[5,4-d]Pyrimidine Derivatives
Source: Molecules. 2020 Aug 4;25(15):3558. doi: 10.3390/molecules25153558 (PMC7436121; doi:10.3390/molecules25153558)
Supplement: Supplementary file 1 [file molecules-25-03558-s001.zip › Supplementary material.pdf]

SUPPLEMENTARY MATERIAL

Synthesis and biological activity of new 7-aminooxazolo[5,4-*d*]pyrimidine derivatives

Aleksandra Sochacka-Ćwikła [1], Andrzej Regiec [1,*], Michał Zimecki [2], Jolanta Artym [2], Ewa Zaczyńska [2], Maja Kocięba [2], Iwona Kochanowska [2], Iwona Bryndal [3], Anna Pyra [4] and Marcin Mączyński [1]

[1] Department of Organic Chemistry, Faculty of Pharmacy, Wroclaw Medical University, 211A Borowska Street, 50-556 Wroclaw, Poland; aleksandra.sochacka-cwikla@umed.wroc.pl (A.S.-Ć.); marcin.maczynski@umed.wroc.pl (M.M.)

[2] Department of Experimental Therapy, Institute of Immunology and Experimental Therapy, Polish Academy of Sciences, 12 Rudolf Weigl Street, 53-114 Wroclaw, Poland; michal.zimecki@hirszfeld.pl (M.Z.); jolanta.artym@hirszfeld.pl (J.A.); ewa.zaczynska@hirszfeld.pl (E.Z.); maja.kocieba@hirszfeld.pl (M.K.); iwona.kochanowska@hirszfeld.pl (I.K.)

[3] Department of Drug Technology, Faculty of Pharmacy, Wroclaw Medical University, 211A Borowska Street, 50-556 Wroclaw, Poland; iwona.bryndal@umed.wroc.pl

[4] Faculty of Chemistry, University of Wroclaw, 14 Joliot-Curie, 50-383 Wroclaw, Poland; anna.pyra@uwr.edu.pl

*Correspondence: andrzej.regiec@umed.wroc.pl, Tel./Fax: +48717840347

TABLE OF CONTENTS

Chemistry 4

Electrospray Mass Ionization (ESI) measurements of intermediates (2-4), oxazolo[5,4-*d*]pyrimidines (5a-5l) and *N'*-cyanooxazolylacetamidines (7m) 4

Analysis and Visualizations of Electrospray Mass Ionization (ESI) spectra of the compounds 2-4 4

Analysis and Visualizations of Electrospray Mass Ionization (ESI) spectra of the compounds 5a-5l 6

Analysis and Visualizations of Electrospray Mass Ionization (ESI) spectra of compound 7m 15

Visualizations of Nuclear magnetic resonance (NMR) and Infrared (IR) spectra of compound 2 16

Visualizations of Nuclear magnetic resonance (NMR) and Infrared (IR) spectra of compound 3 17

Visualizations of Nuclear magnetic resonance (NMR) and Infrared (IR) spectra of compound 4 19

Visualizations of Nuclear magnetic resonance (NMR) and Infrared (IR) spectra of compound 5a 20

Visualizations of Nuclear magnetic resonance (NMR) and Infrared (IR) spectra of compound 5b 23

Visualizations of Nuclear magnetic resonance (NMR) and Infrared (IR) spectra of compound 5c 26

Visualizations of Nuclear magnetic resonance (NMR) and Infrared (IR) spectra of compound 5d 29

Visualizations of Nuclear magnetic resonance (NMR) and Infrared (IR) spectra of compound 5e 30

Visualizations of Nuclear magnetic resonance (NMR) and Infrared (IR) spectra of compound 5f 32

Visualizations of Nuclear magnetic resonance (NMR) and Infrared (IR) spectra of compound 5g 33

Visualizations of Nuclear magnetic resonance (NMR) and Infrared (IR) spectra of compound 5h 35

Visualizations of Nuclear magnetic resonance (NMR) and Infrared (IR) spectra of compound 5i 36

Visualizations of Nuclear magnetic resonance (NMR) and Infrared (IR) spectra of compound 5j 40

Visualizations of Nuclear magnetic resonance (NMR) and Infrared (IR) spectra of compound 5k 42

Visualizations of Nuclear magnetic resonance (NMR) and Infrared (IR) spectra of compound 5l 43
Visualizations of Nuclear magnetic resonance (NMR) and Infrared (IR) spectra of compound 7m 45
Single crystal X-ray diffraction measurement of compound 5h 46
Biology 49

CHEMISTRY

Electrospray Mass Ionization (ESI) measurements of intermediates (2-4), oxazolo[5,4-*d*]pyrimidines (5a-5l) and *N'*-cyanooxazolylacetamidines (7m)

Electrospray Mass Ionization (ESI) spectra of the all compounds were recorded with the compact^{TM} Electrospray Ionisation-Quadrupole-Time of Flight (ESI-Q-TOF) Mass Spectrometer (Bruker Daltonics) in methanol solutions in positive ionization. The samples of the compounds were dissolved in methanol in eppendorf tubes and immediately measured. Tandem mass spectrometry MS/MS were performed with low-energy collision-induced dissociation (CID), nitrogen as collision gas. Collision energy in the range of 5-30 eV.

HR-ESI-MS measurement conditions:

- mass spectrometer resolution above 16000 (in our measurements, the resolution = 23500)
- mass measurement error below |±5| ppm (indicating a good mass measurement accuracy)

Analysis and Visualizations of Electrospray Mass Ionization (ESI) spectra of the compounds 2-4

Table S1. Comparative set of masses of quasi-parent molecular $[M+Na]^+$ ions (as base peaks) of the compounds **2-3** and quasi-parent molecular $[M+H]^+$ ion (as base peak) of compound **4**. Errors associated with mass measurements of the compounds **2-4**.

No.	Calculated m/z for		Found m/z for base peaks (percentage relative intensity)	Mass measurement error (ppm)
	$[M+H]^+$ ion	$[M+Na]^+$ ion		
**2**	-	228.0491	228.0499 (100%)	3.51
**3**	-	298.0911	298.0975 (100%)	21.47
**4**	346.1510	-	346.1445 (100%)	-18.78

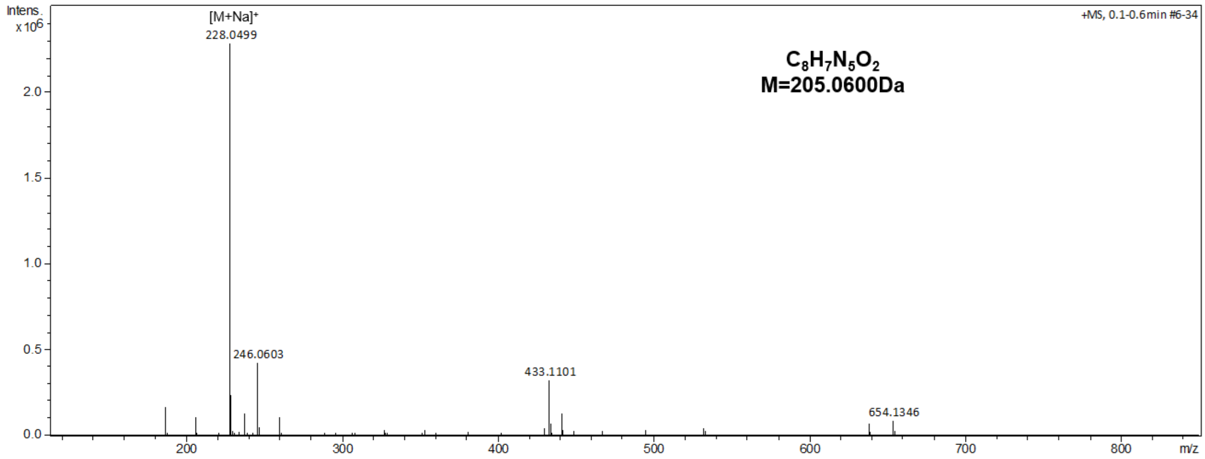

Figure S1. HR-ESI-MS spectrum of compound **2**.

Table S2. Analysis of other quasi-parent molecular ions of compound **2**.

Other quasi-parent molecular ions of **2**	Calculated m/z	Found		Mass measurement error (ppm)
		m/z	Relative intensity (%) to base peak	
$[M+H]^+$	206.0672	206.0675	4.77	1.46
$[M+CH_3OH+H]^+$	238.0935	238.0938	5.59	1.26
$[M+H_2O+Na]^+$	246.0598	246.0603	18.58	2.03
$[M+CH_3OH+Na]^+$	260.0754	260.0757	4.59	1.15
$[2M+Na]^+$	433.1092	433.1101	13.99	2.08
$[2M+K]^+$	449.0831	449.0755	1.04	-16.92
$[3M+Na]^+$	638.1691	638.1685	2.99	-0.94
$[3M+K]^+$	654.1431	654.1346	3.68	-12.99

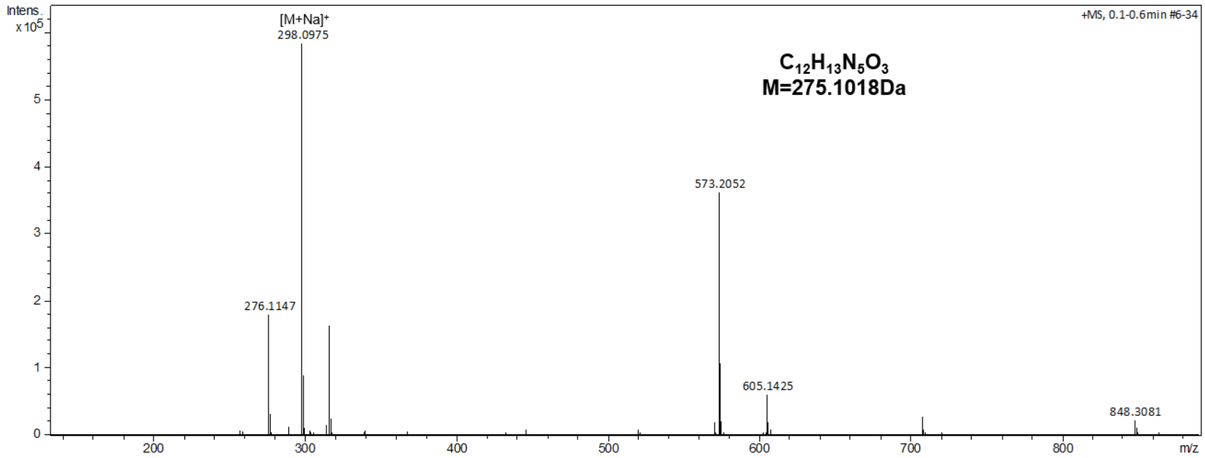

Figure S2. ESI-MS spectrum of compound **3**.

Table S3. Analysis of other quasi-parent molecular ions of compound **3**.

Other quasi-parent molecular ions of **3**	Calculated m/z	Found	
		m/z	Relative intensity (%) to base peak
$[M+H]^+$	276.1091	276.1147	30.89
$[M+K]^+$	314.0650	314.0708	2.55
$[M+H_2O+Na]^+$	316.1016	316.1079	27.80
$[2M+Na]^+$	573.1929	573.2052	61.78
$[2M+CH_3OH+Na]^+$	605.2191	605.1425	10.27
$[3M+Na]^+$	848.2947	848.3081	3.69

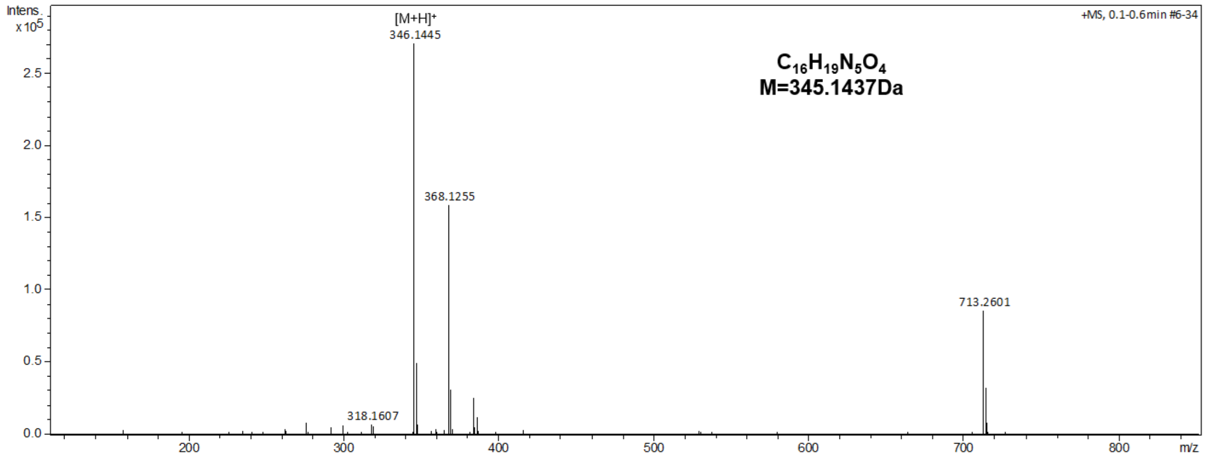

Figure S3. ESI-MS spectrum of compound **4**.

Table S4. Analysis of other quasi-parent molecular ions of compound **4**.

Other quasi-parent molecular ions of **4**	Calculated m/z	Found	
		m/z	Relative intensity (%) to base peak
$[M\text{-}CO+H]^+$	318.1561	318.1607	2.72
$[M+Na]^+$	368.1329	368.1255	58.77
$[M+K]^+$	384.1069	384.0990	9.25
$[2M+Na]^+$	713.2766	713.2601	31.64

Analysis and Visualizations of Electrospray Mass Ionization (ESI) spectra of the compounds 5a-5l

Table S5. Comparative set of masses of quasi-parent molecular $[M+H]^+$ ions (as base peaks) of the compounds **5a-5l**. Errors associated with mass measurements of the compounds **5a-5l**.

No.	Calculated m/z for $[M+H]^+$ ion	Found m/z for $[M+H]^+$ ion (percentage relative intensity)	Mass measurement error (ppm)
**5a**	261.1094	261.1081 (100%)	-4.98
**5b**	275.1251	275.1246 (100%)	-1.82
**5c** (method A)	289.1408	289.1407 (100%)	-0.35
**5c** (method B)	289.1408	289.1454 (100%)	15.91
**5d**	303.1564	303.1556 (100%)	-2.64
**5e**	317.1720	317.1717 (100%)	-0.95
**5f**	291.1200	291.1195 (100%)	-1.72
**5g**	305.1357	305.1352 (100%)	-1,64
**5h**	360.1779	360.1768 (100%)	-3.05
**5i** (method A)	332.1829	332.1821 (100%)	-2.41
**5i** (method B)	332.1829	332.1824 (100%)	-1,51
**5i** (method C)	332.1829	332.1848 (100%)	5.72
**5j**	318.1673	318.1664 (100%)	-2.83
**5k**	289.1408	289.1396 (100%)	-4.15
**5l**	329.1720	329.1706 (100%)	-4.25

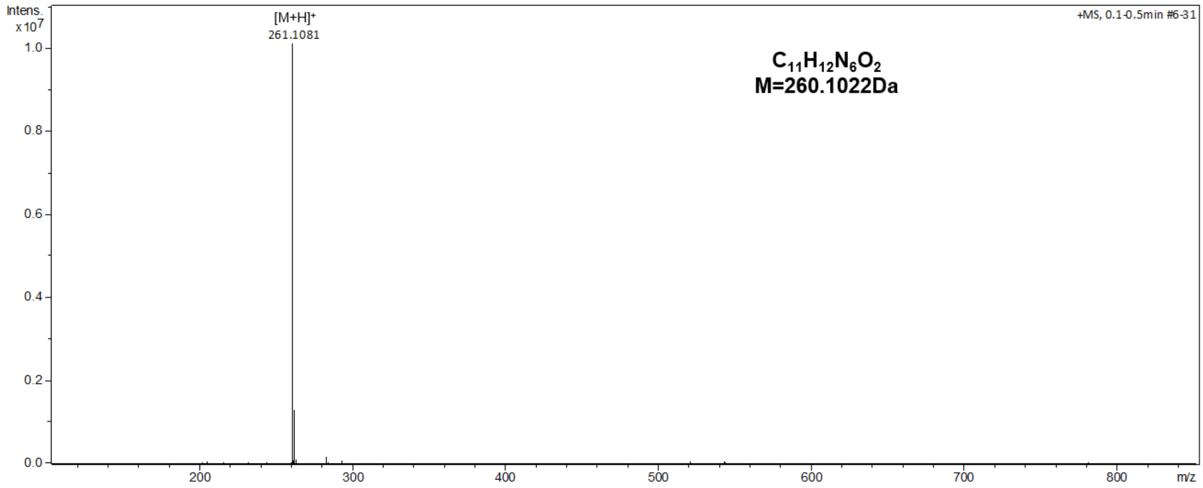

Figure S4. HR-ESI-MS spectrum of compound **5a**.

Table S6. Analysis of other quasi-parent molecular ions of compound **5a**.

Other quasi-parent molecular ions of **5a**	Calculated m/z	Found		Mass measurement error (ppm)
		m/z	Relative intensity (%) to base peak	
$[M+Na]^+$	283.0914	283.0904	1.54	-3.53
$[M+CH_3OH+H]^+$	293.1357	293.1344	0.91	-4.43
$[2M+Na]^+$	543.1936	543.1927	0.57	-1.66

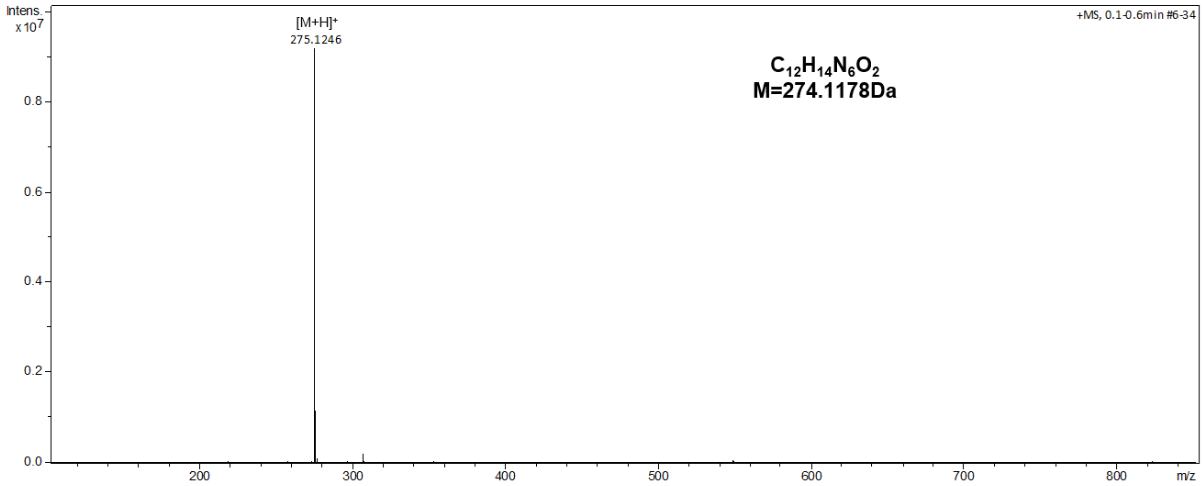

Figure S5. HR-ESI-MS spectrum of compound **5b**.

Table S7. Analysis of other quasi-parent molecular ions of compound **5b**.

Other quasi-parent molecular ions of **5b**	Calculated m/z	Found		Mass measurement error (ppm)
		m/z	Relative intensity (%) to base peak	
$[M+CH_3OH+H]^+$	307.1513	307.1511	2.26	-0.65

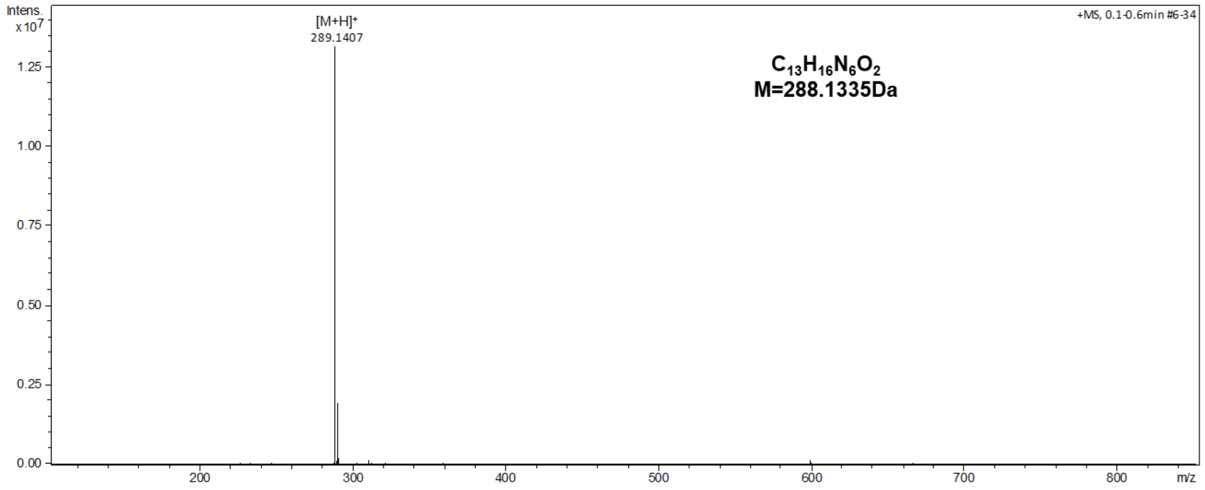

Figure S6. HR-ESI-MS spectrum of compound **5c** (method A).

Table S8. Analysis of other quasi-parent molecular ions of compound **5c** (method A).

Other quasi-parent molecular ions of **5c**	Calculated m/z	Found		Mass measurement error (ppm)
		m/z	Relative intensity (%) to base peak	
$[M+Na]^+$	311.1227	311.1233	1.19	1.93
$[M+CH_3OH+H]^+$	321.1669	321.1674	0.29	1.56
$[2M+Na]^+$	599.2562	599.2584	1.16	3.67

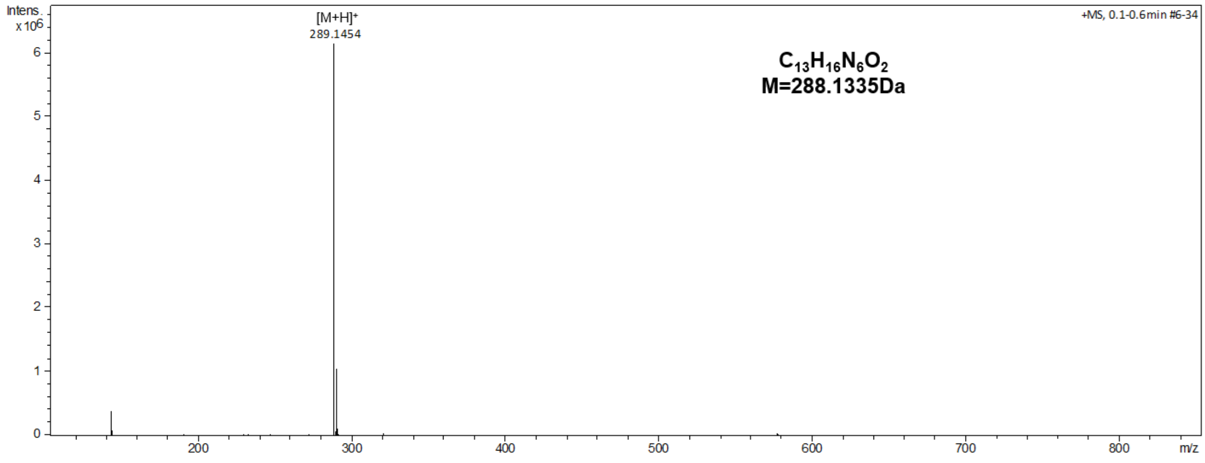

Figure S7. ESI-MS spectrum of compound **5c** (method B).

Table S9. Analysis of other quasi-parent molecular ions of compound **5c** (method B).

Other quasi-parent molecular ions of **5c**	Calculated m/z	Found	
		m/z	Relative intensity (%) to base peak
$[M+CH_3OH+H]^+$	321.1669	321.1710	0.62
$[2M+H]^+$	577.2742	577.2820	0.61

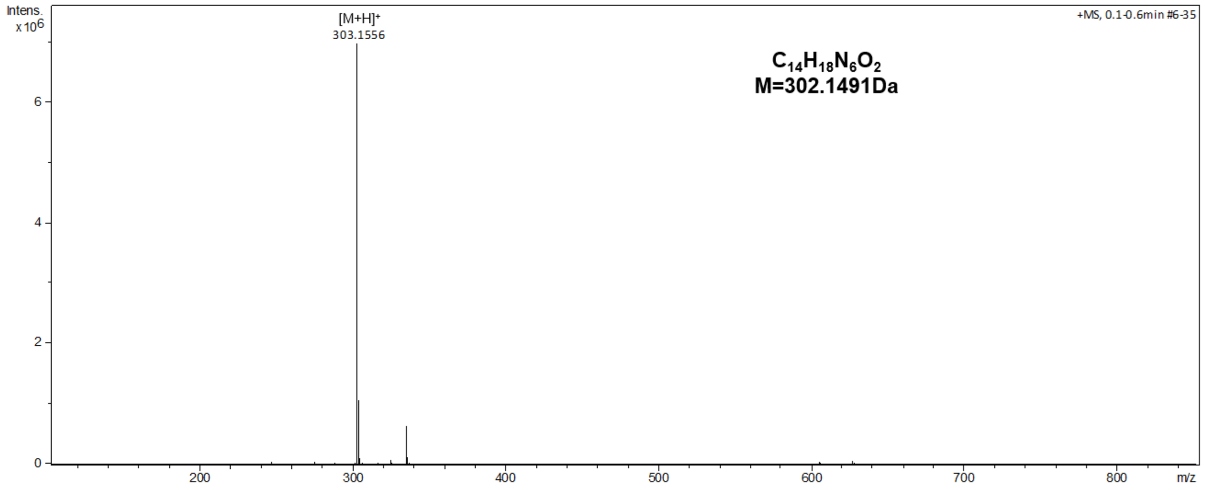

Figure S8. HR-ESI-MS spectrum of compound **5d**.

Table S10. Analysis of other quasi-parent molecular ions of compound **5d**.

Other quasi-parent molecular ions of **5d**	Calculated m/z	Found		Mass measurement error (ppm)
		m/z	Relative intensity (%) to base peak	
$[M+Na]^+$	325.1383	325.1376	0.99	-2.15
$[M+CH_3OH+H]^+$	335.1826	335.1825	9.11	-0.30

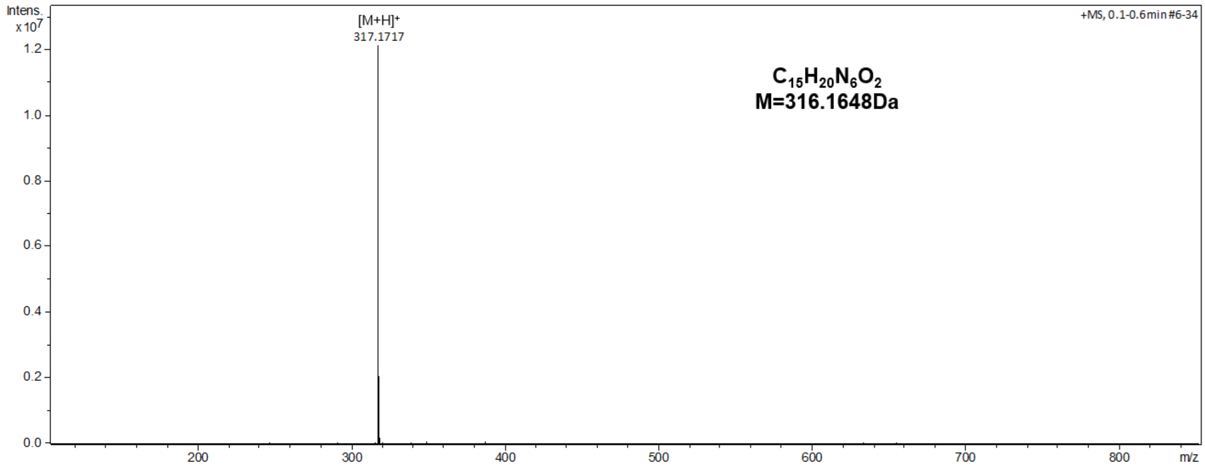

Figure S9. HR-ESI-MS spectrum of compound **5e**.

Table S11. Analysis of other quasi-parent molecular ions of compound **5e**.

Other quasi-parent molecular ions of **5e**	Calculated m/z	Found		Mass measurement error (ppm)
		m/z	Relative intensity (%) to base peak	
$[M+Na]^+$	339.1540	339.1535	0.17	-1.47
$[M+CH_3OH+H]^+$	349.1982	349.1977	0.51	-1.43
$[2M+H]^+$	633.3368	633.3373	0.26	0.79
$[2M+Na]^+$	655.3188	655.3195	0.23	1.07

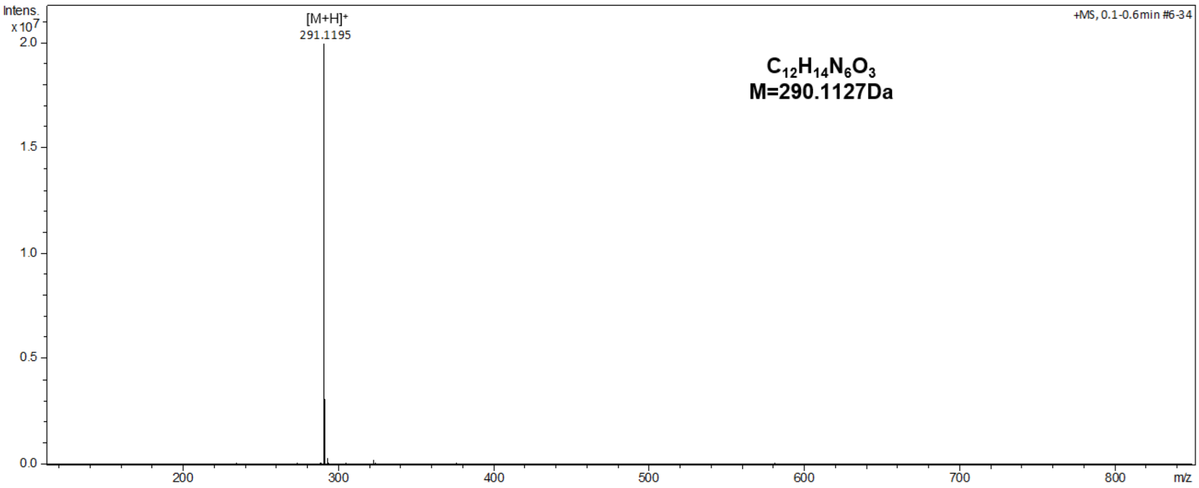

Figure S10. HR-ESI-MS spectrum of compound **5f**.

Table S12. Analysis of other quasi-parent molecular ions of compound **5f**.

Other quasi-parent molecular ions of **5f**	Calculated m/z	Found		Mass measurement error (ppm)
		m/z	Relative intensity (%) to base peak	
$[M+CH_3OH+H]^+$	323.1462	323.1463	1.00	0.31
$[2M+H]^+$	633.3368	633.3372	1.52	0.63

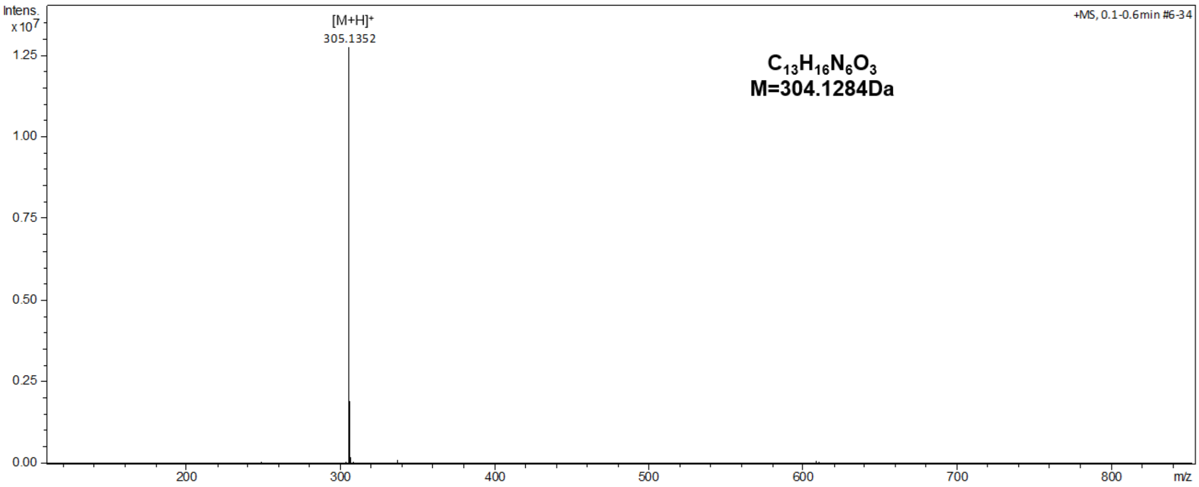

Figure S11. HR-ESI-MS spectrum of compound **5g**.

Table S13. Analysis of other quasi-parent molecular ions of compound **5g**.

Other quasi-parent molecular ions of **5g**	Calculated m/z	Found		Mass measurement error (ppm)
		m/z	Relative intensity (%) to base peak	
$[M+CH_3OH+H]^+$	337.1619	337.1620	0.75	0.30

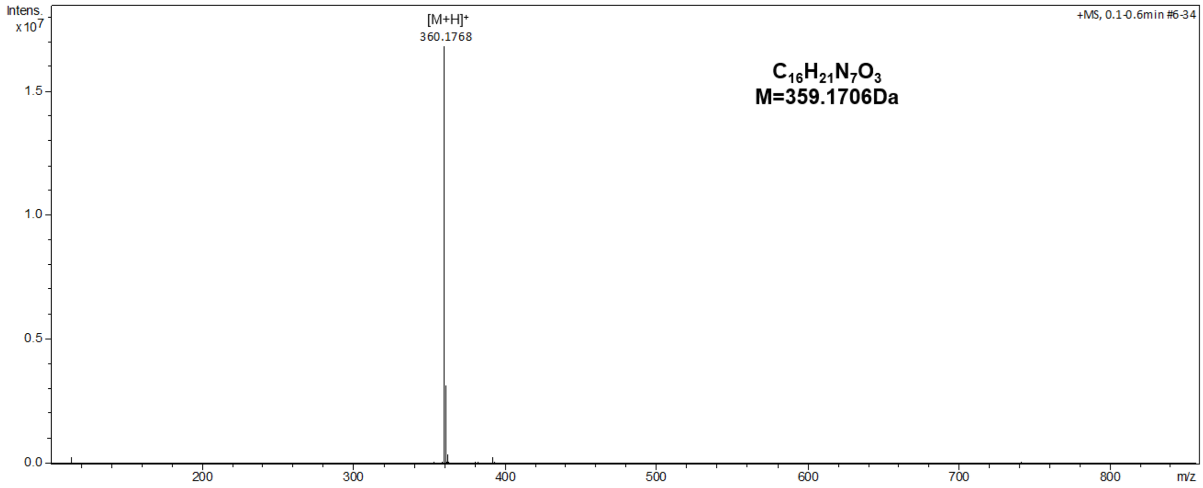

Figure S12. HR-ESI-MS spectrum of compound **5h**.

Table S14. Analysis of other quasi-parent molecular ions of compound **5h**.

Other quasi-parent molecular ions of **5h**	Calculated m/z	Found		Mass measurement error (ppm)
		m/z	Relative intensity (%) to base peak	
$[M+Na]^+$	382.1598	382.1594	0.12	-1.05
$[M+CH_3OH+H]^+$	392.2041	392.2041	2.02	0.00

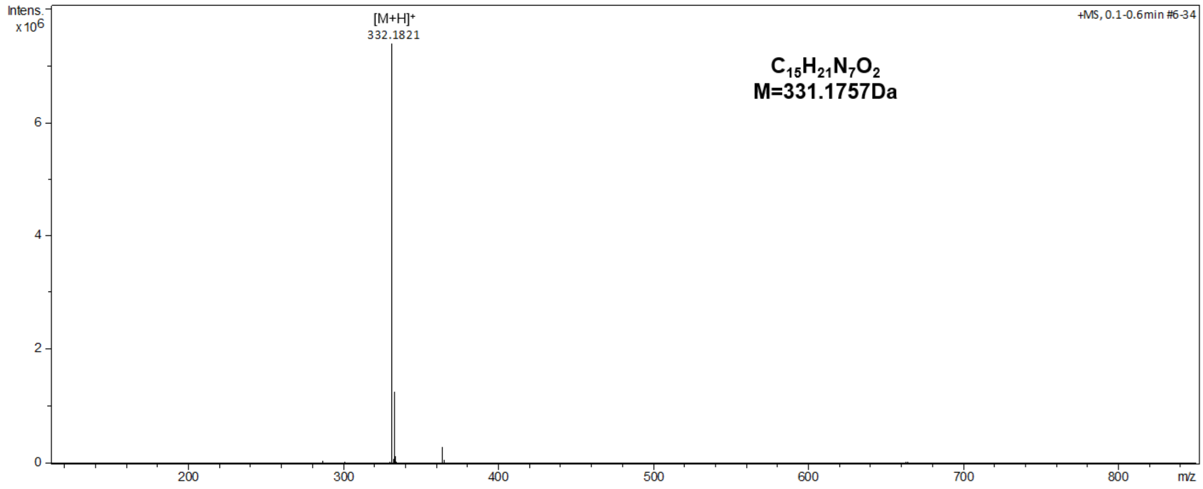

Figure S13. HR-ESI-MS spectrum of compound **5i** (method A).

Table S15. Analysis of other quasi-parent molecular ions of compound **5i** (method A).

Other quasi-parent molecular ions of **5i**	Calculated m/z	Found		Mass measurement error (ppm)
		m/z	Relative intensity (%) to base peak	
$[M+CH_3OH+H]^+$	364.2092	364.2089	3.95	-0.82

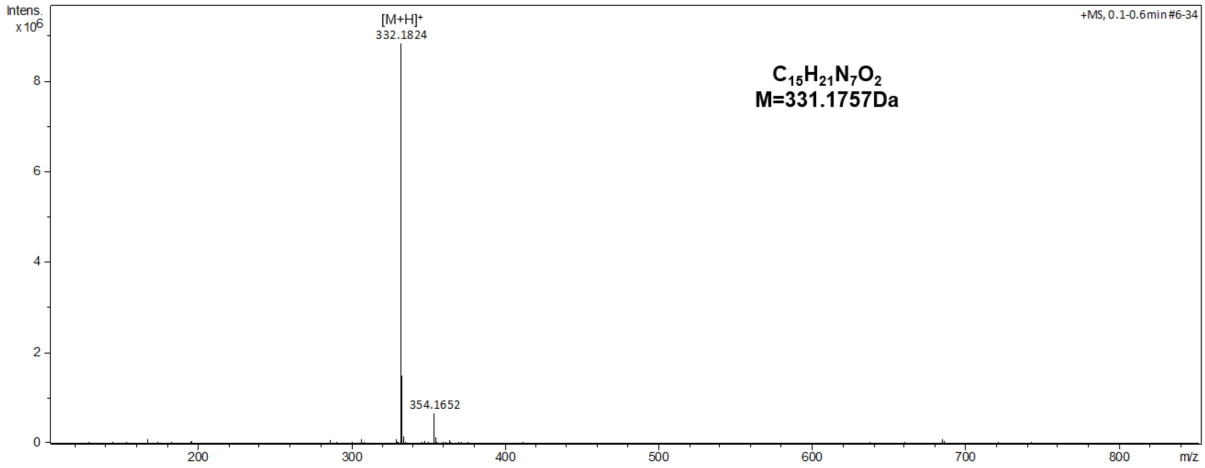

Figure S14. HR-ESI-MS spectrum of compound **5i** (method B).

Table S16. Analysis of other quasi-parent molecular ions of compound **5i** (method B).

Other quasi-parent molecular ions of **5i**	Calculated m/z	Found		Mass measurement error (ppm)
		m/z	Relative intensity (%) to base peak	
$[M+Na]^+$	354.1649	354.1652	7.50	0.85
$[M+CH_3OH+H]^+$	364.2092	364.2085	0.81	-1.92

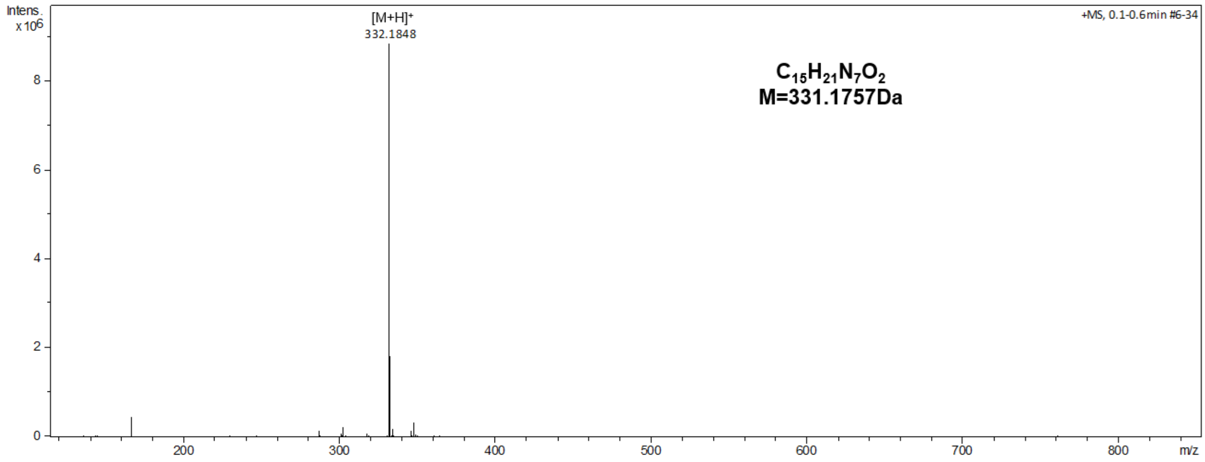

Figure S15. ESI-MS spectrum of compound **5i** (method C).

Table S17. Analysis of other quasi-parent molecular ions of compound **5i** (method C).

Other quasi-parent molecular ions of **5i**	Calculated m/z	Found	
		m/z	Relative intensity (%) to base peak
$[M+2H]^{2+}$	166.5951	166.5949	5.06

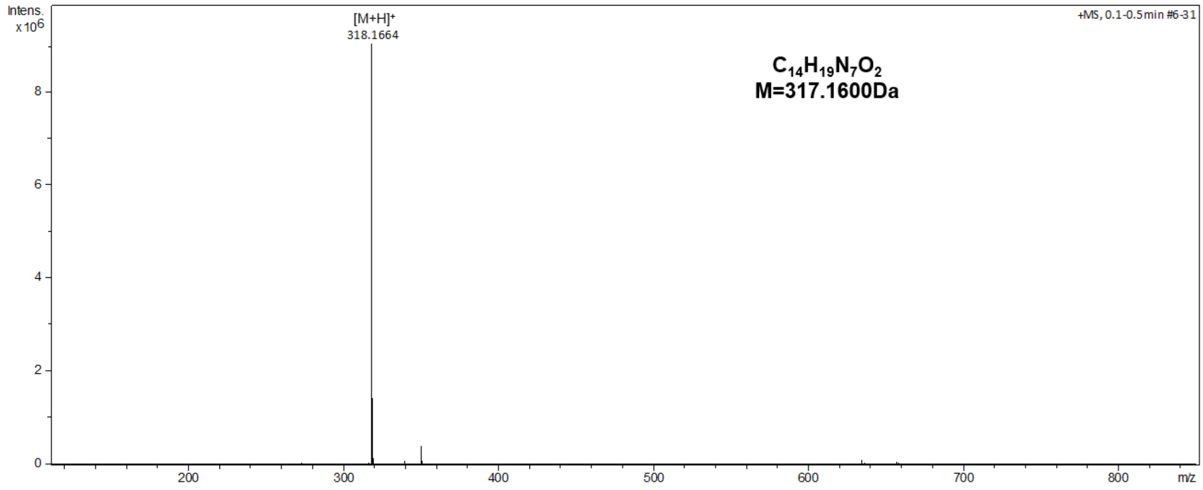

Figure S16. HR-ESI-MS spectrum of compound **5j**.

Table S18. Analysis of other quasi-parent molecular ions of compound **5j**.

Other quasi-parent molecular ions of **5j**	Calculated m/z	Found		Mass measurement error (ppm)
		m/z	Relative intensity (%) to base peak	
$[M+Na]^+$	340.1492	340.1487	0.70	-1.47
$[M+CH_3OH+H]^+$	350.1935	350.1935	4.47	0.00
$[2M+H]^+$	635.3273	635.3273	1.15	0.00
$[2M+Na]^+$	657.3093	657.3097	0.62	0.61

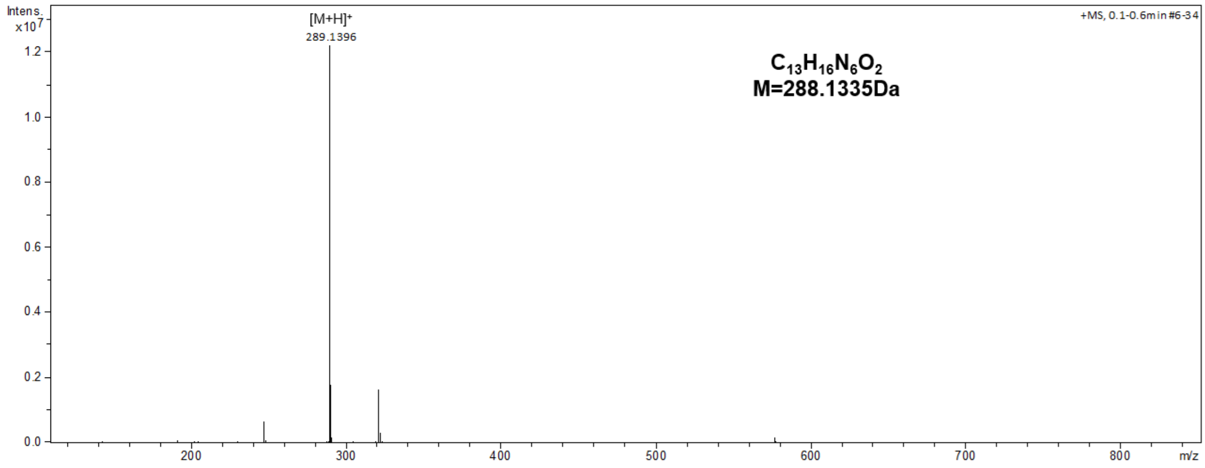

Figure S17. HR-ESI-MS spectrum of compound **5k**.

Table S19. Analysis of other quasi-parent molecular ions of compound **5k**.

Other quasi-parent molecular ions of **5k**	Calculated m/z	Found		Mass measurement error (ppm)
		m/z	Relative intensity (%) to base peak	
$[M+CH_3OH+H]^+$	321.1670	321.1667	13.32	-0.93
$[2M+H]^+$	577.2742	577.2742	1.03	0.00

Table S20. Analysis of fragmentation ion of compound **5k**.

Fragmentation ion of **5k**	Calculated m/z	Found		Mass measurement error (ppm)
		m/z	Relative intensity (%) to base peak	
$[C_{10}H_{11}N_6O_2]^+$	247.0938	247.0937	5.30	-0.40

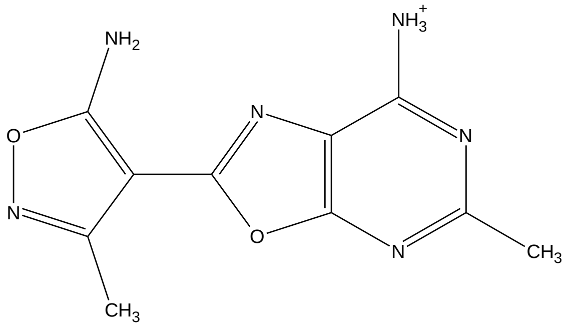

Figure S18. Structure of $[C_{10}H_{11}N_6O_2]^+$ ion from HR-ESI-MS spectrum of compound **5k**.

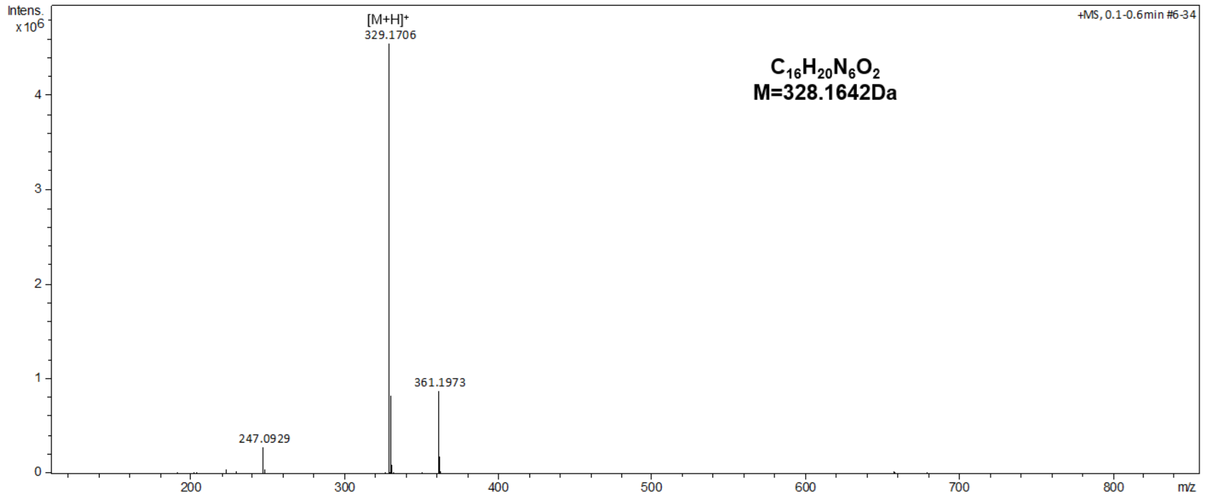

Figure S19. HR-ESI-MS spectrum of compound **5l**.

Table S21. Analysis of other quasi-parent molecular ions of compound **5l**.

Other quasi-parent molecular ions of **5l**	Calculated m/z	Found		Mass measurement error (ppm)
		m/z	Relative intensity (%) to base peak	
$[M+CH_3OH+H]^+$	361.1983	361.1973	19.26	-2.77
$[2M+H]^+$	657.3368	657.3349	0.46	-2.89

Table S22. Analysis of fragmentation ion of compound **5l**.

Fragmentation ion of **5l**	Calculated m/z	Found		Mass measurement error (ppm)
		m/z	Relative intensity (%) to base peak	
$[C_{10}H_{11}N_6O_2]^+$	247.0938	247.0929	6.04	-3.64

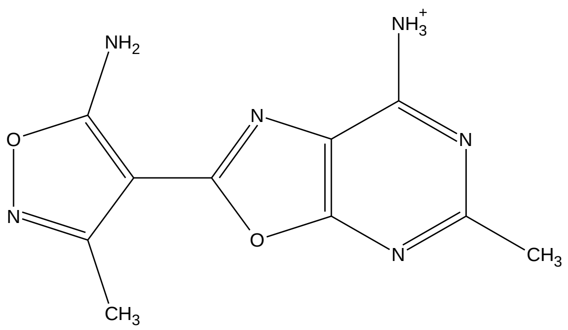

Figure S20. Structure of $[C_{10}H_{11}N_6O_2]^+$ ion from HR-ESI-MS spectrum of compound **5l**.

Analysis and Visualizations of Electrospray Mass Ionization (ESI) spectra of compound 7m

Table S23. Comparative set of masses of quasi-parent molecular $[2M+Na]^+$ ion (as base peak) of compound **7m**. Errors associated with mass measurements of compound **7m**.

No.	Calculated m/z for $[2M+Na]^+$ ion	Found m/z for base peaks (percentage relative intensity)	Mass measurement error (ppm)
**7m**	627.2875	627.2885 (100%)	1.59

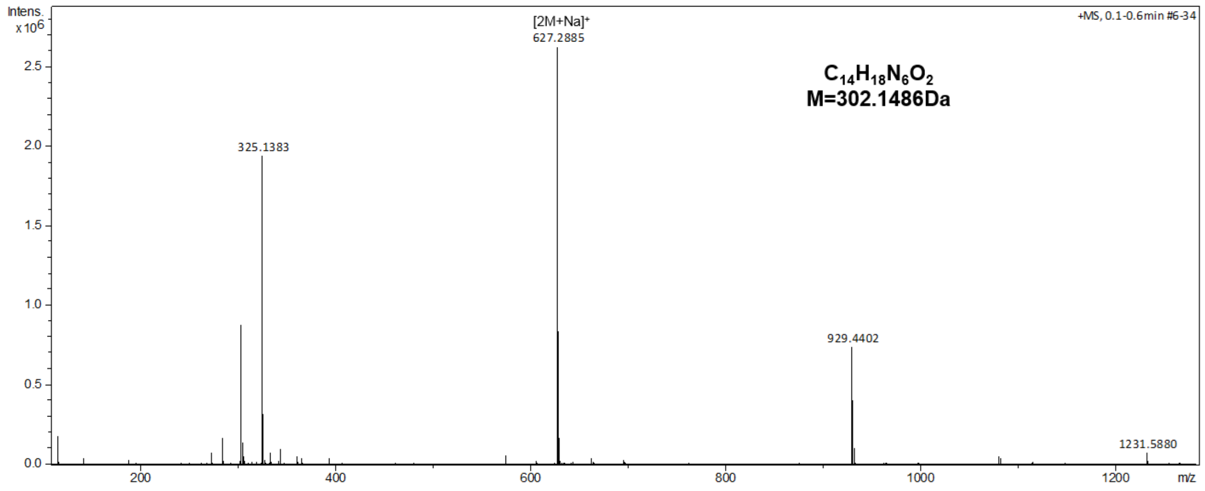

Figure S21. HR-ESI-MS spectrum of compound **7m**.

Table S24. Analysis of other quasi-parent molecular ions of compound **7m**.

Other quasi-parent molecular ions of **7m**	Calculated m/z	Found		Mass measurement error (ppm)
		m/z	Relative intensity (%) to base peak	
$[M+H]^+$	303.1564	303.1565	33.35	0.33
$[M+Na]^+$	325.1383	325.1383	73.80	0.00
$[M+H_2O+Na]^+$	343.1489	343.1482	3.67	-2.04
$[3M+Na]^+$	929.4366	929.4402	28.05	3.87

Visualizations of Nuclear magnetic resonance (NMR) and Infrared (IR) spectra of compound 2

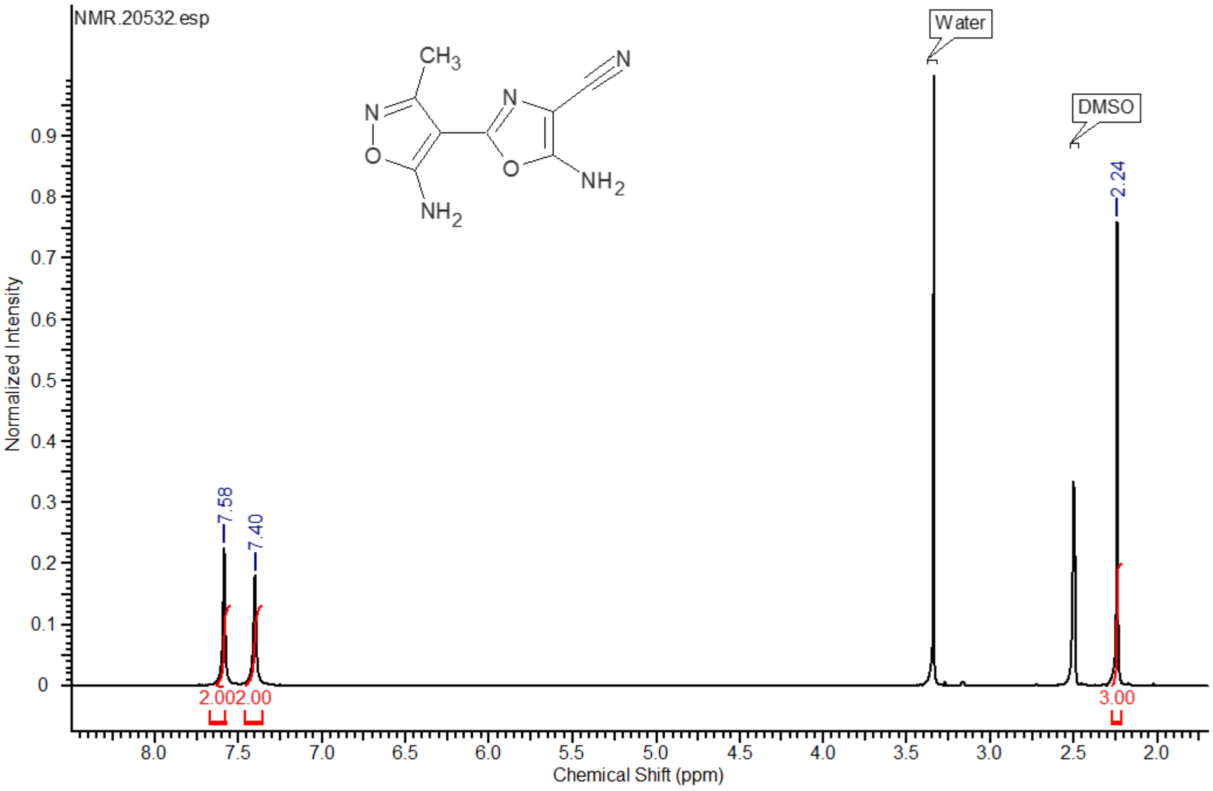

Figure S22. ^{1}H-NMR spectrum of compound **2** in DMSO-d6.

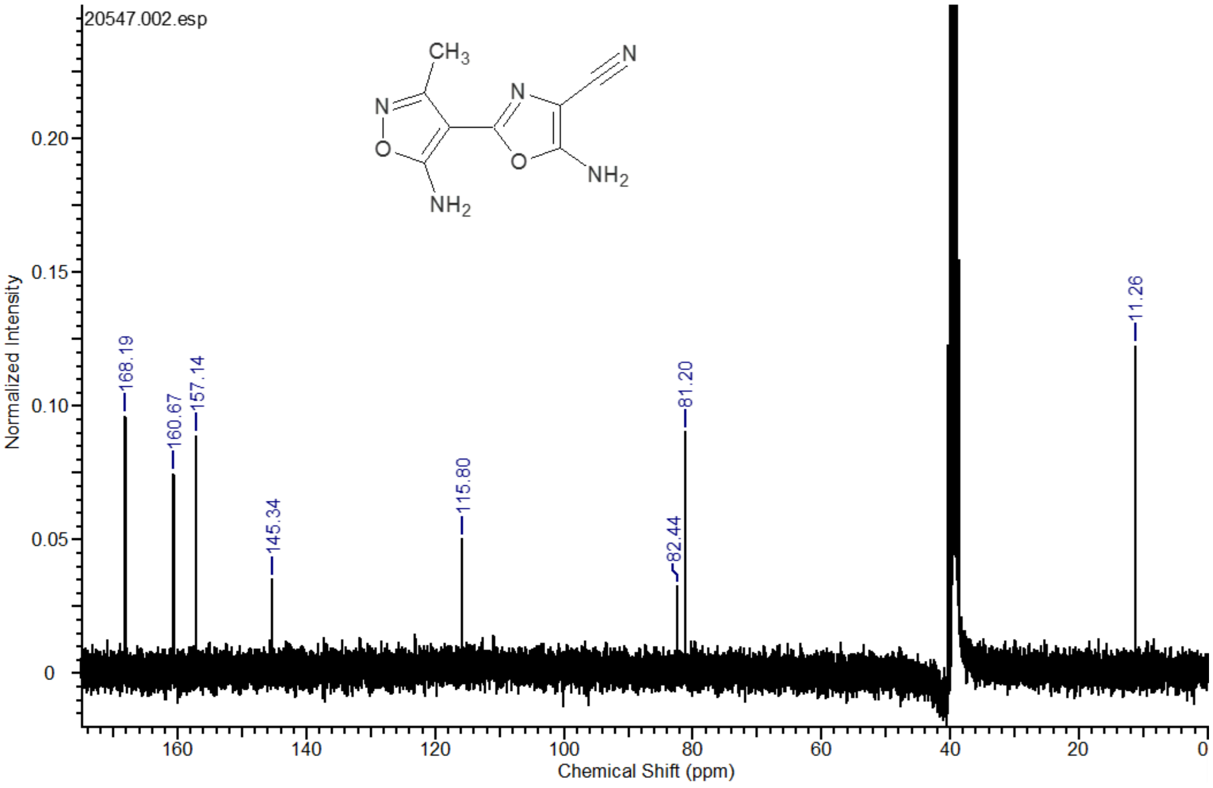

Figure S23. ^{13}C-NMR spectrum of compound **2** in DMSO-d6.

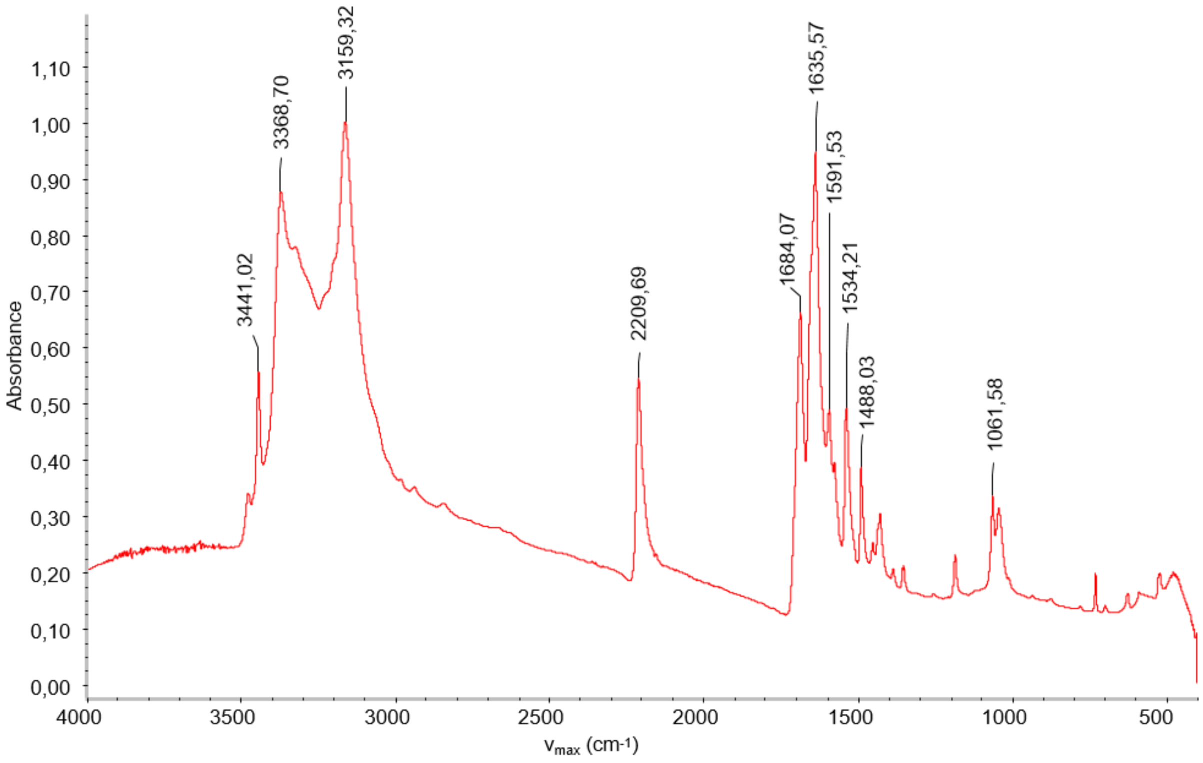

Figure S24. ATR-FTIR spectrum of compound **2**.

Visualizations of Nuclear magnetic resonance (NMR) and Infrared (IR) spectra of compound 3

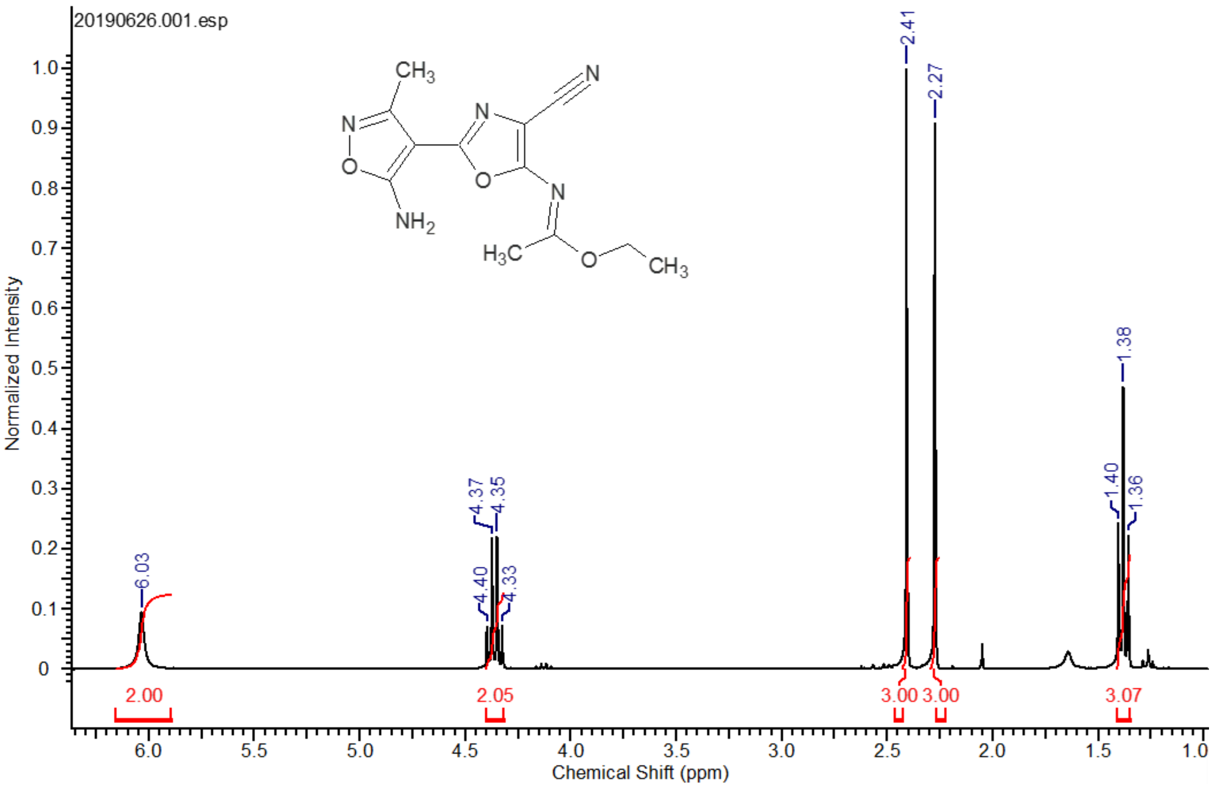

Figure S25. ^{1}H-NMR spectrum of compound **3** in $CDCl_3$.

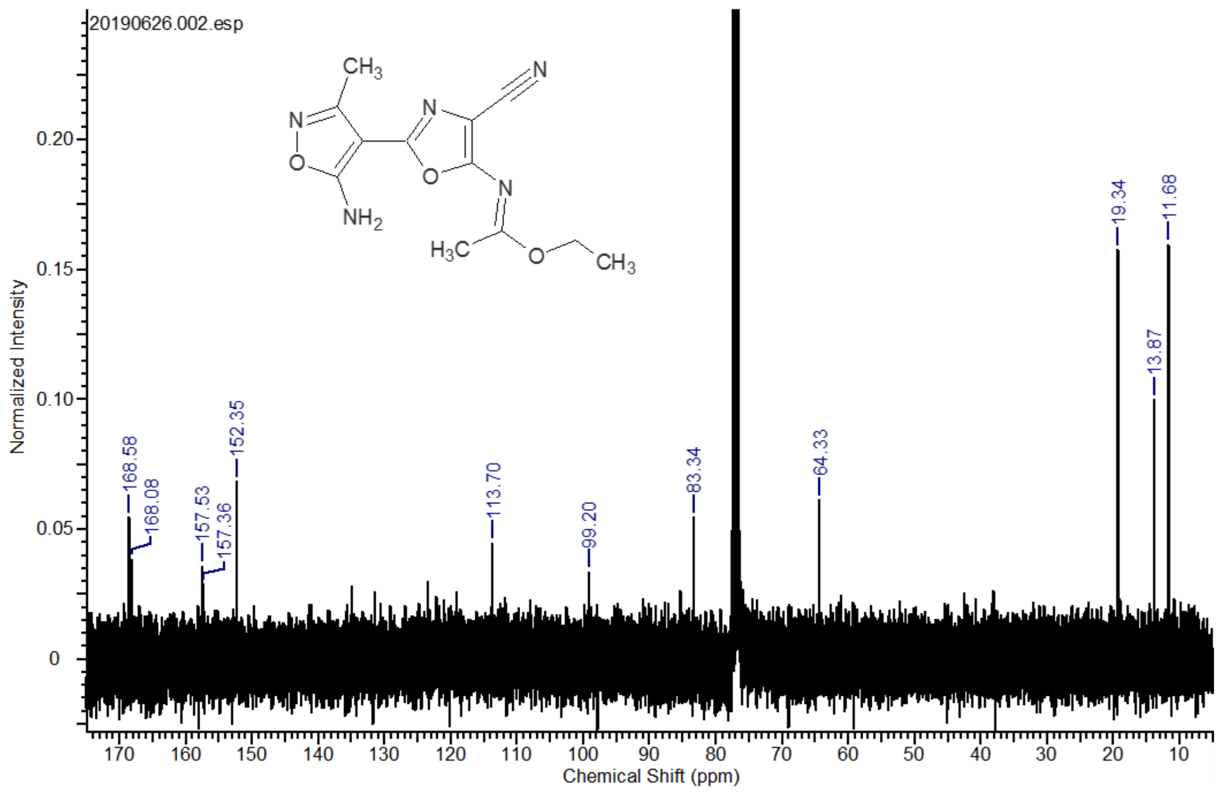

Figure S26. ^{13}C-NMR spectrum of compound **3** in $CDCl_3$.

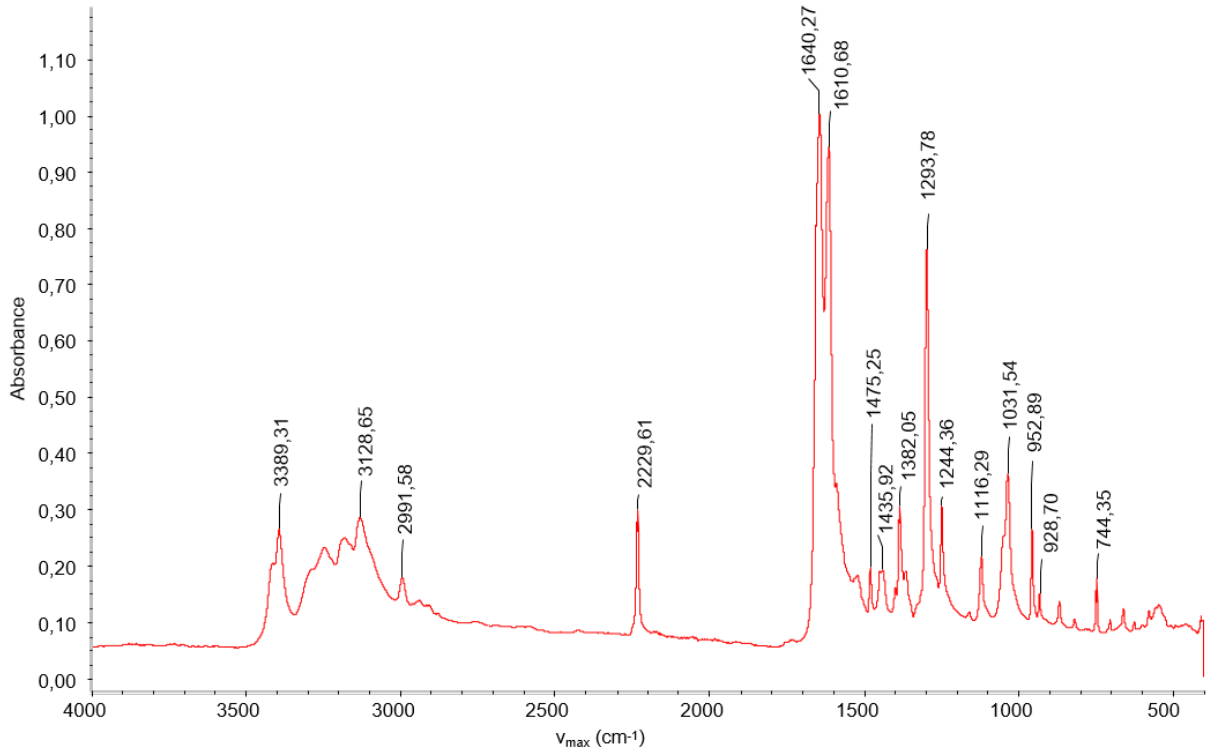

Figure S27. ATR-FTIR spectrum of compound **3**.

Visualizations of Nuclear magnetic resonance (NMR) and Infrared (IR) spectra of compound 4

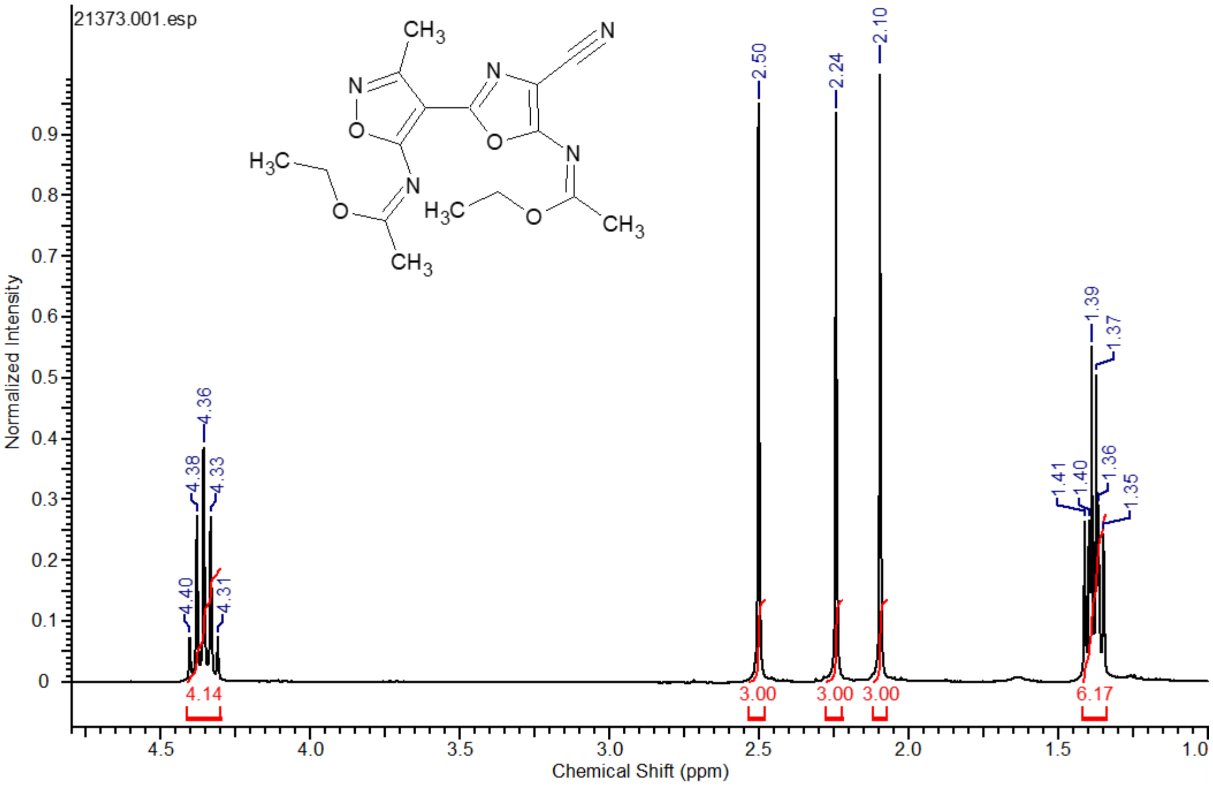

Figure S28. ^{1}H-NMR spectrum of compound **4** in $CDCl_3$.

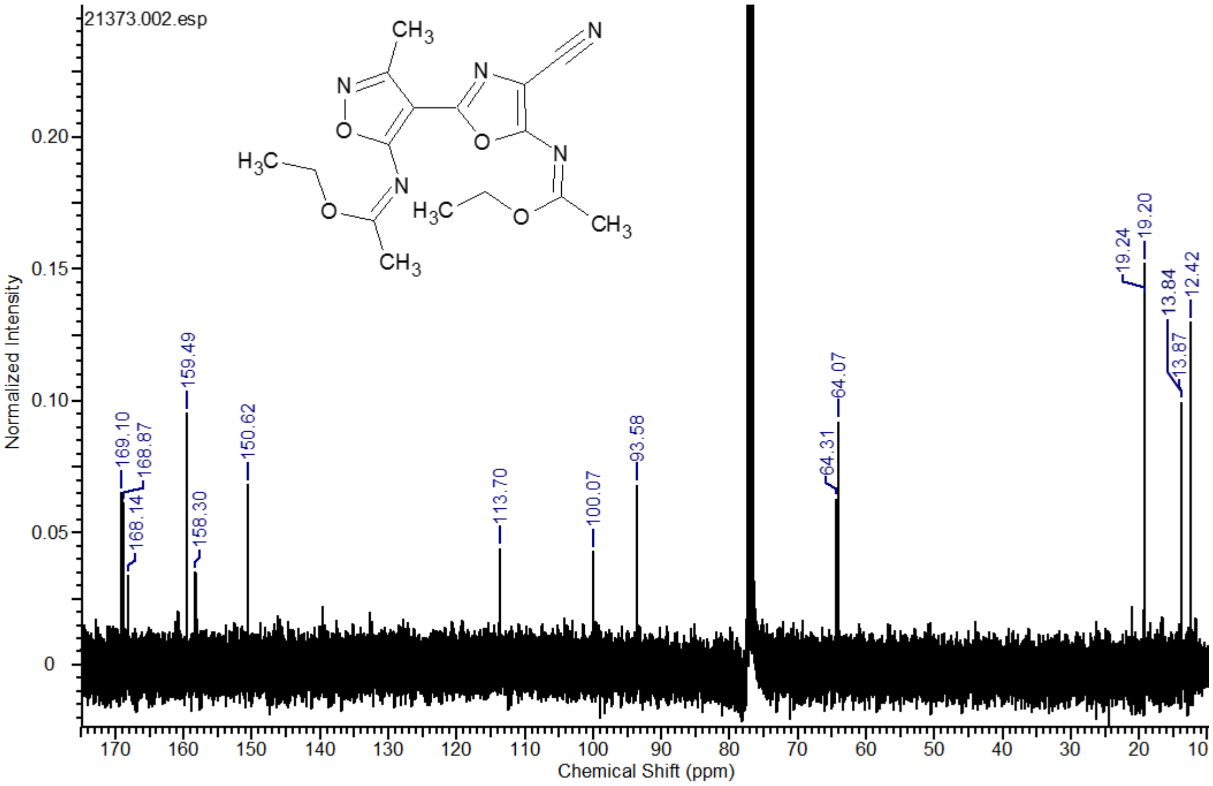

Figure S29. ^{13}C-NMR spectrum of compound **4** in $CDCl_3$.

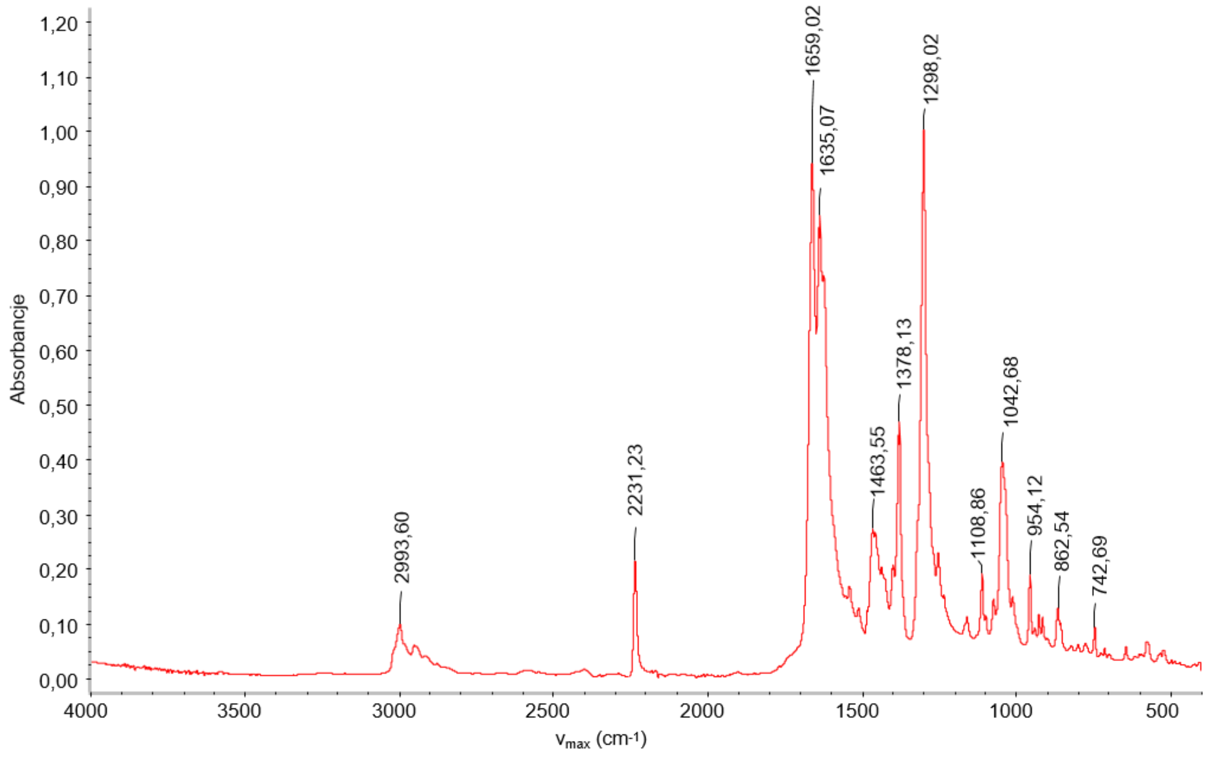

Figure S30. ATR-FTIR spectrum of compound **4**.

Visualizations of Nuclear magnetic resonance (NMR) and Infrared (IR) spectra of compound 5a

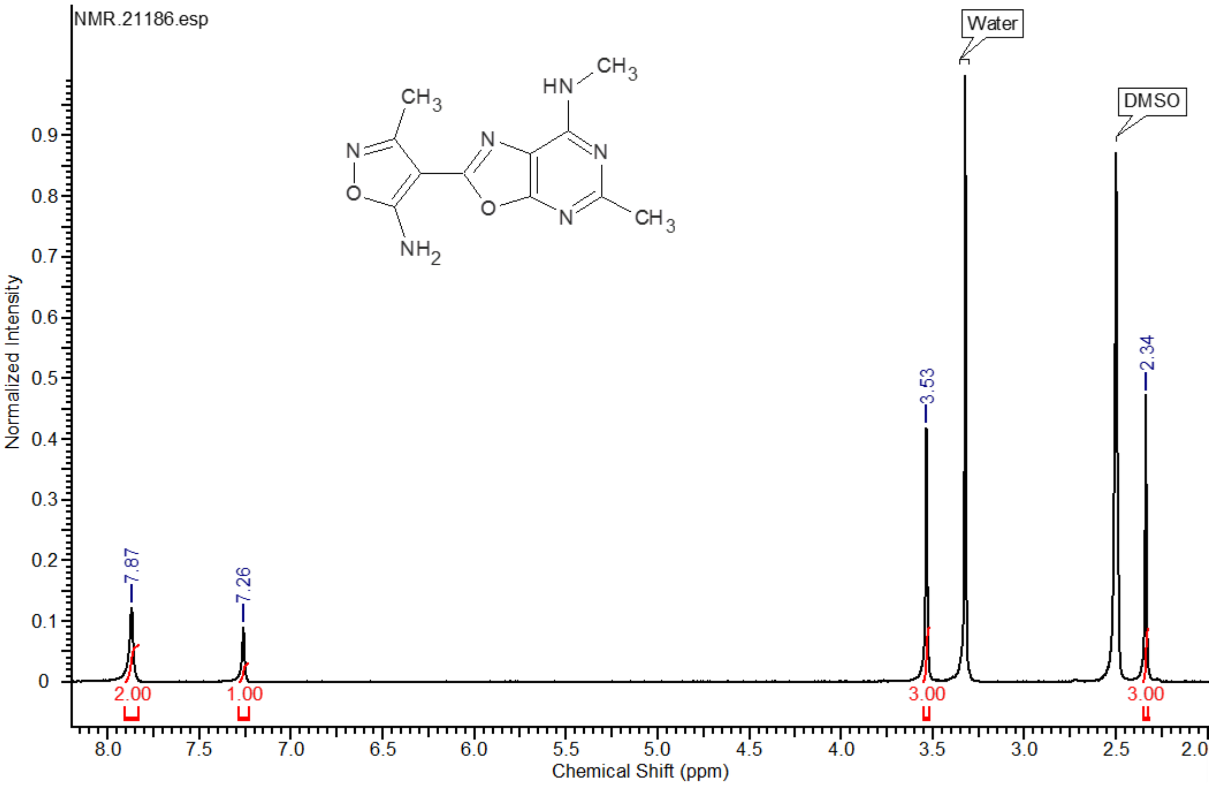

Figure S31. ^{1}H-NMR spectrum of compound **5a** in DMSO-d6.

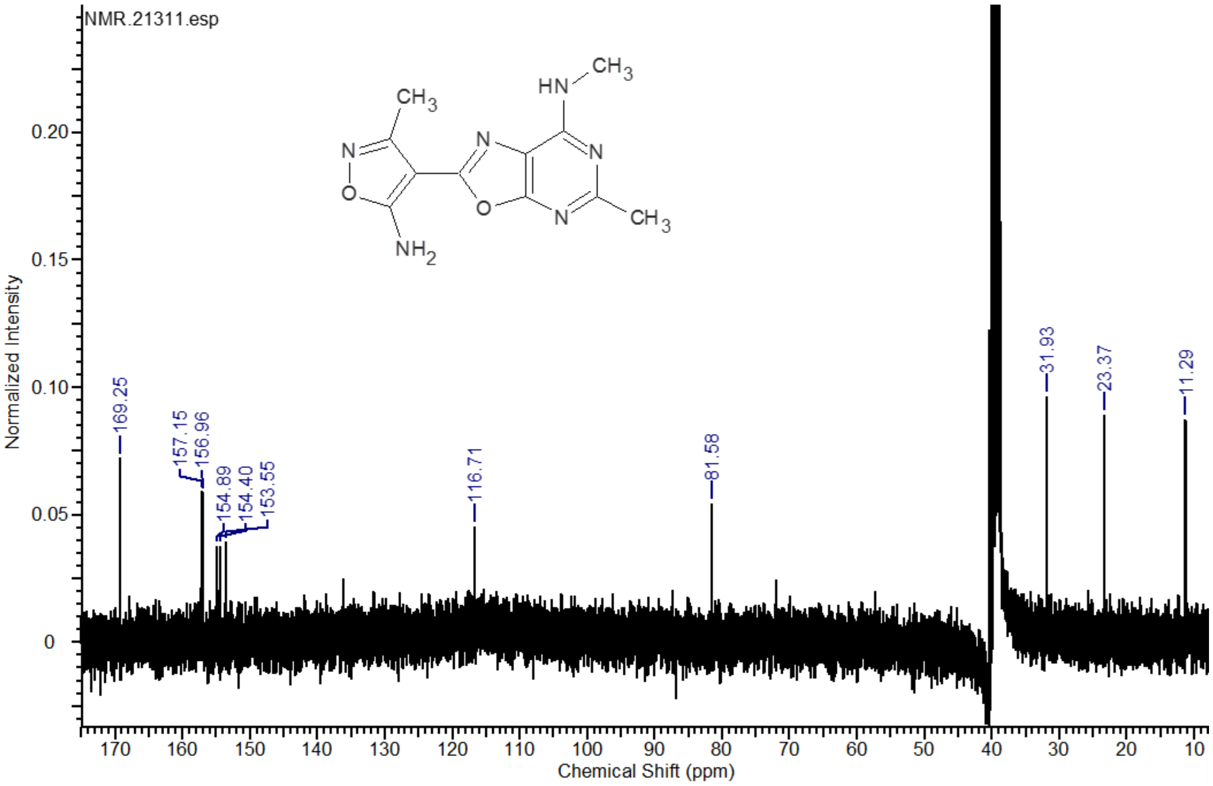

Figure S32. ^{13}C-NMR spectrum of compound **5a** in DMSO-d6.

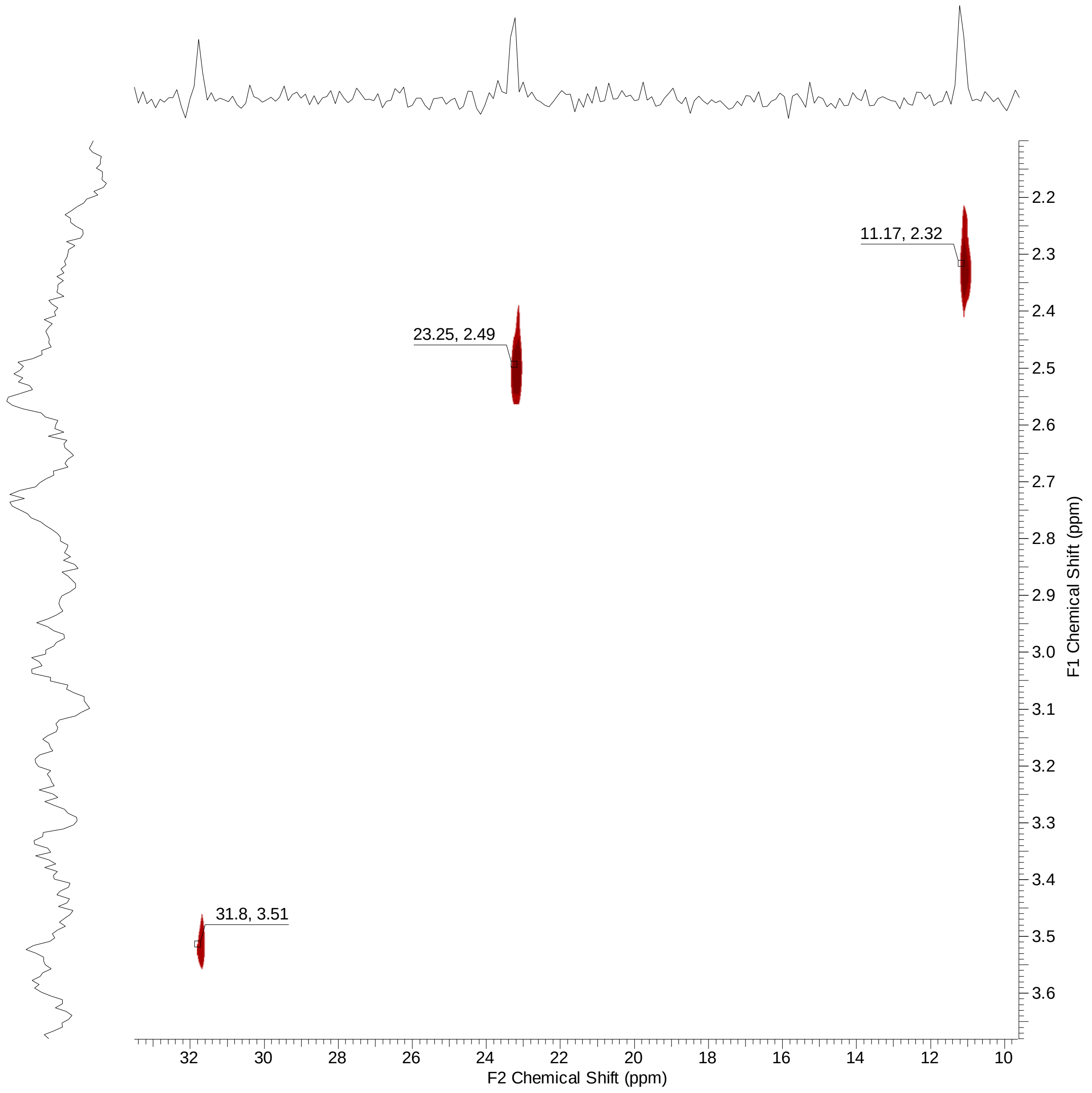

Figure S33. ^{1}H-^{13}C 2D COSY NMR spectrum of compound **5a** in DMSO-d6.

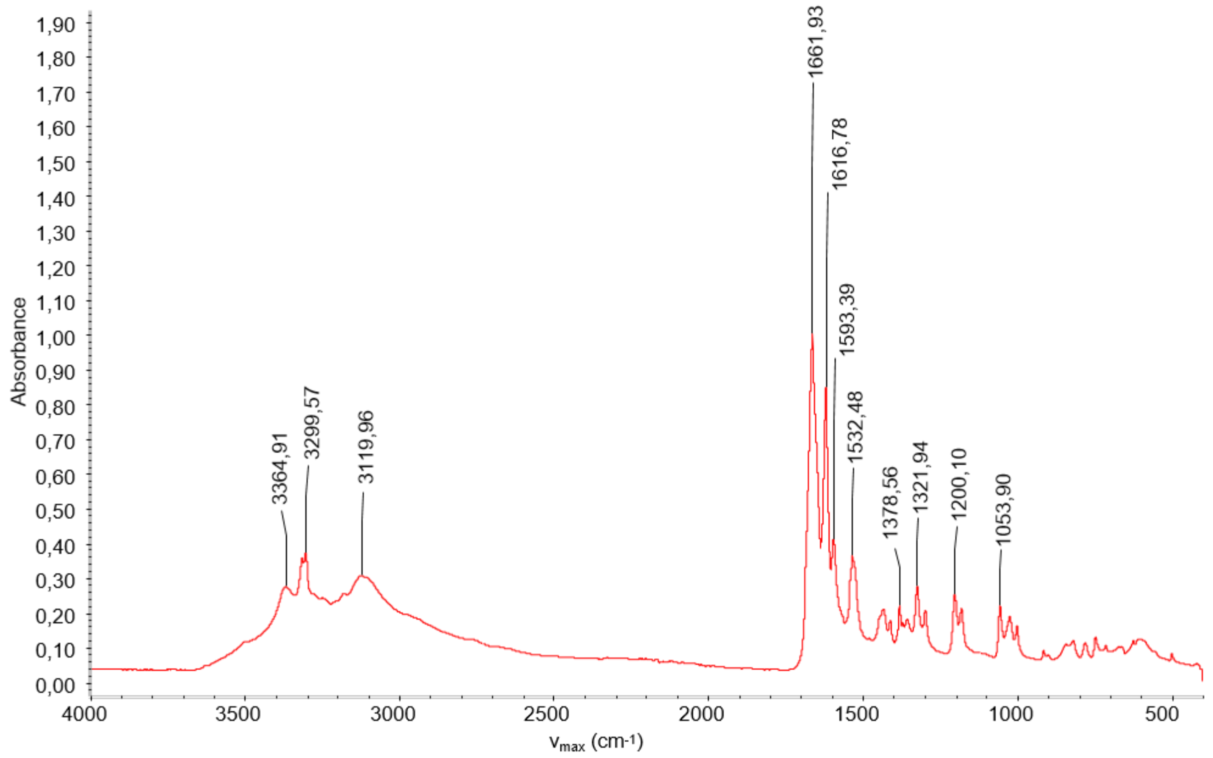

Figure S34. ATR-FTIR spectrum of compound **5a**.

Visualizations of Nuclear magnetic resonance (NMR) and Infrared (IR) spectra of compound 5b

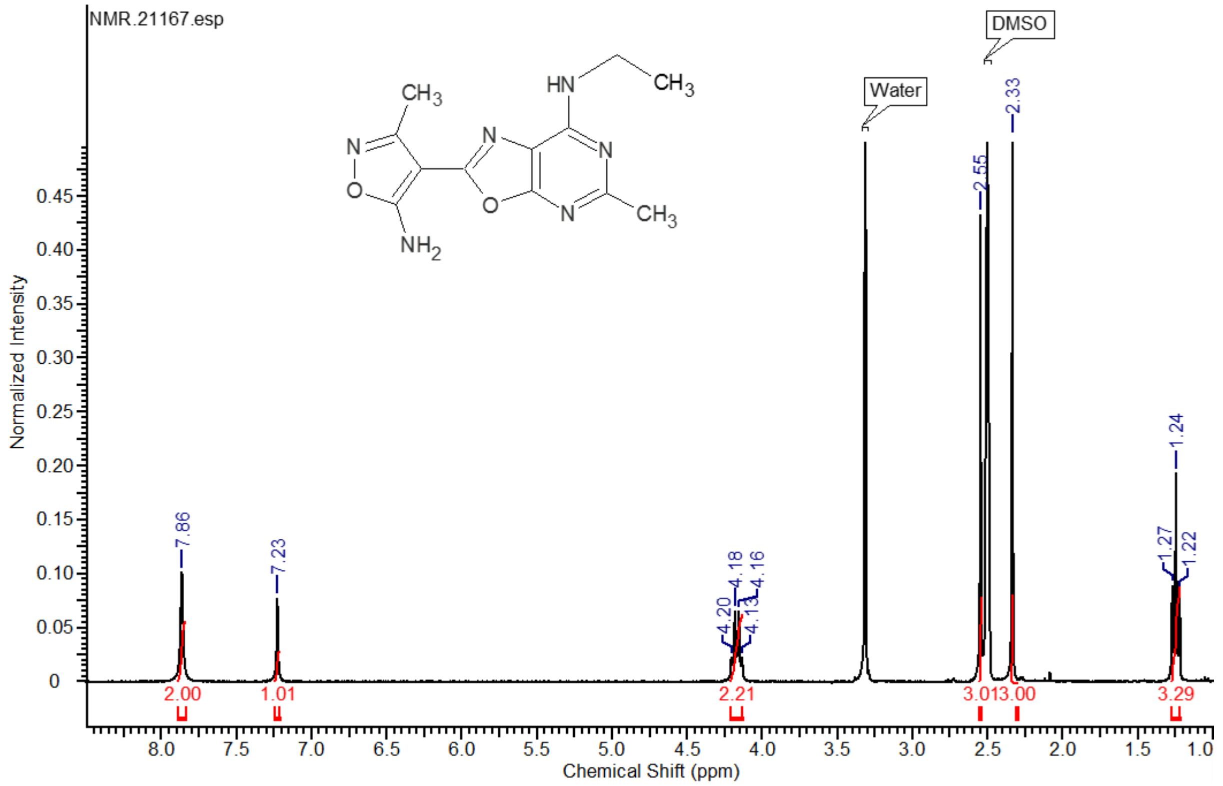

Figure S35. ^{1}H-NMR spectrum of compound **5b** in DMSO-d6.

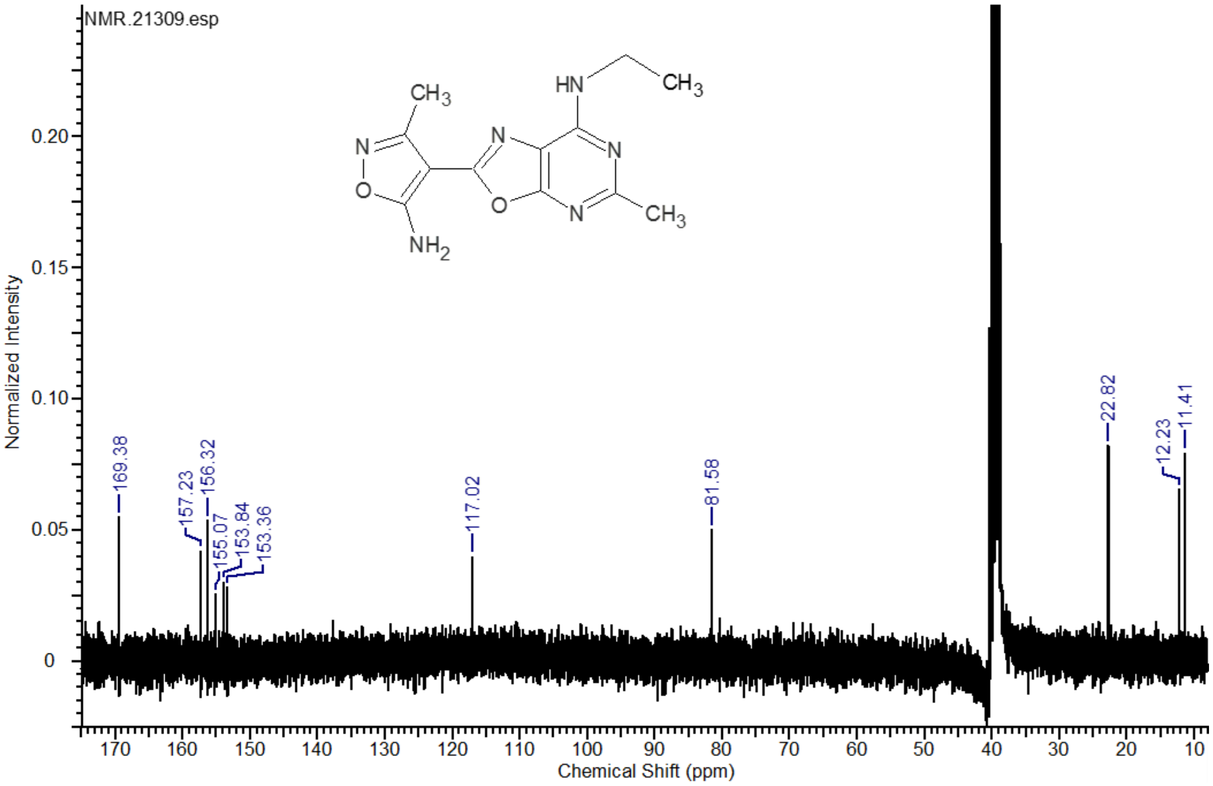

Figure S36. ^{13}C-NMR spectrum of compound **5b** in DMSO-d6.

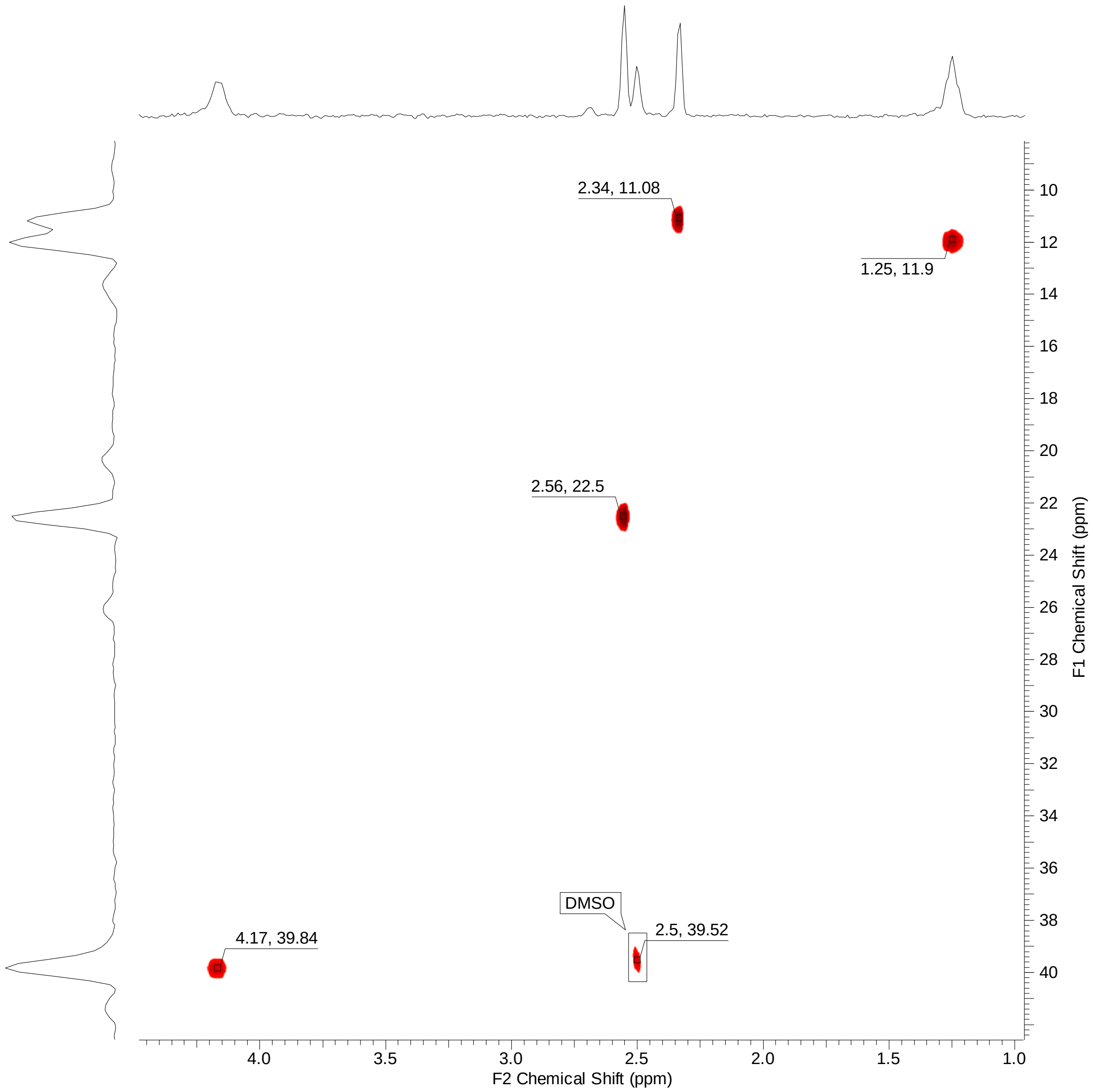

Figure S37. ^{1}H-^{13}C 2D HSQC (Heteronuclear Single Quantum Coherence=Heteronuclear Single Quantum Correlation) NMR of compound **5b** in DMSO-d6.

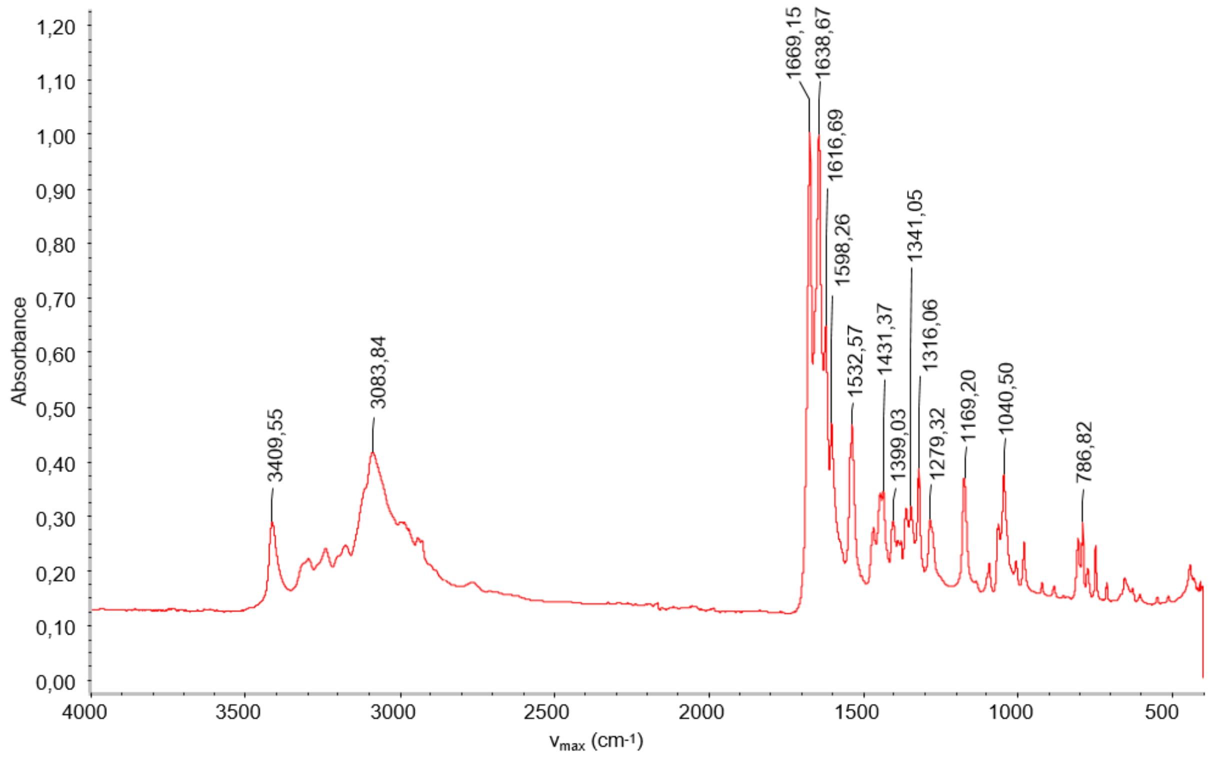

Figure S38. ATR-FTIR spectrum of compound **5b**.

Visualizations of Nuclear magnetic resonance (NMR) and Infrared (IR) spectra of compound 5c

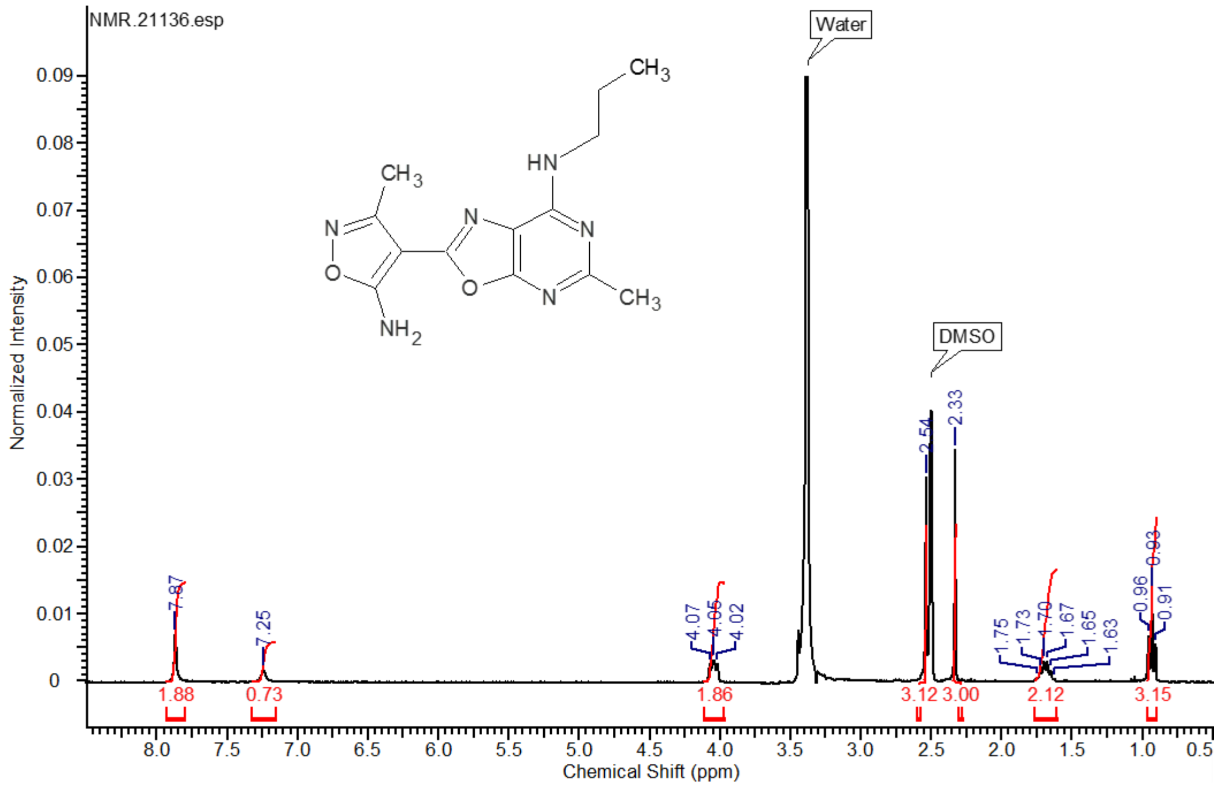

Figure S39. ^{1}H-NMR spectrum of compound **5c** in DMSO-d6 (method A).

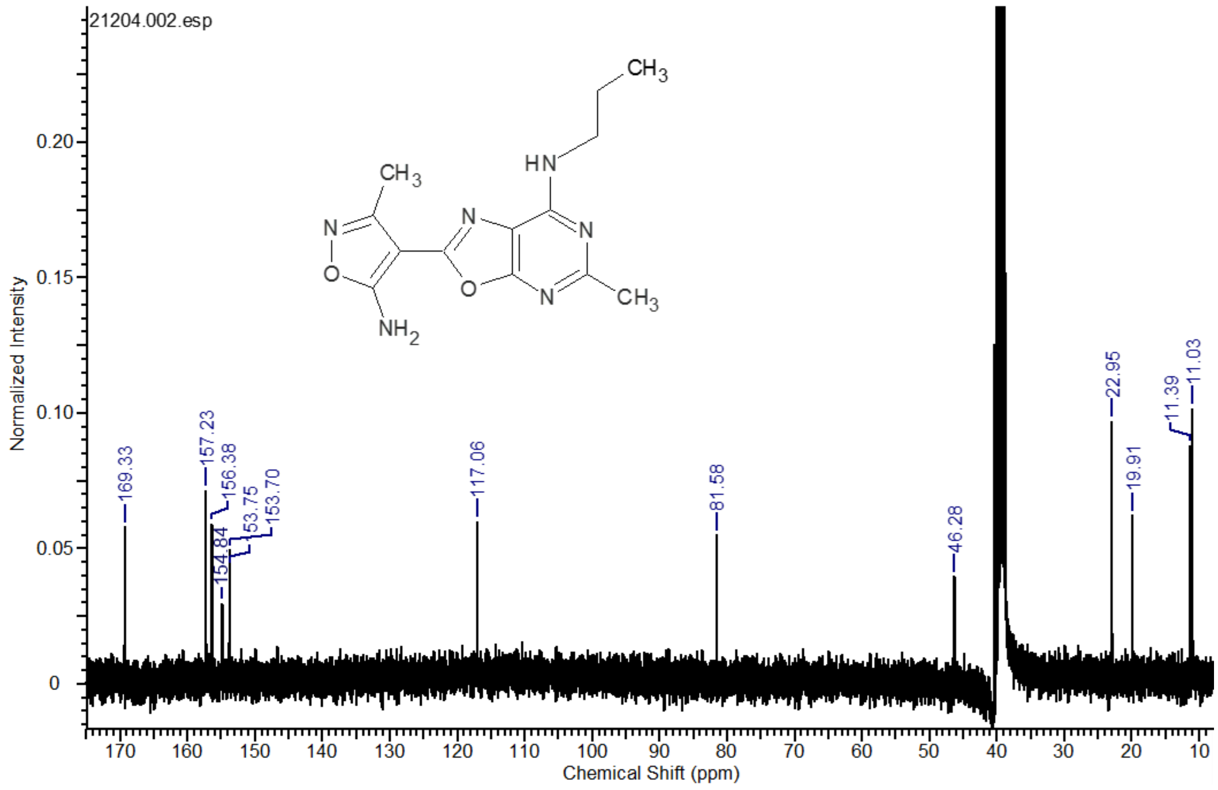

Figure S40. ^{13}C-NMR spectrum of compound **5c** in DMSO-d6 (method A).

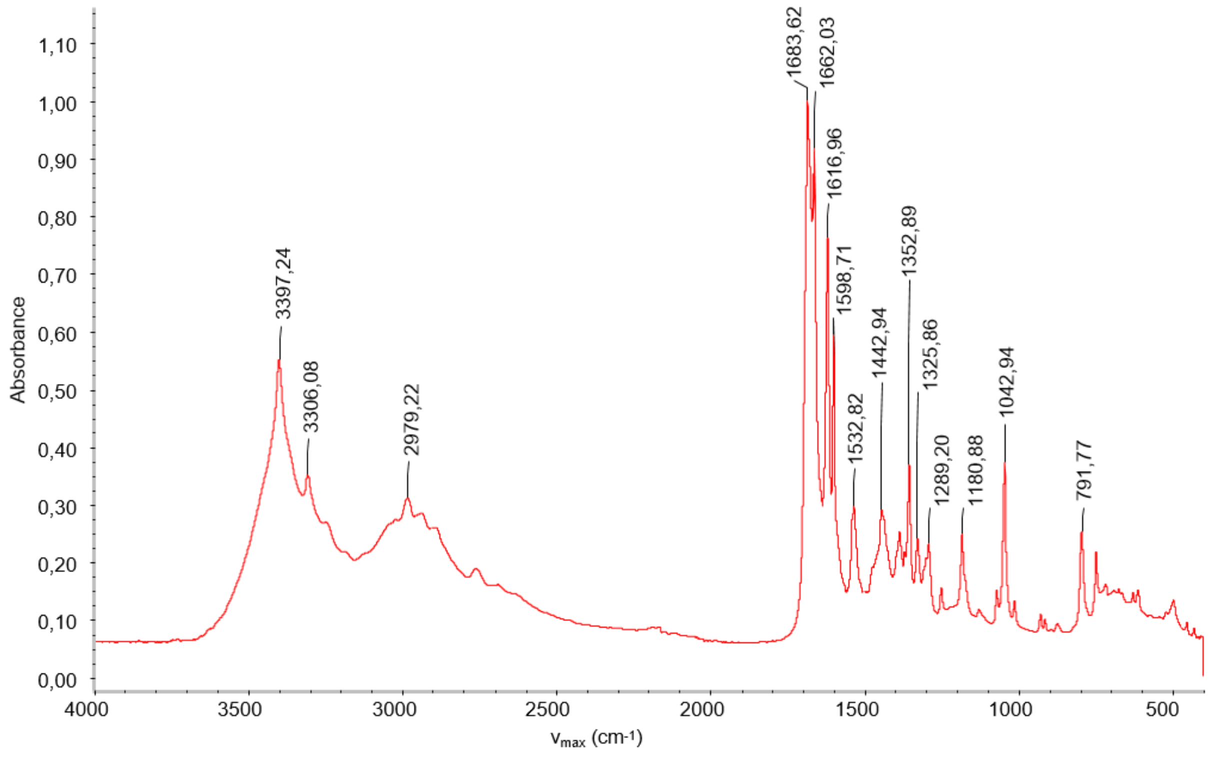

Figure S41. ATR-FTIR spectrum of compound **5c** (method A).

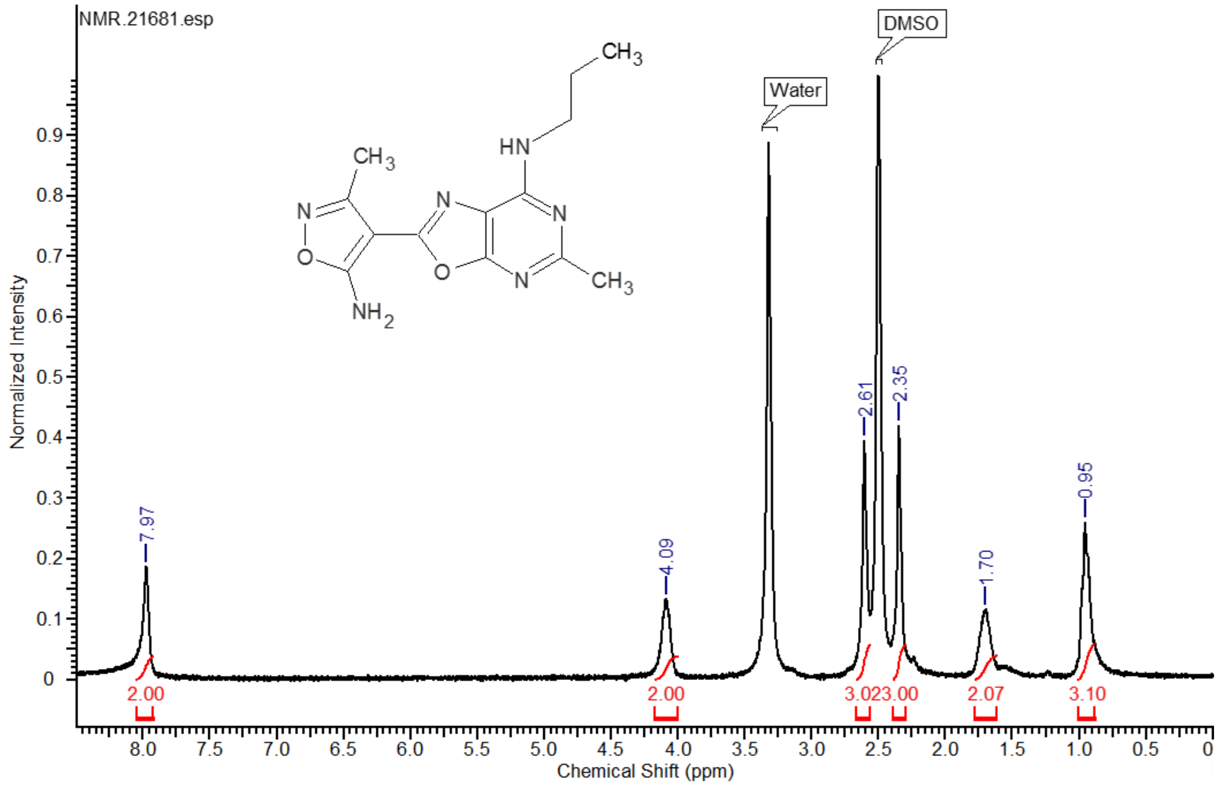

Figure S42. ^{1}H-NMR spectrum of compound **5c** in DMSO-d6 (method B).

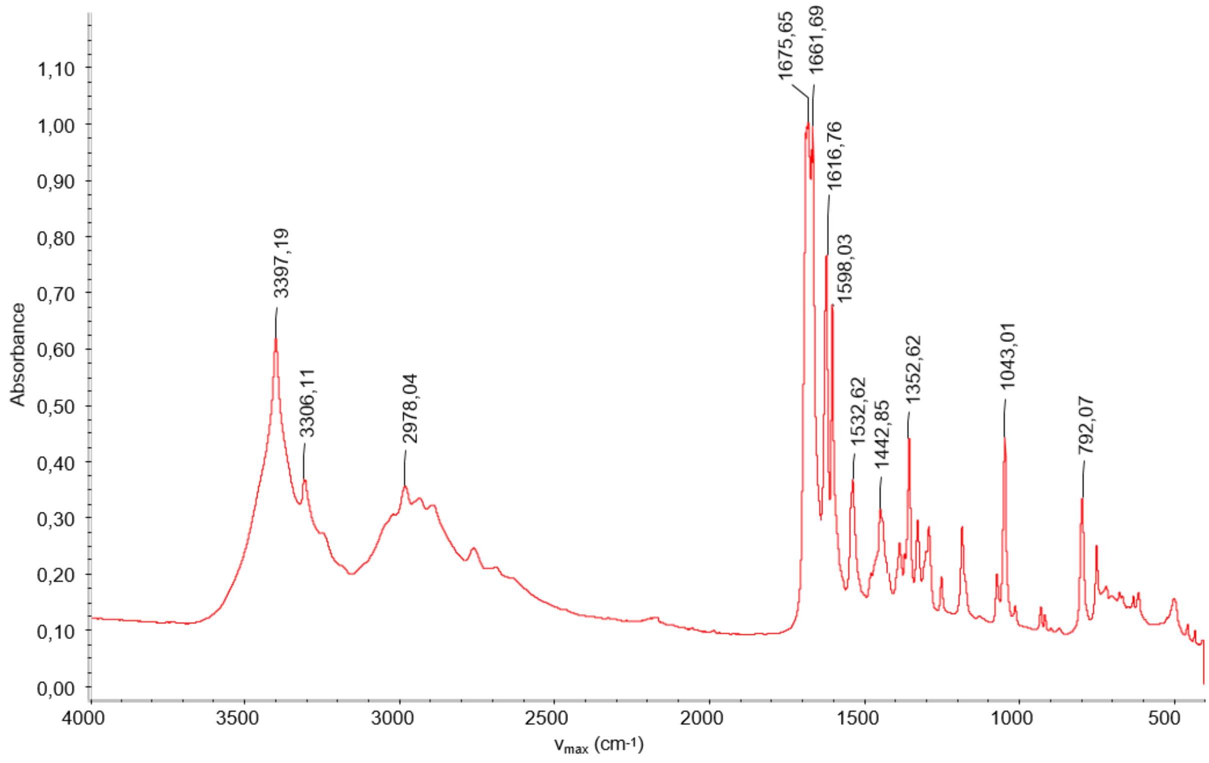

Figure S43. ATR-FTIR spectrum of compound **5c** (method B).

Visualizations of Nuclear magnetic resonance (NMR) and Infrared (IR) spectra of compound 5d

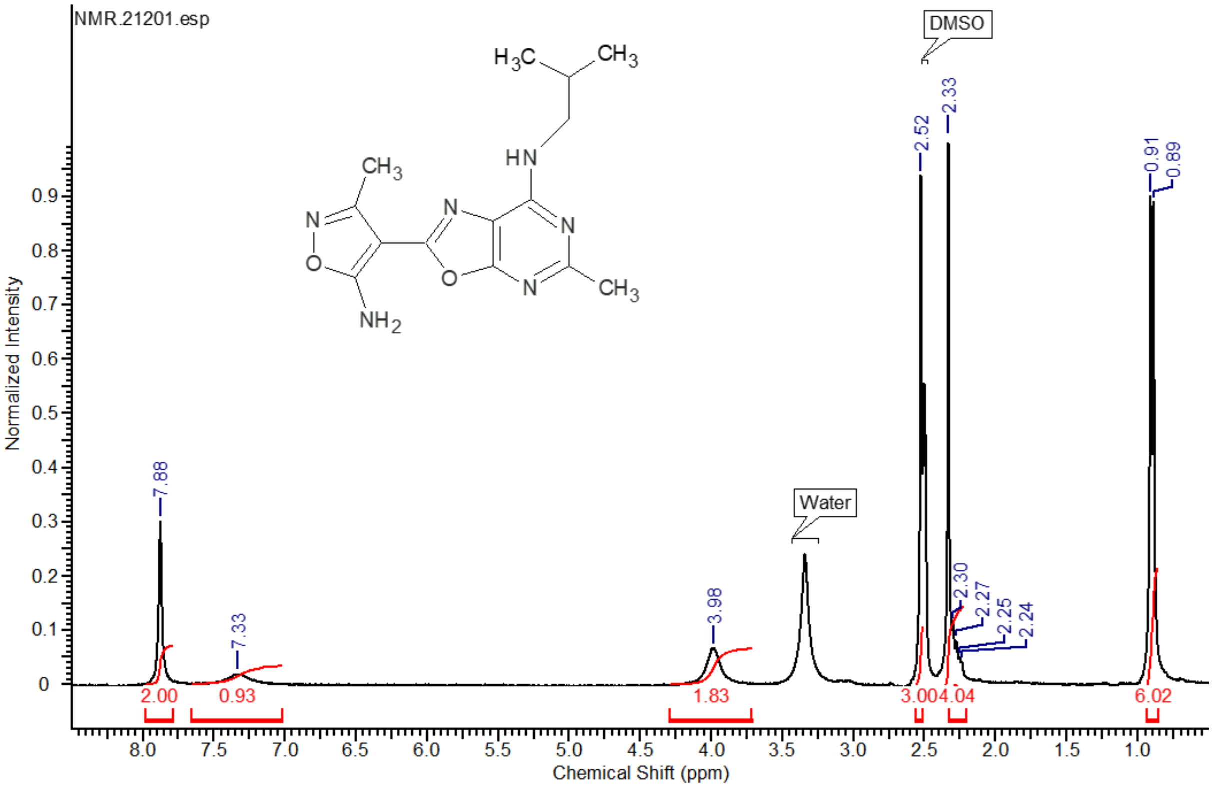

Figure S44. ^{1}H-NMR spectrum of compound **5d** in DMSO-d6.

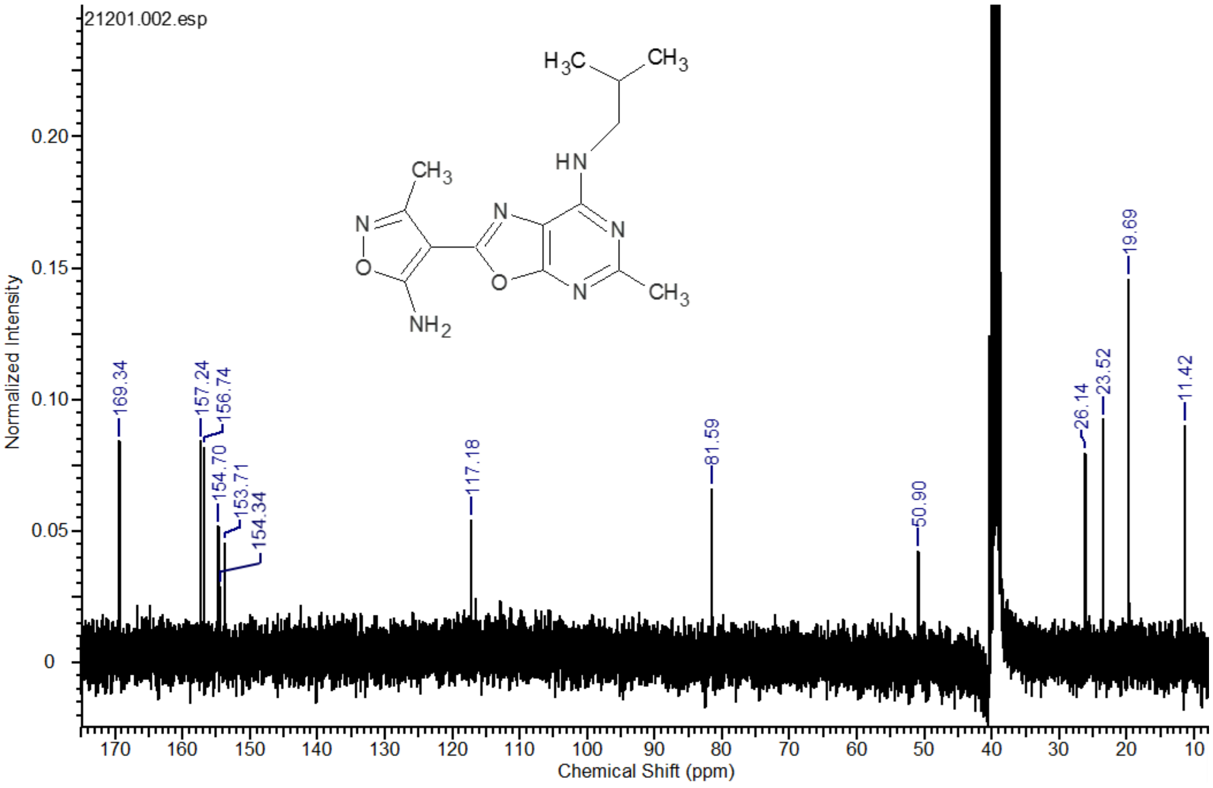

Figure S45. ^{13}C-NMR spectrum of compound **5d**.

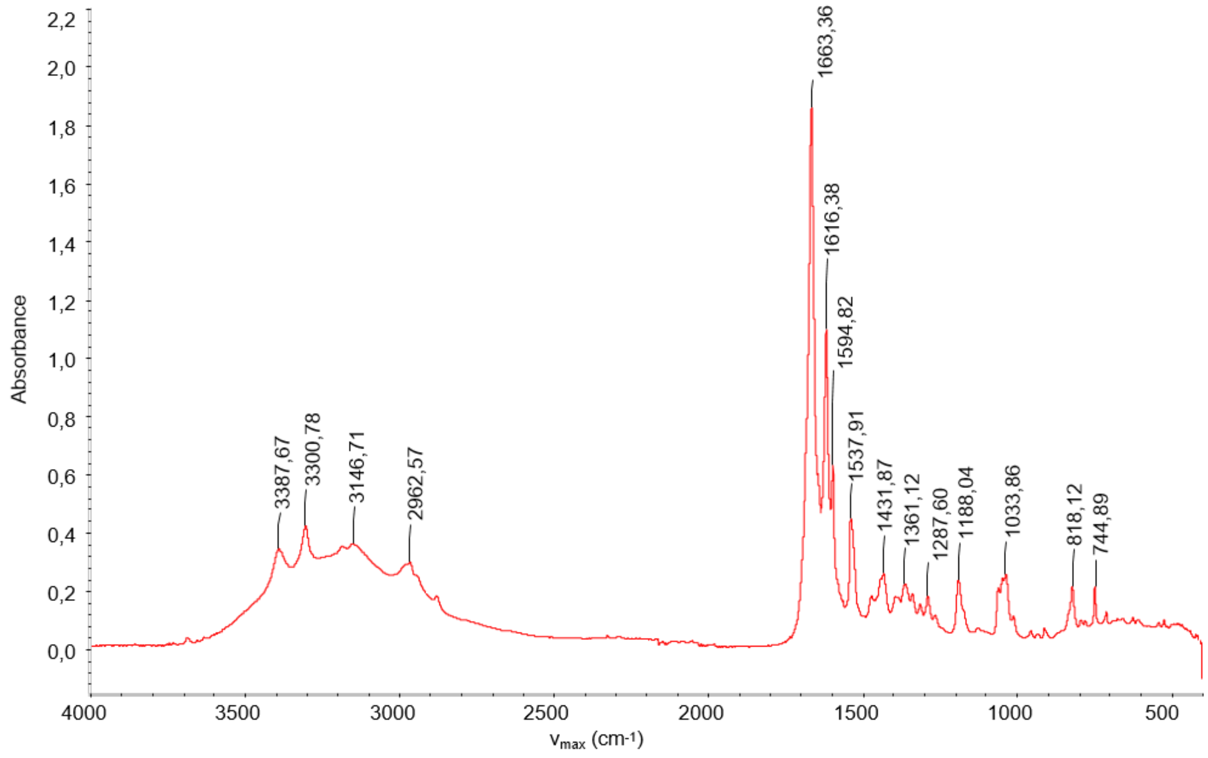

Figure S46. ATR-FTIR spectrum of compound **5d**.

Visualizations of Nuclear magnetic resonance (NMR) and Infrared (IR) spectra of compound 5e

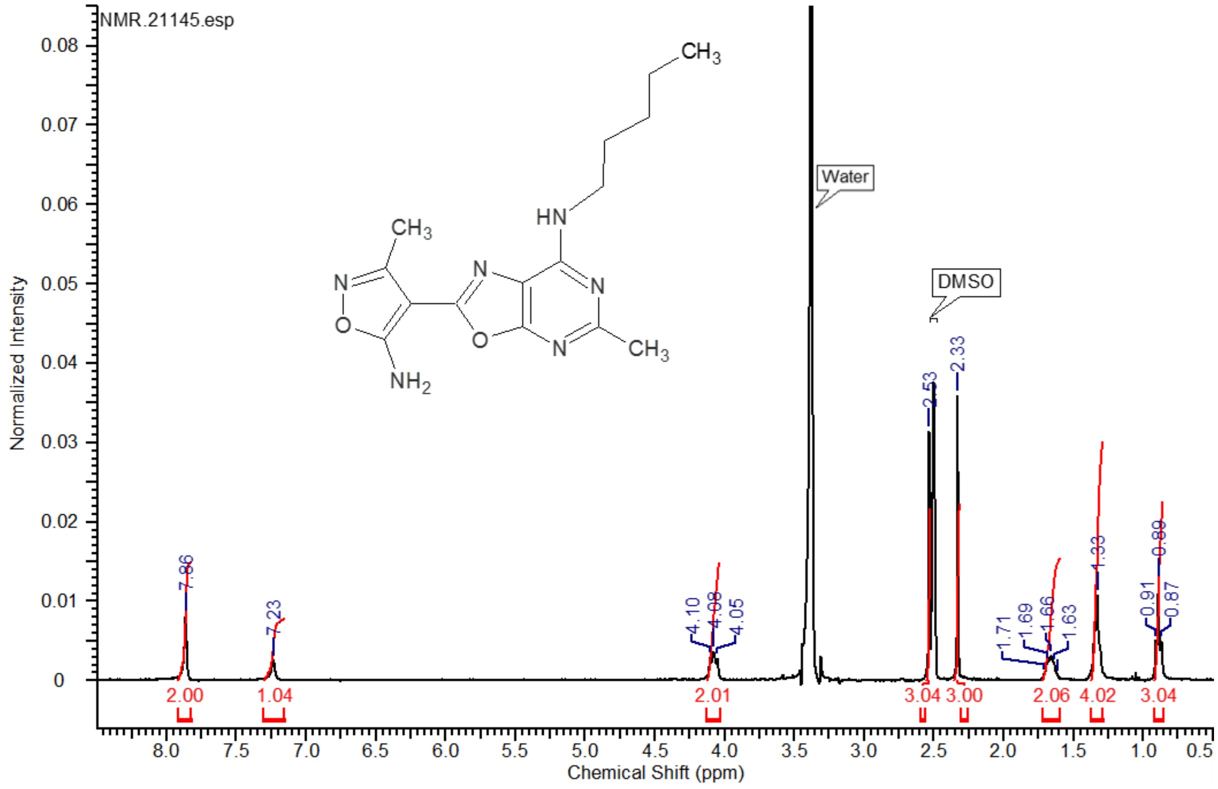

Figure S47. ^{1}H-NMR spectrum of compound **5e** in DMSO-d6.

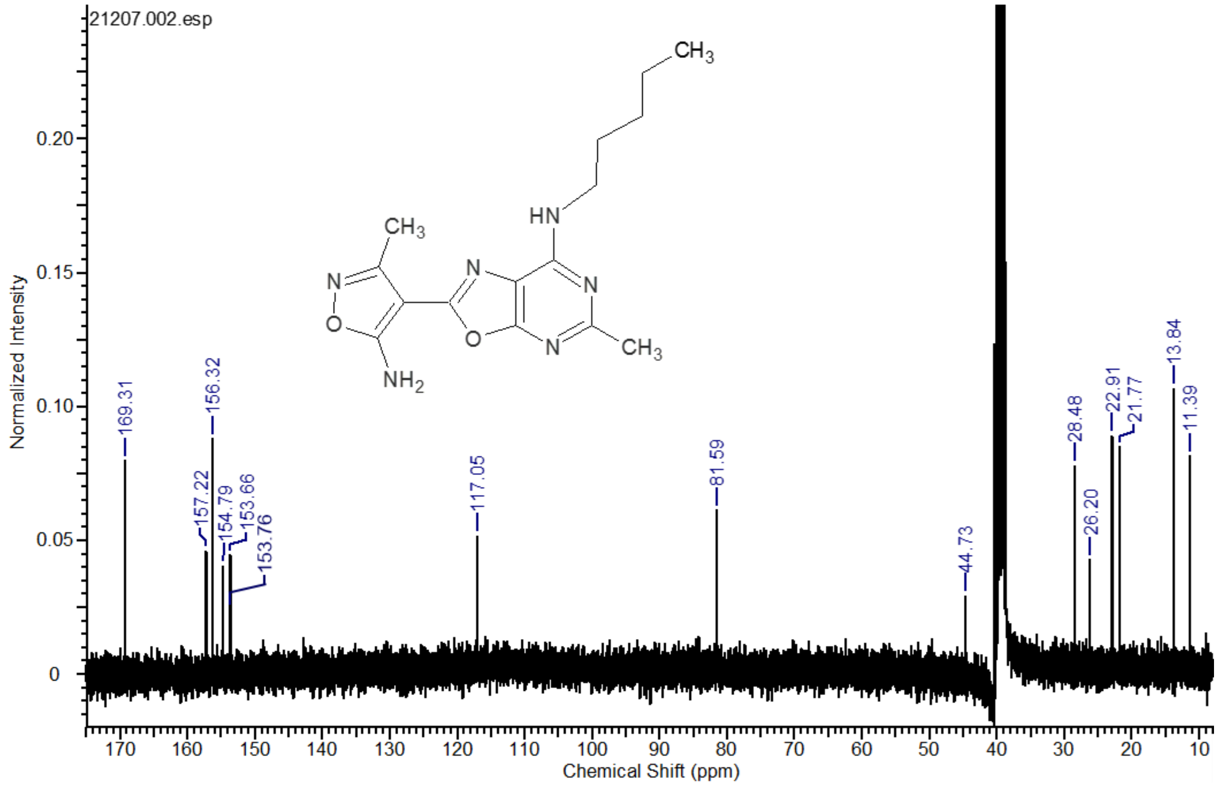

Figure S48. ^{13}C-NMR spectrum of compound **5e** in DMSO-d6.

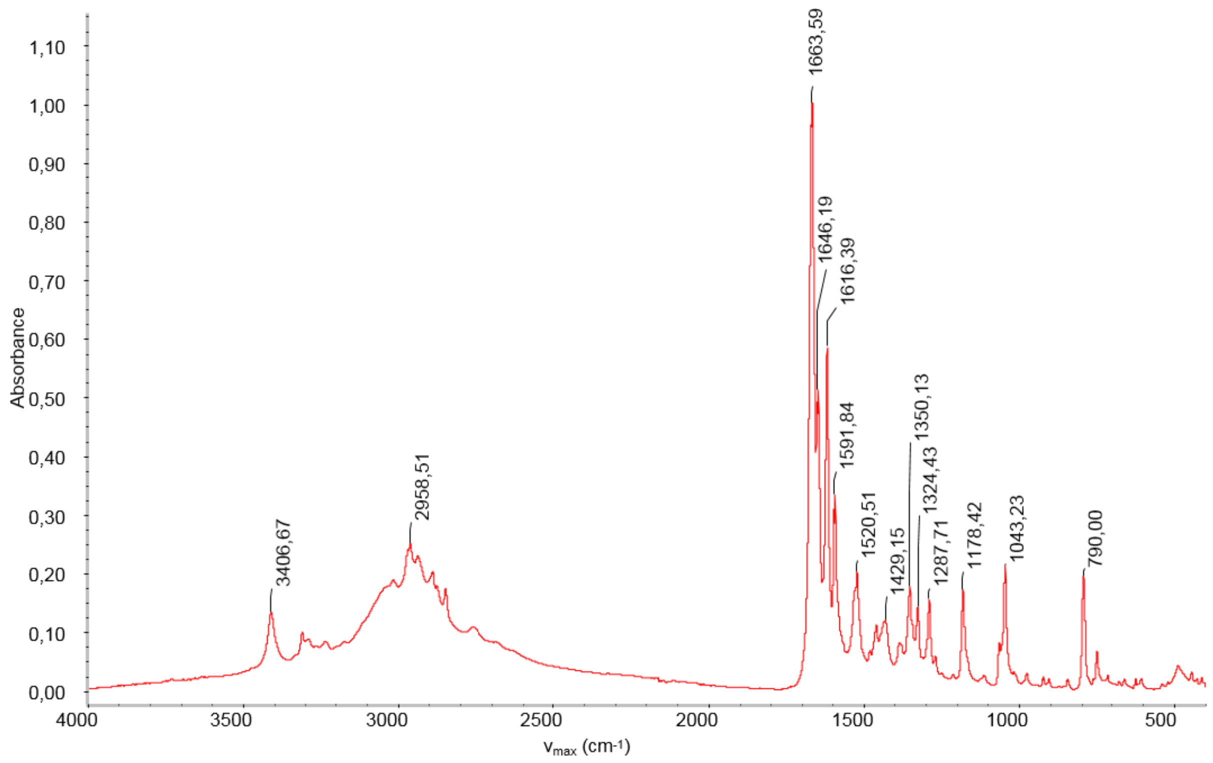

Figure S49. ATR-FTIR spectrum of compound **5e**.

Visualizations of Nuclear magnetic resonance (NMR) and Infrared (IR) spectra of compound 5f

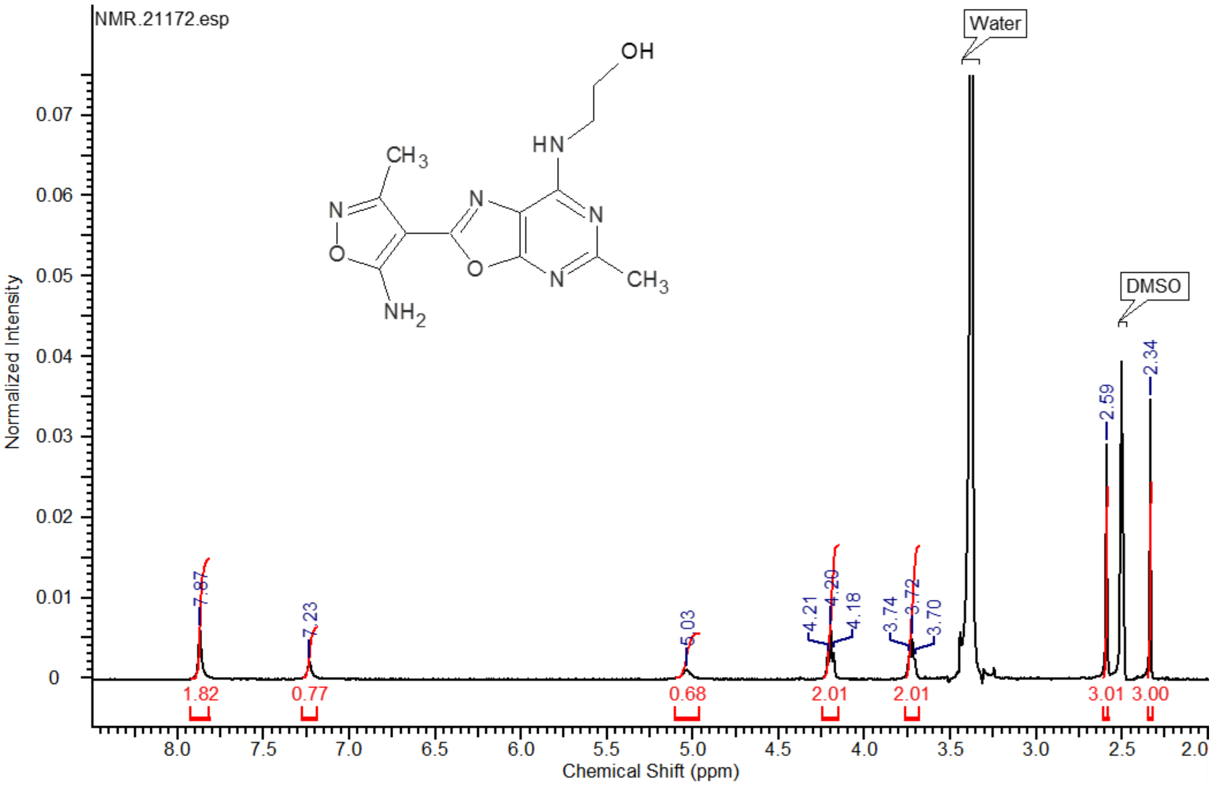

Figure S50. ^{1}H-NMR spectrum of compound **5f** in DMSO-d6.

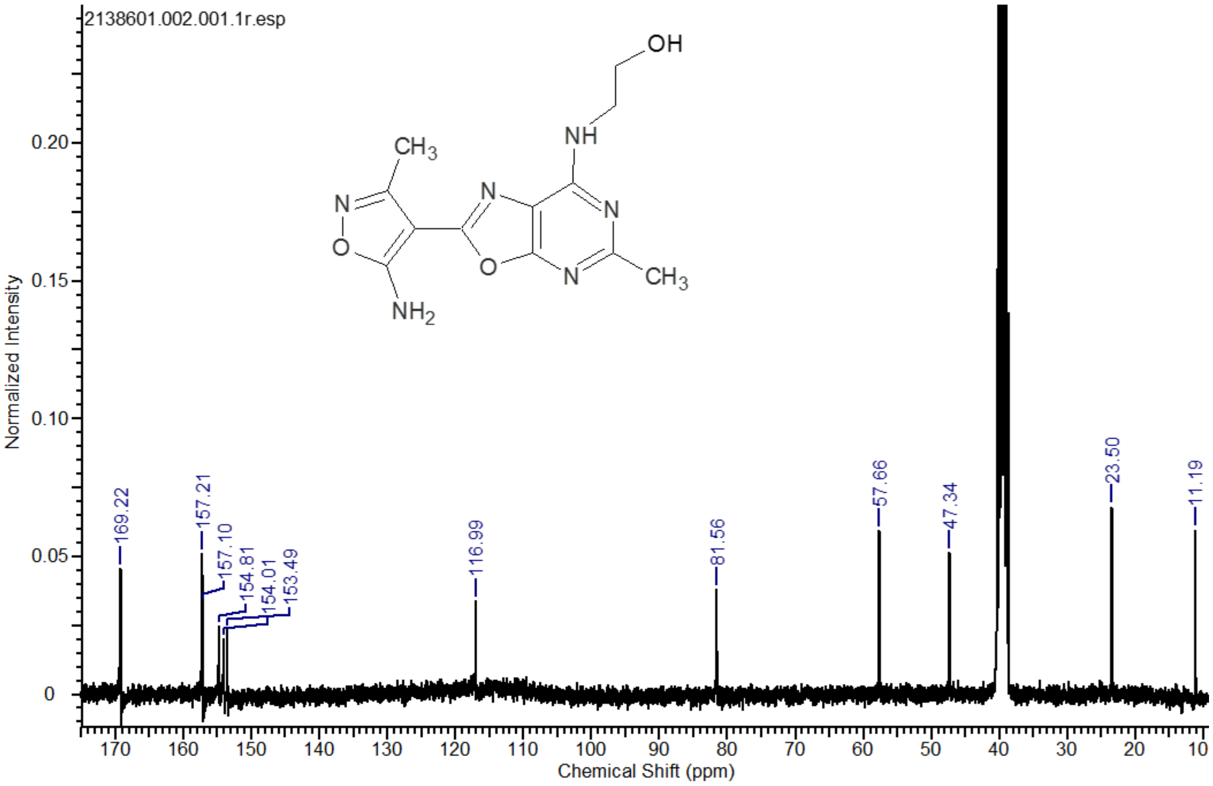

Figure S51. ^{13}C-NMR spectrum of compound **5f** in DMSO-d6.

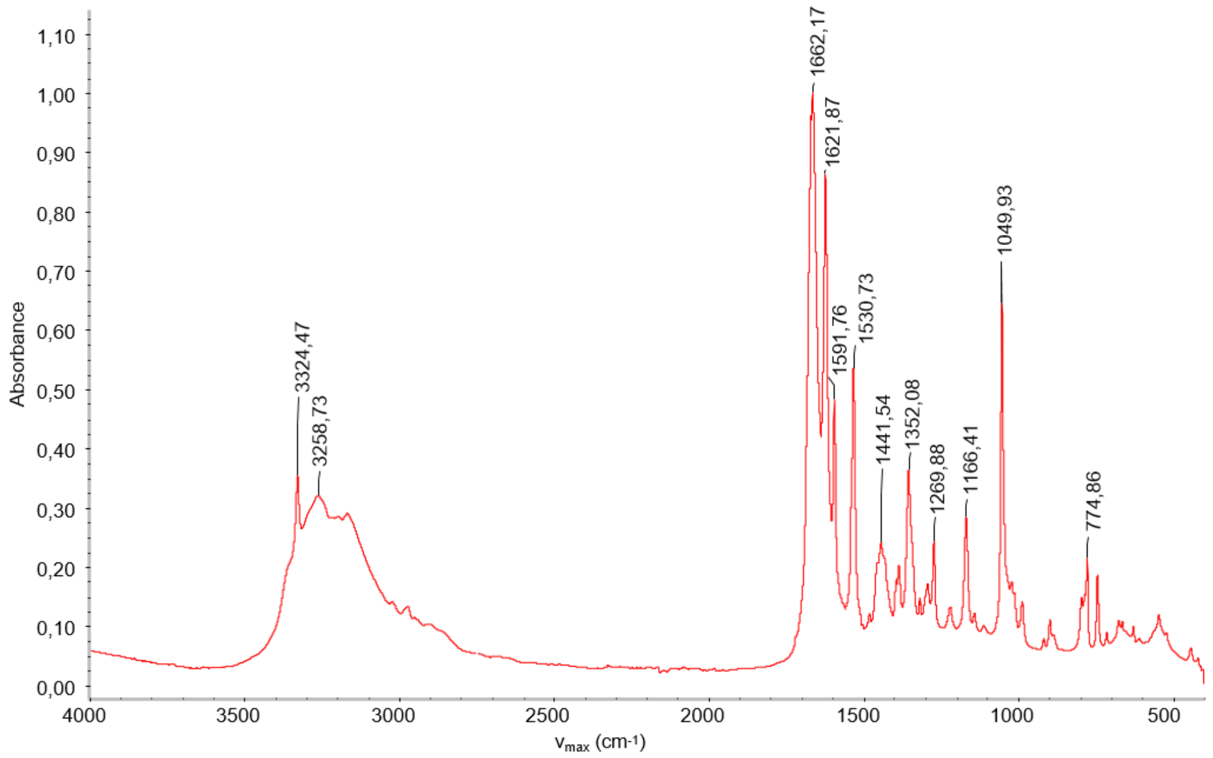

Figure S52. ATR-FTIR spectrum of compound **5f**.

Visualizations of Nuclear magnetic resonance (NMR) and Infrared (IR) spectra of compound 5g

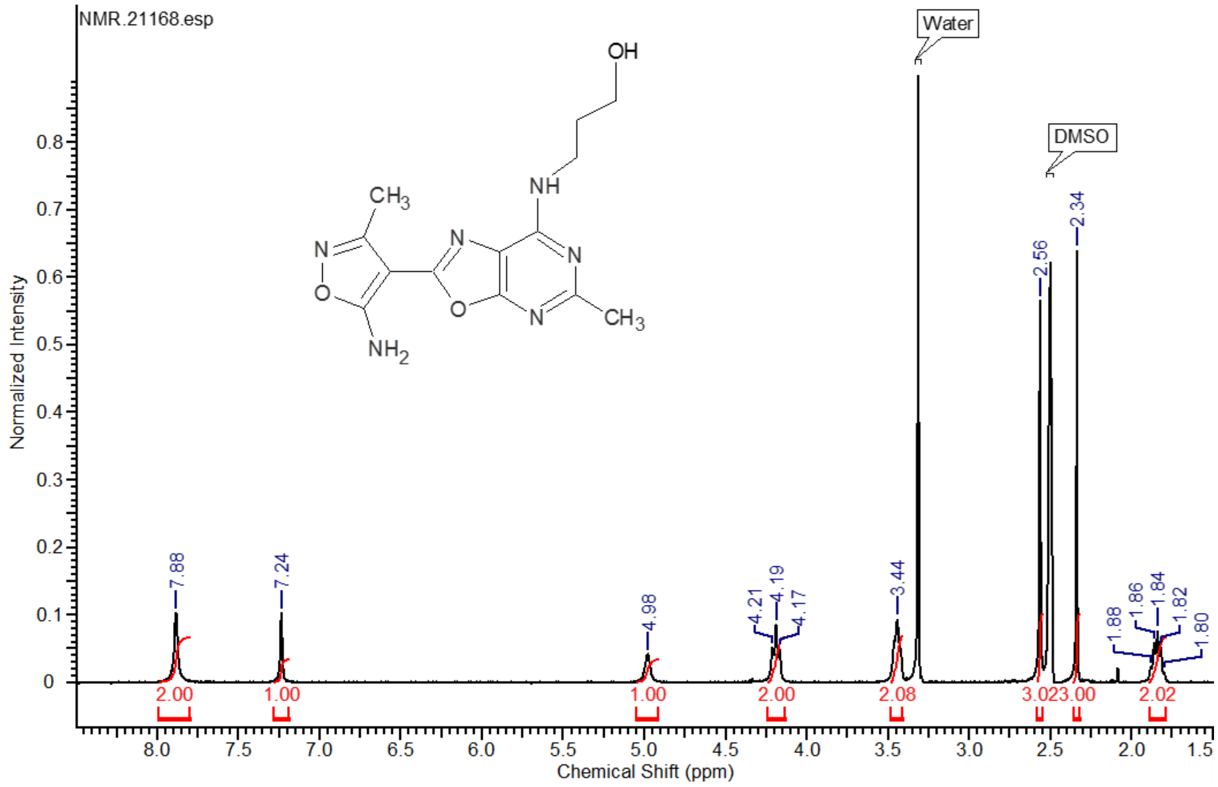

Figure S53. ^{1}H-NMR spectrum of compound **5g** in DMSO-d6.

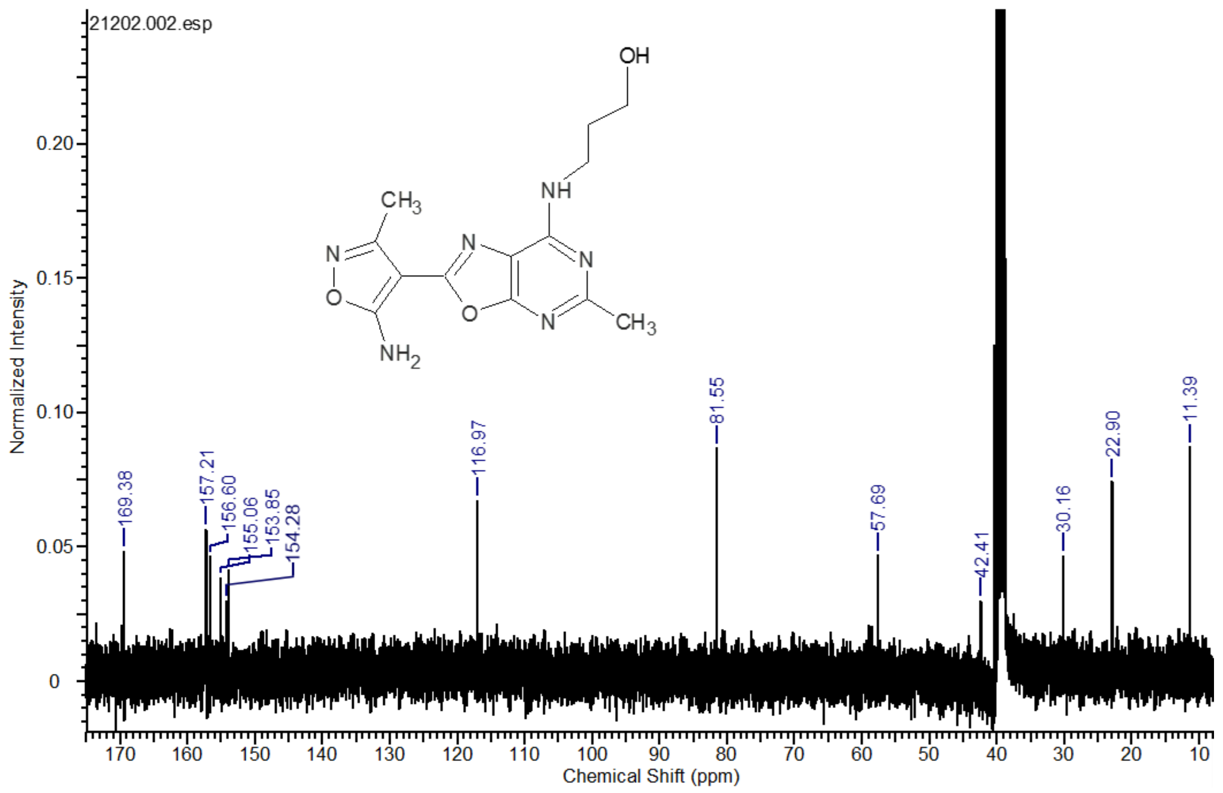

Figure S54. ^{13}C-NMR spectrum of compound **5g** in DMSO-d6.

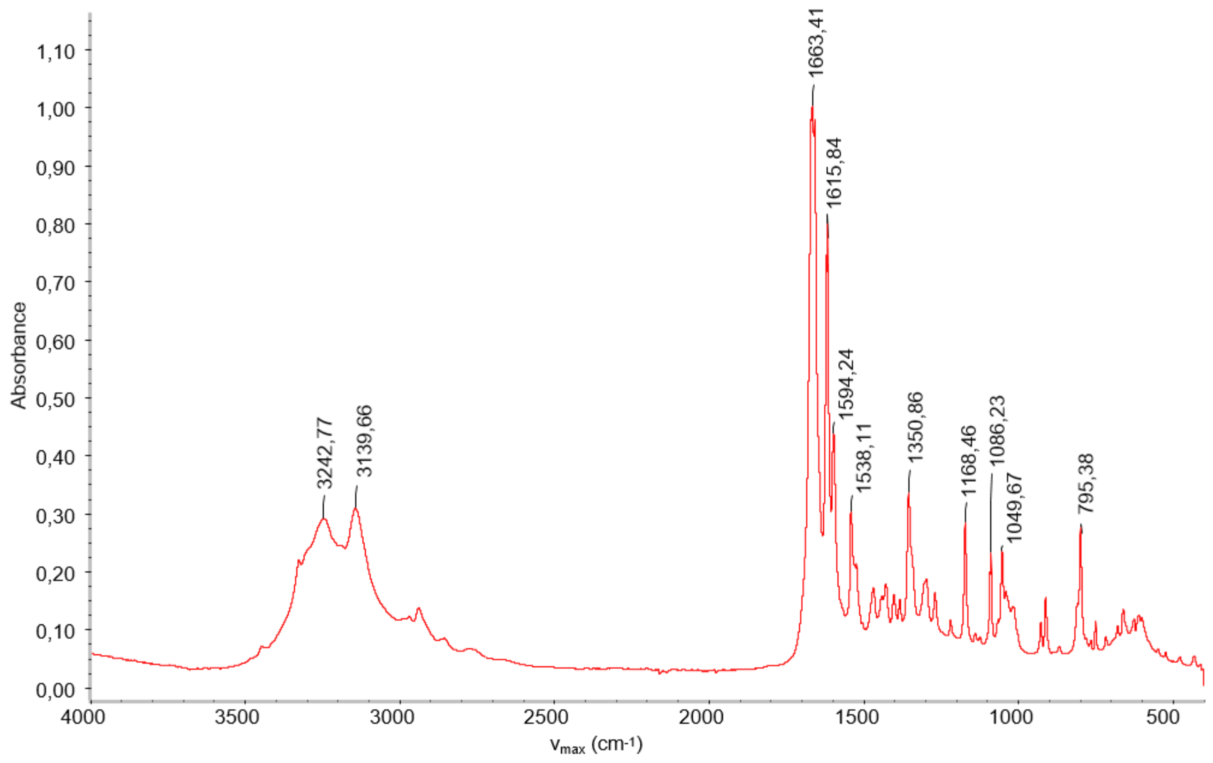

Figure S55. ATR-FTIR spectrum of compound **5g**.

Visualizations of Nuclear magnetic resonance (NMR) and Infrared (IR) spectra of compound 5h

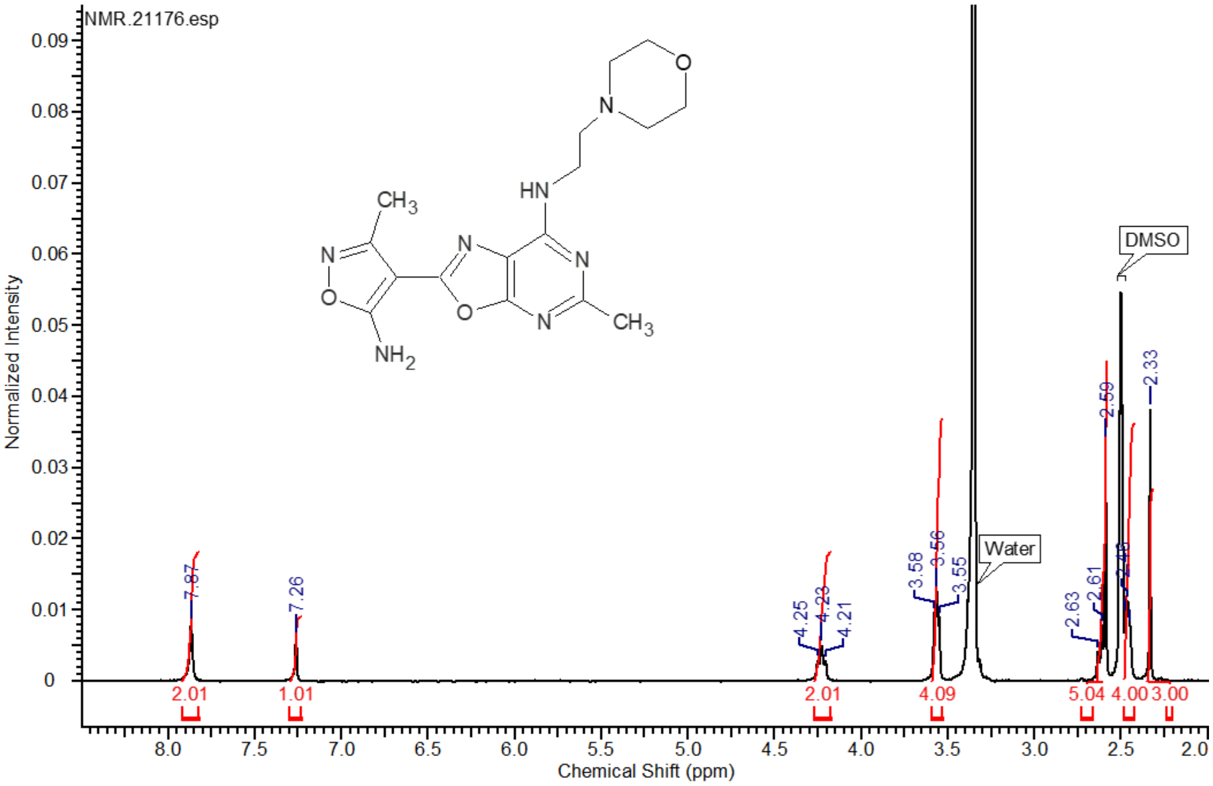

Figure S56. ^{1}H-NMR spectrum of compound **5h** in DMSO-d6.

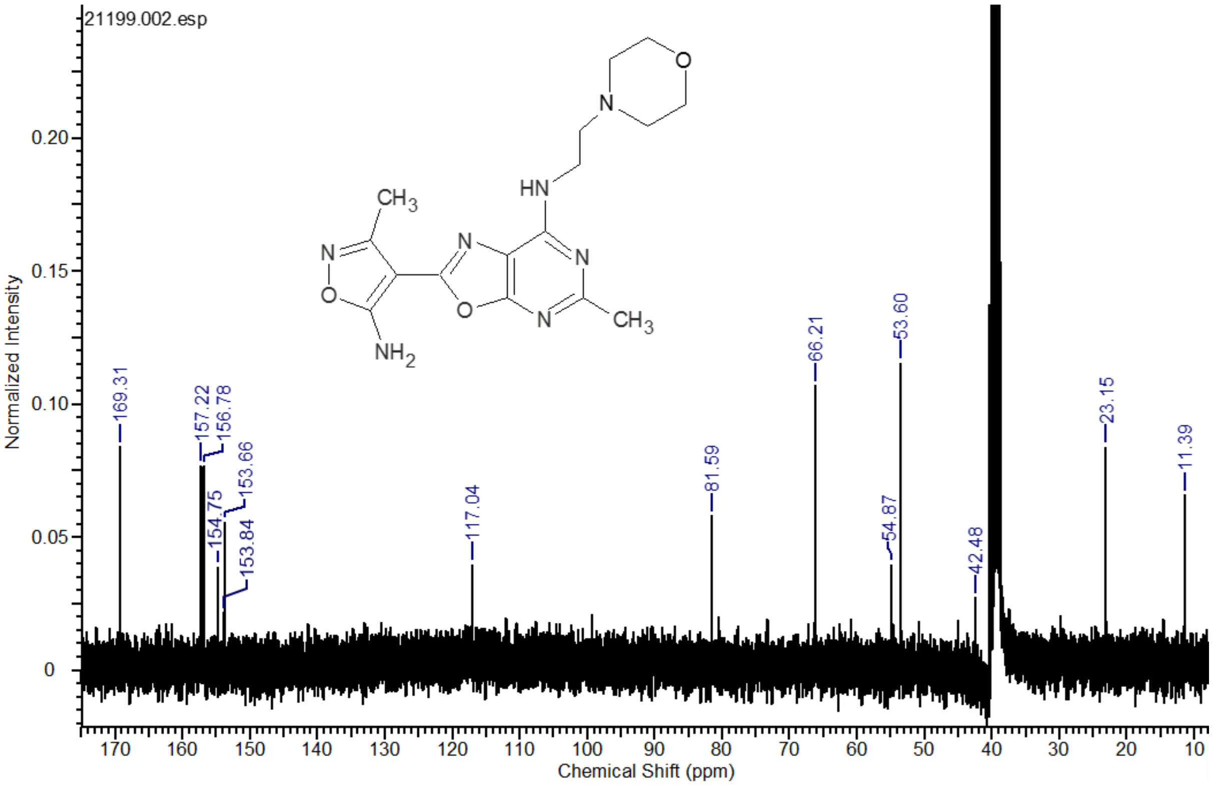

Figure S57. ^{13}C-NMR spectrum of compound **5h** in DMSO-d6.

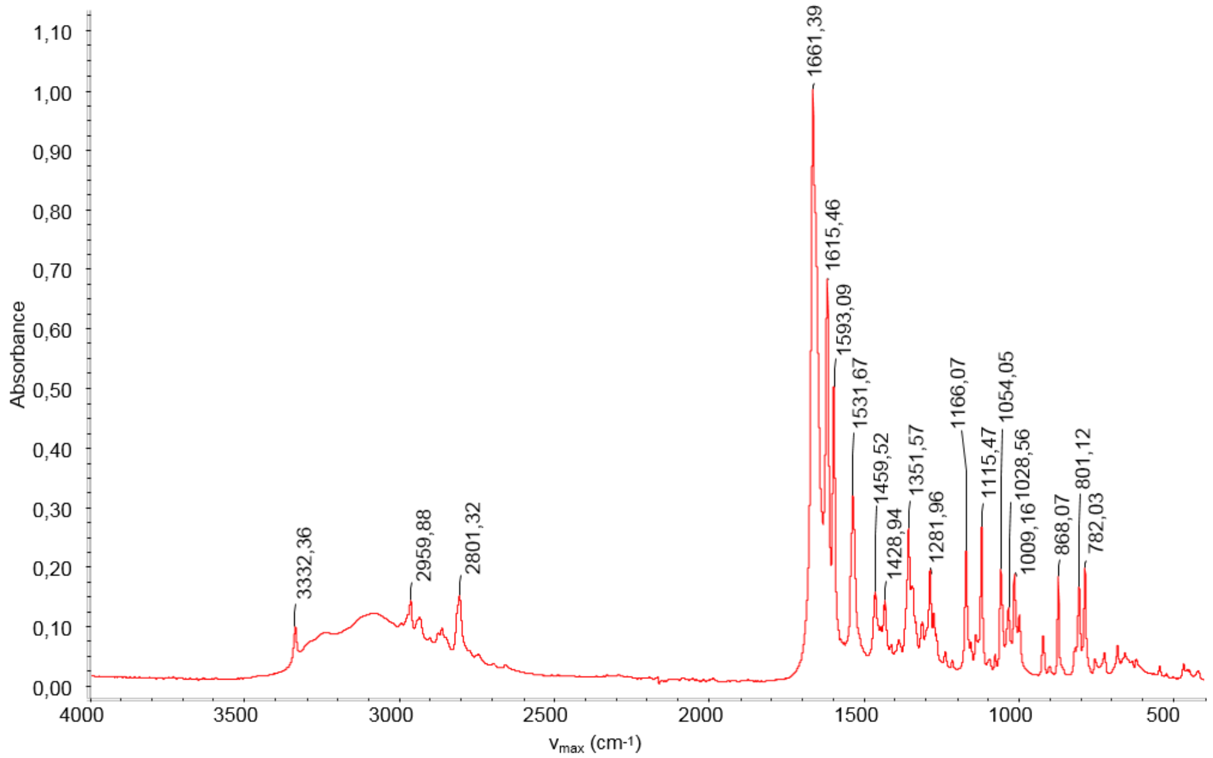

Figure S58. ATR-FTIR spectrum of compound **5h**.

Visualizations of Nuclear magnetic resonance (NMR) and Infrared (IR) spectra of compound 5i

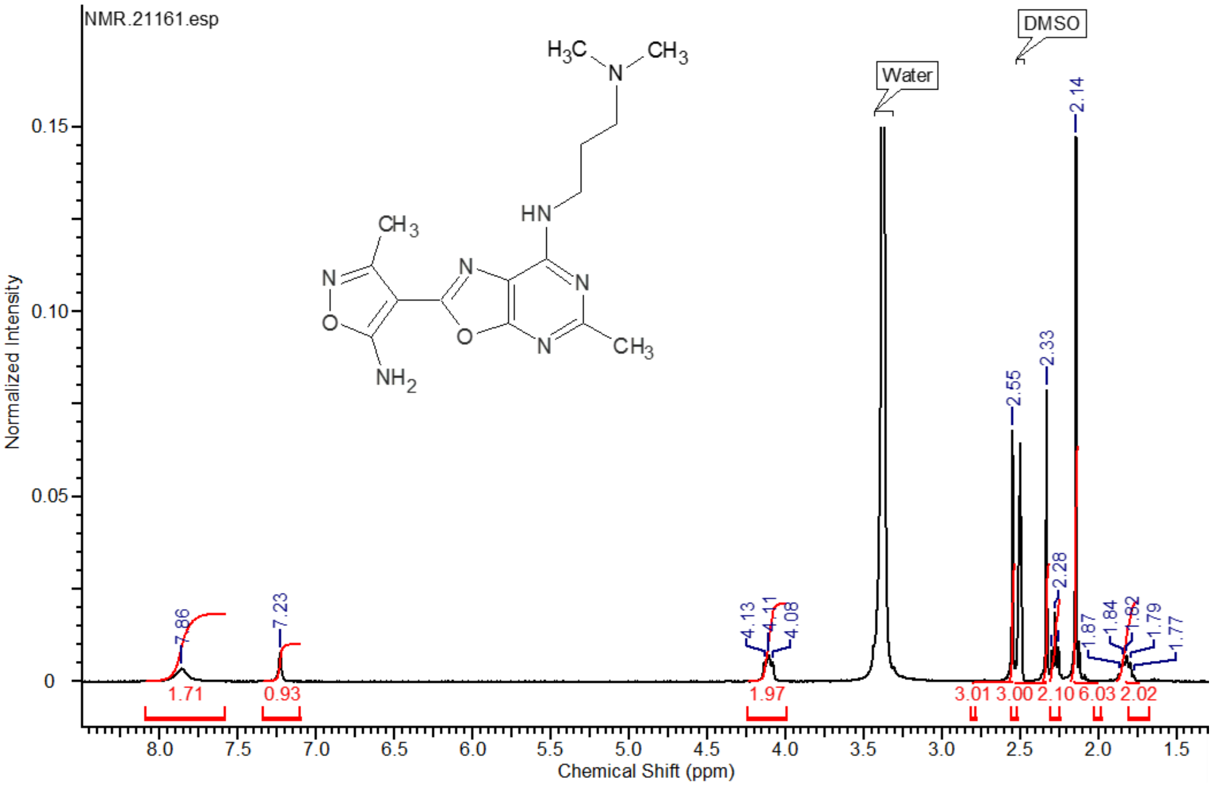

Figure S59. ^{1}H-NMR spectrum of compound **5i** in DMSO-d6 (method A).

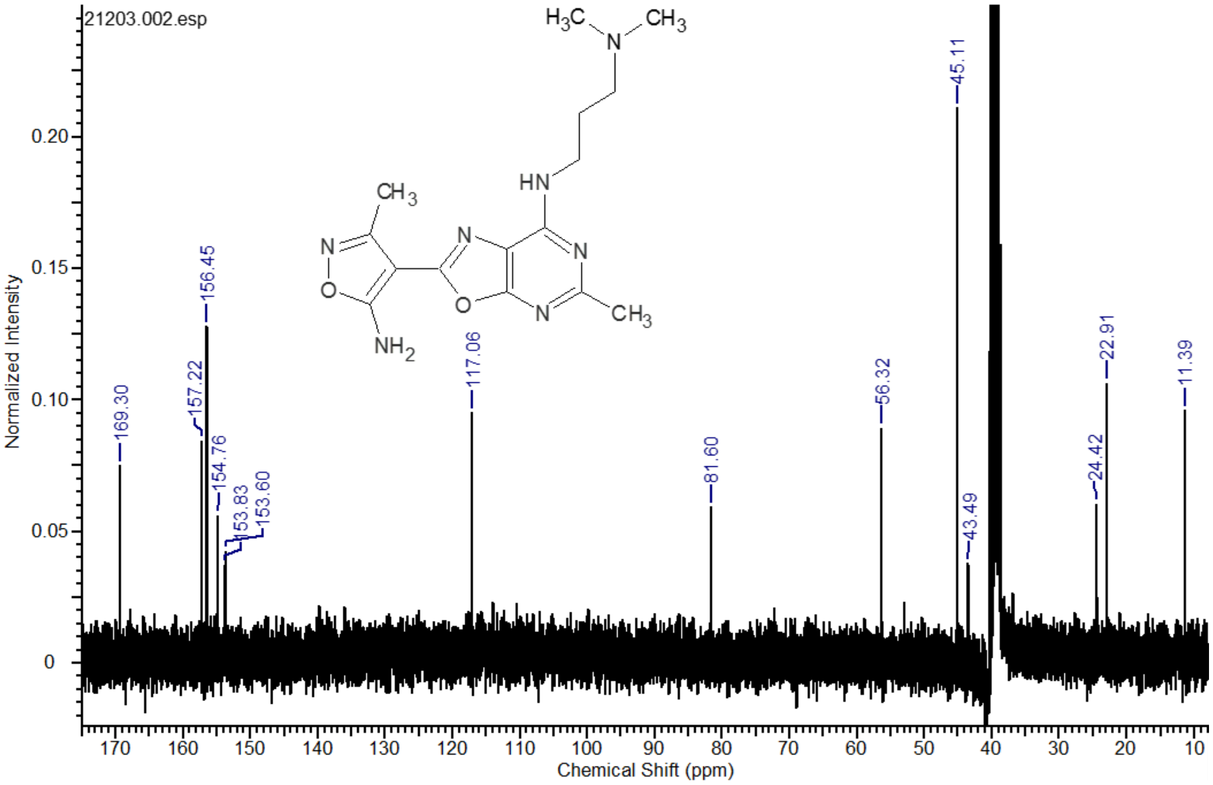

Figure S60. ^{13}C-NMR spectrum of compound **5i** in DMSO-d6 (method A).

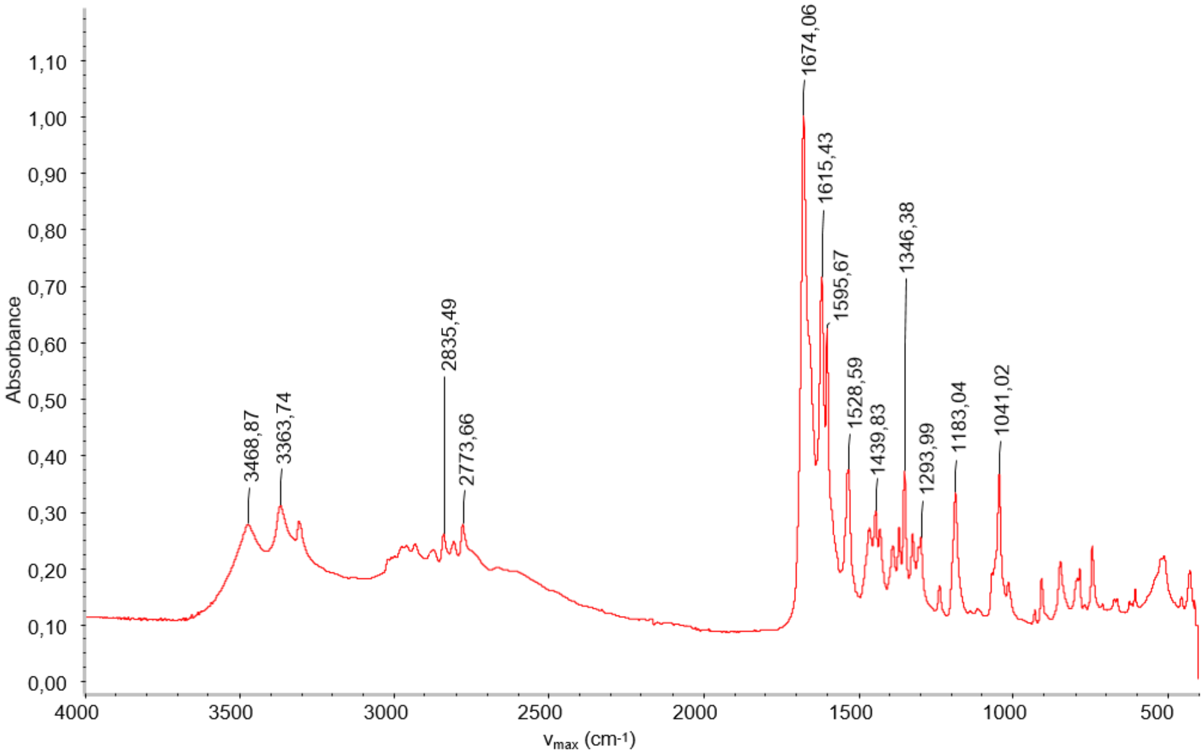

Figure S61. ATR-FTIR spectrum of compound **5i** (method A).

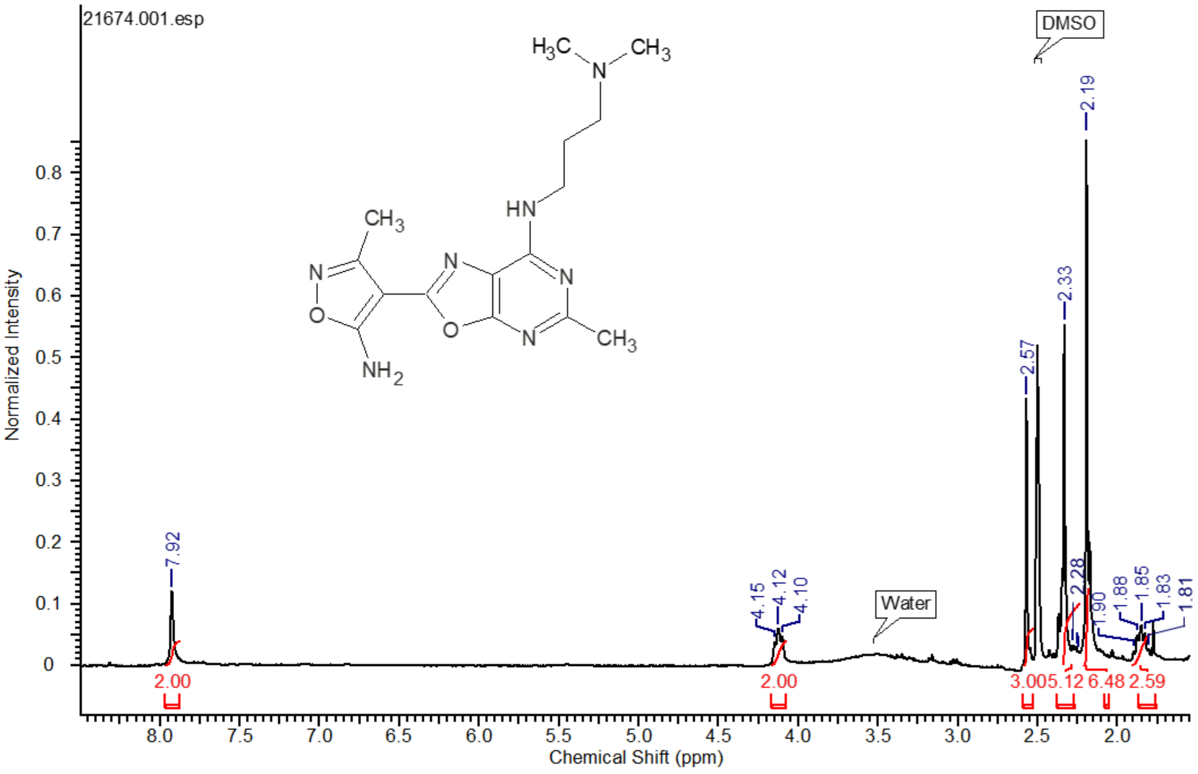

Figure S62. ^{1}H-NMR spectrum of compound **5i** in DMSO-d6 (method B).

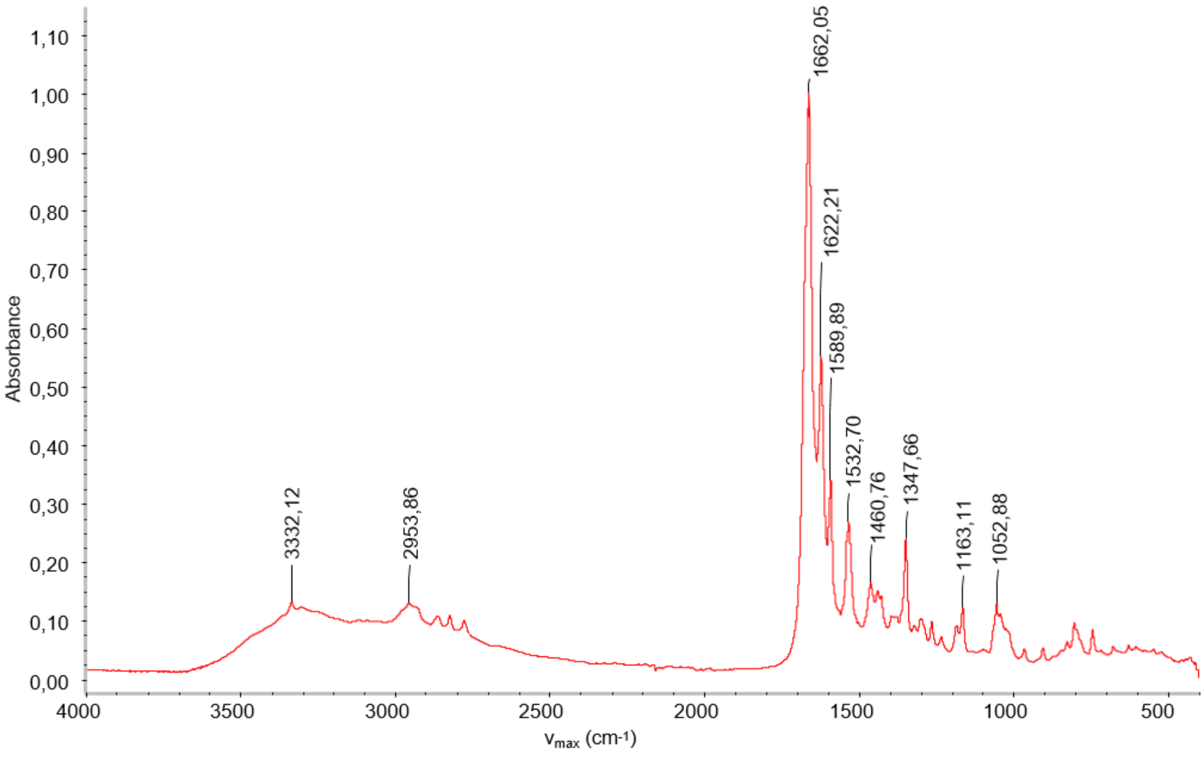

Figure S63. ATR-FTIR spectrum of compound **5i** (method B).

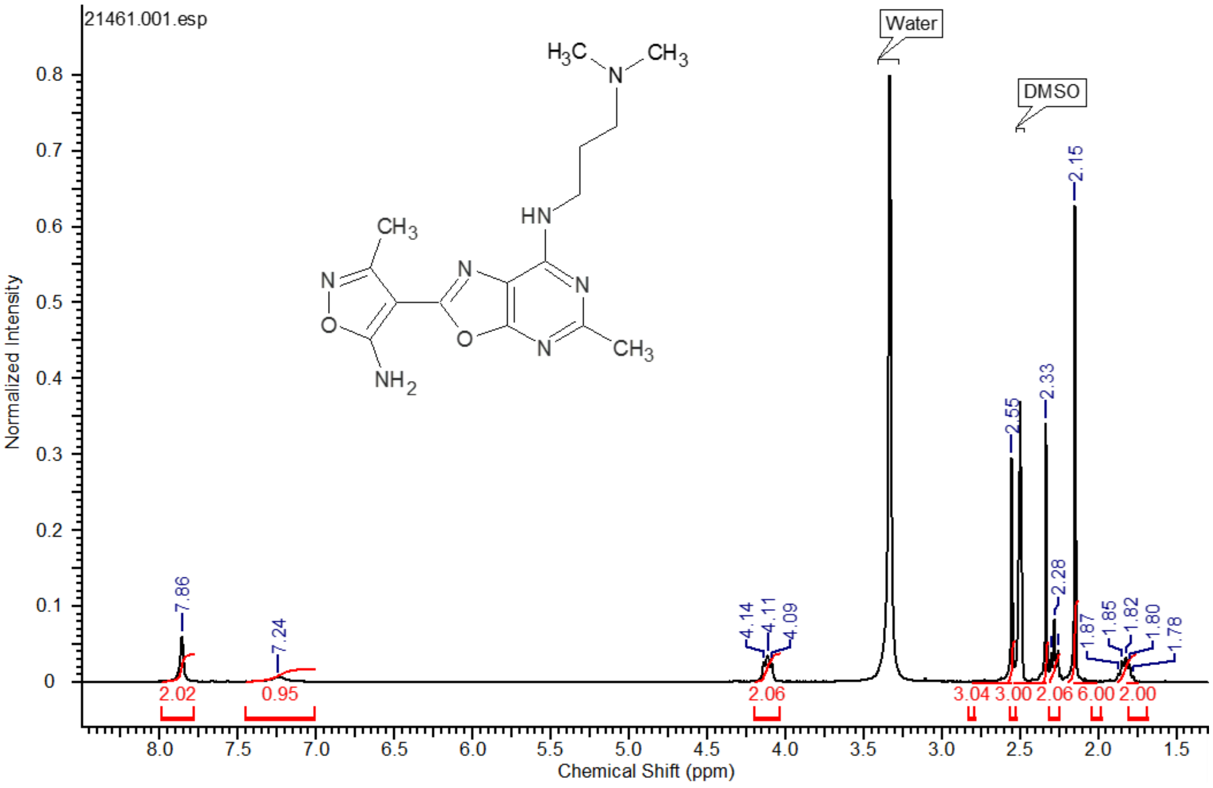

Figure S64. ^{1}H-NMR spectrum of compound **5i** in DMSO-d6 (method C).

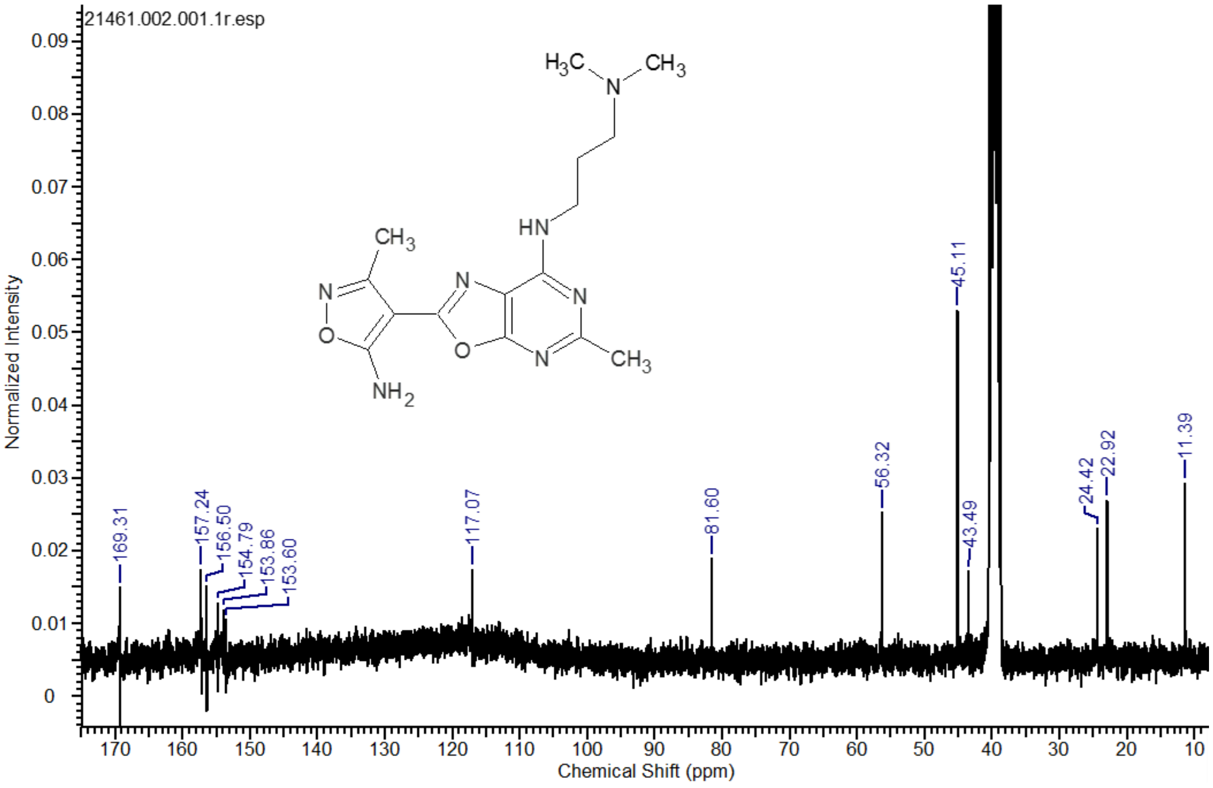

Figure S65. ^{13}C-NMR spectrum of compound **5i** in DMSO-d6 (method C).

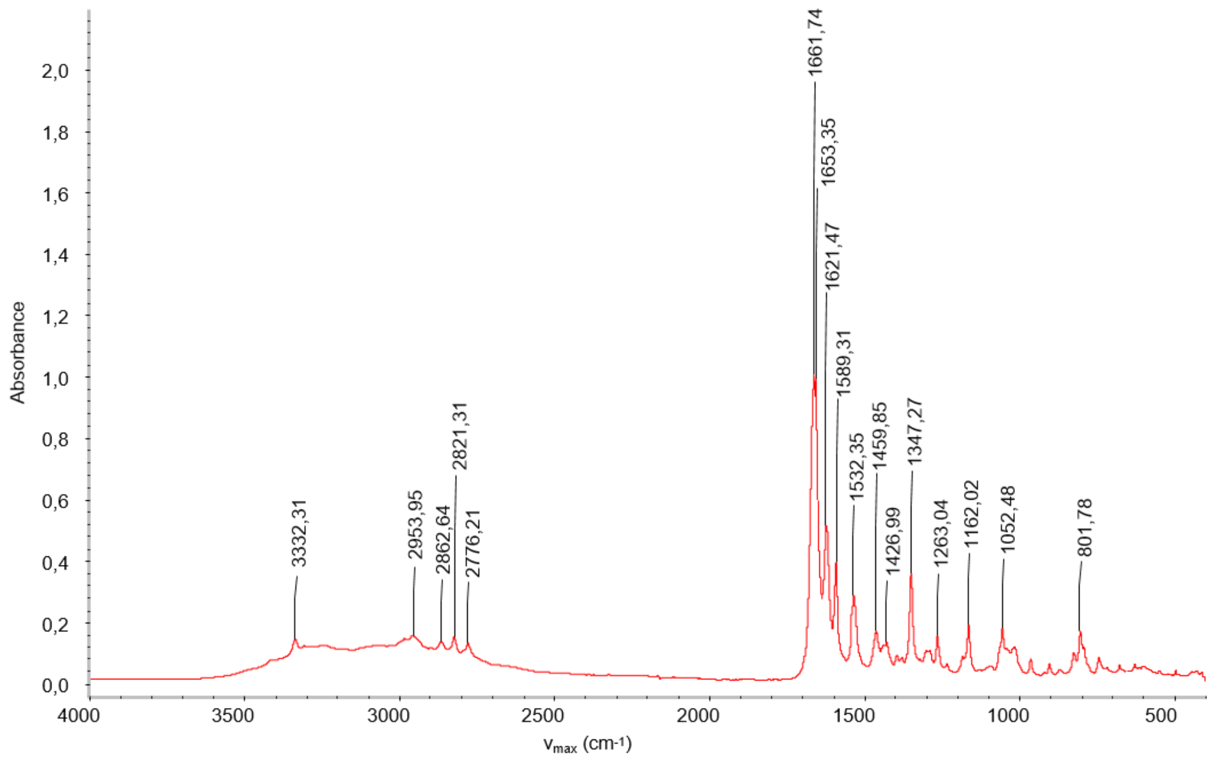

Figure S66. ATR-FTIR spectrum of compound **5i** (method C).

Visualizations of Nuclear magnetic resonance (NMR) and Infrared (IR) spectra of compound 5j

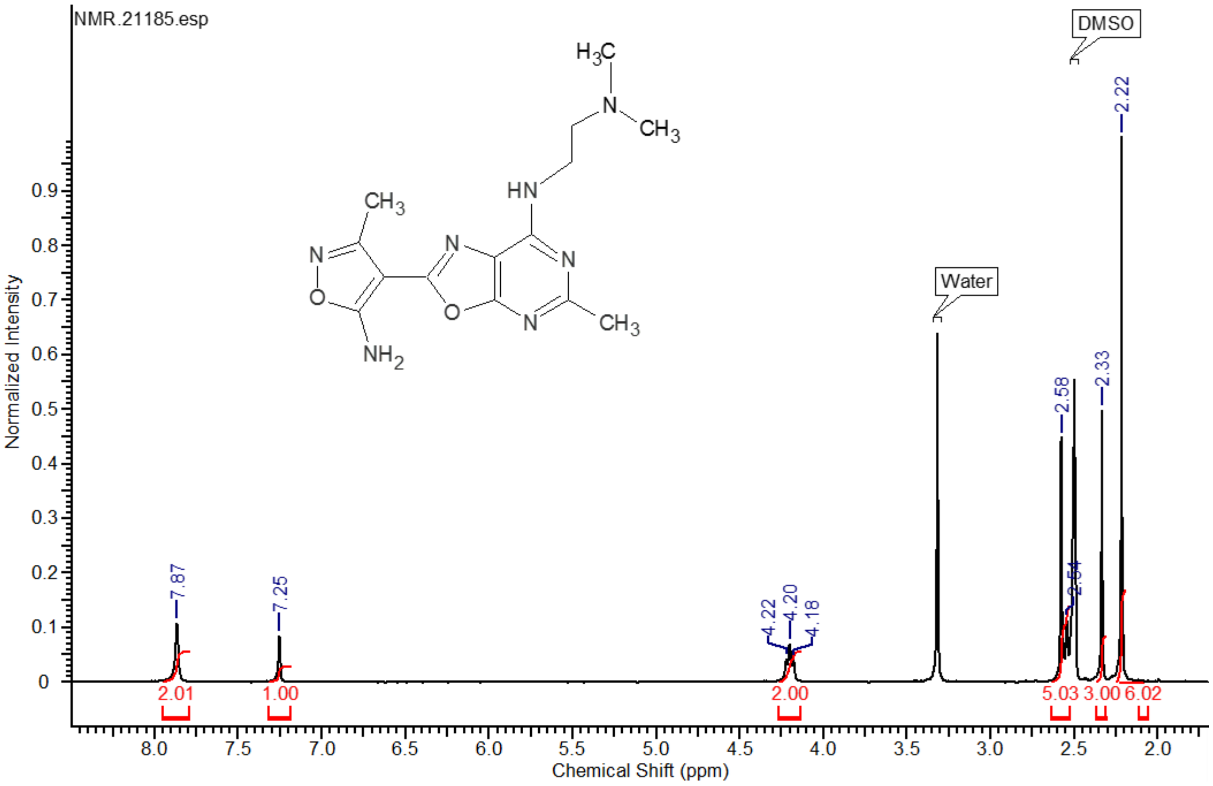

Figure S67. ^{1}H-NMR spectrum of compound **5j** in DMSO-d6.

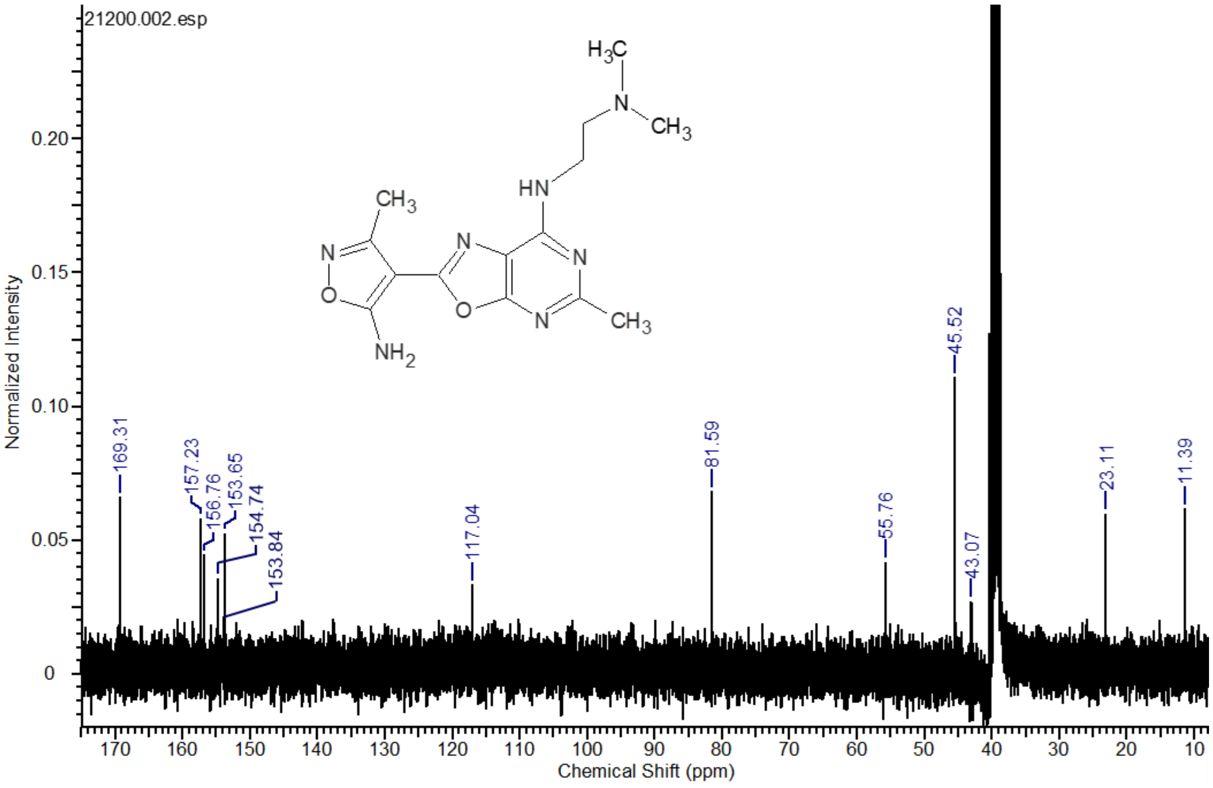

Figure S68. ^{13}C-NMR spectrum of compound **5j** in DMSO-d6.

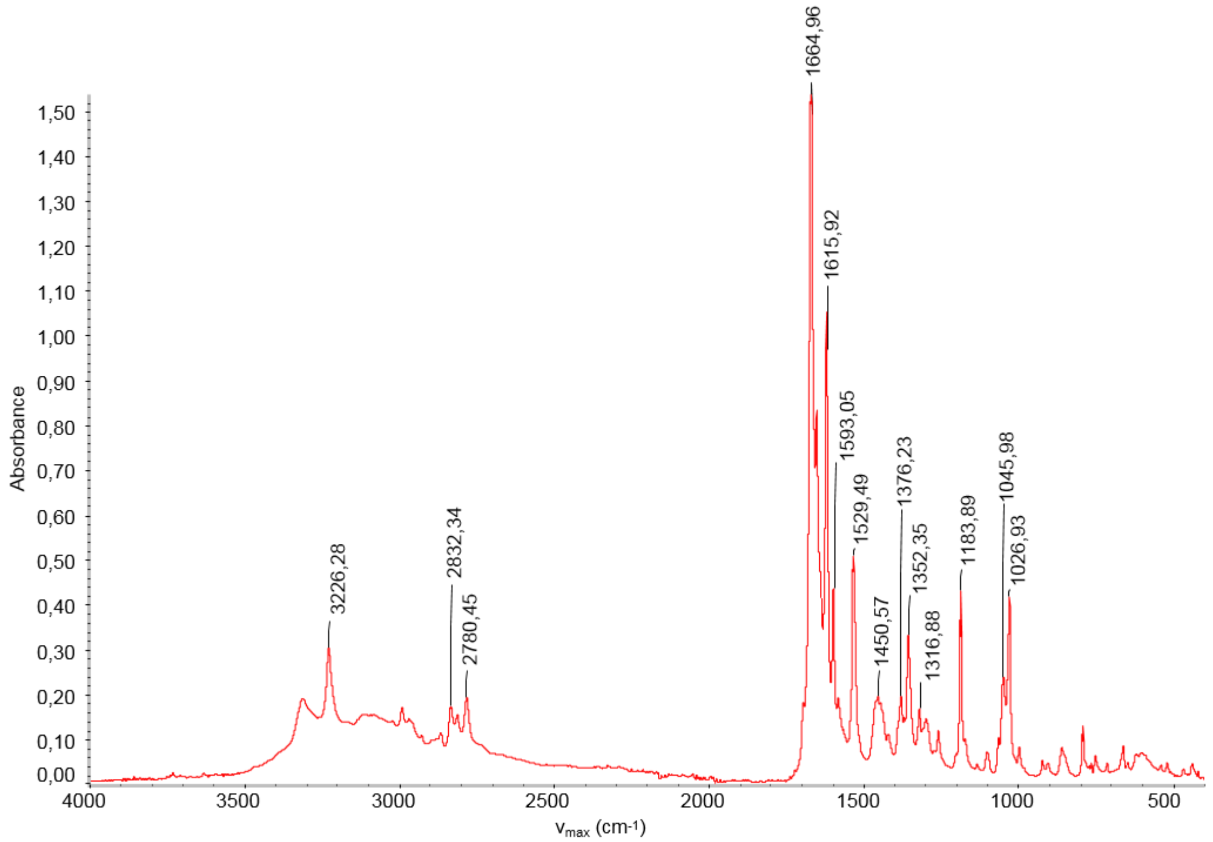

Figure S69. ATR-FTIR spectrum of compound **5j**.

Visualizations of Nuclear magnetic resonance (NMR) and Infrared (IR) spectra of compound 5k

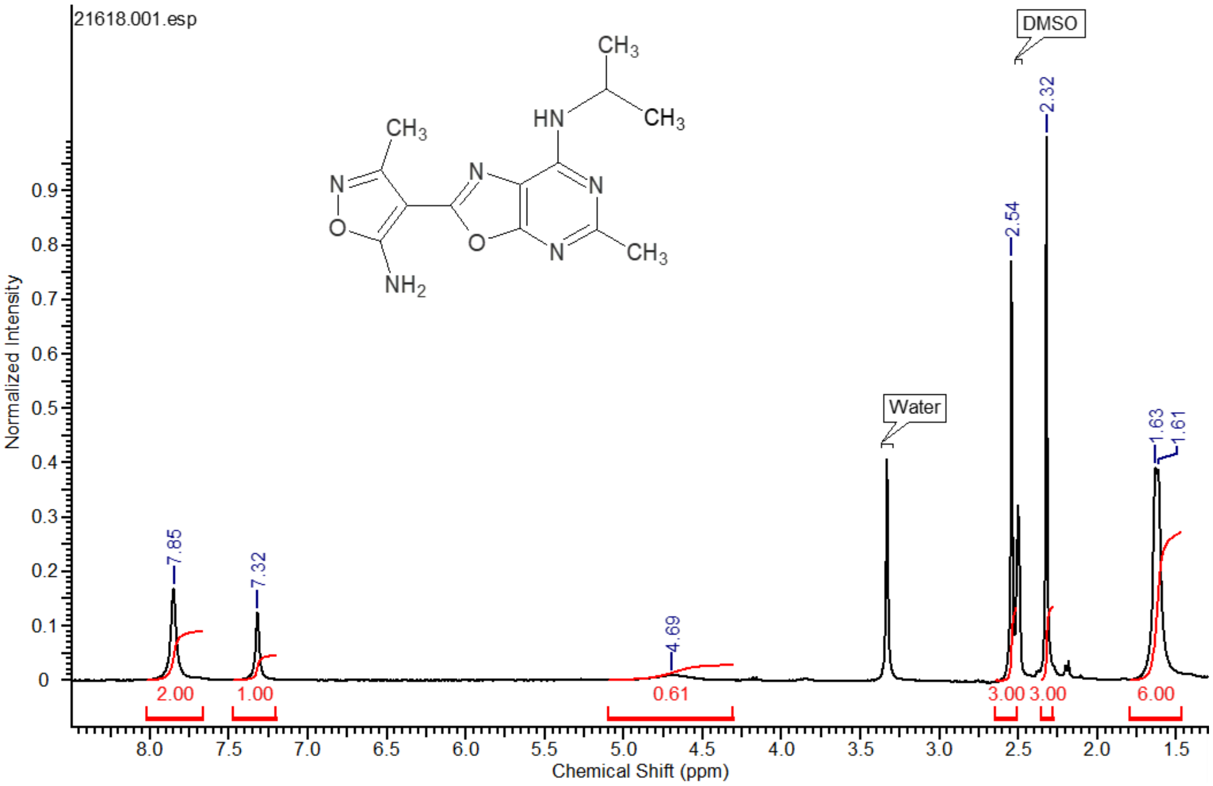

Figure S70. ^{1}H-NMR spectrum of compound **5k** in DMSO-d6.

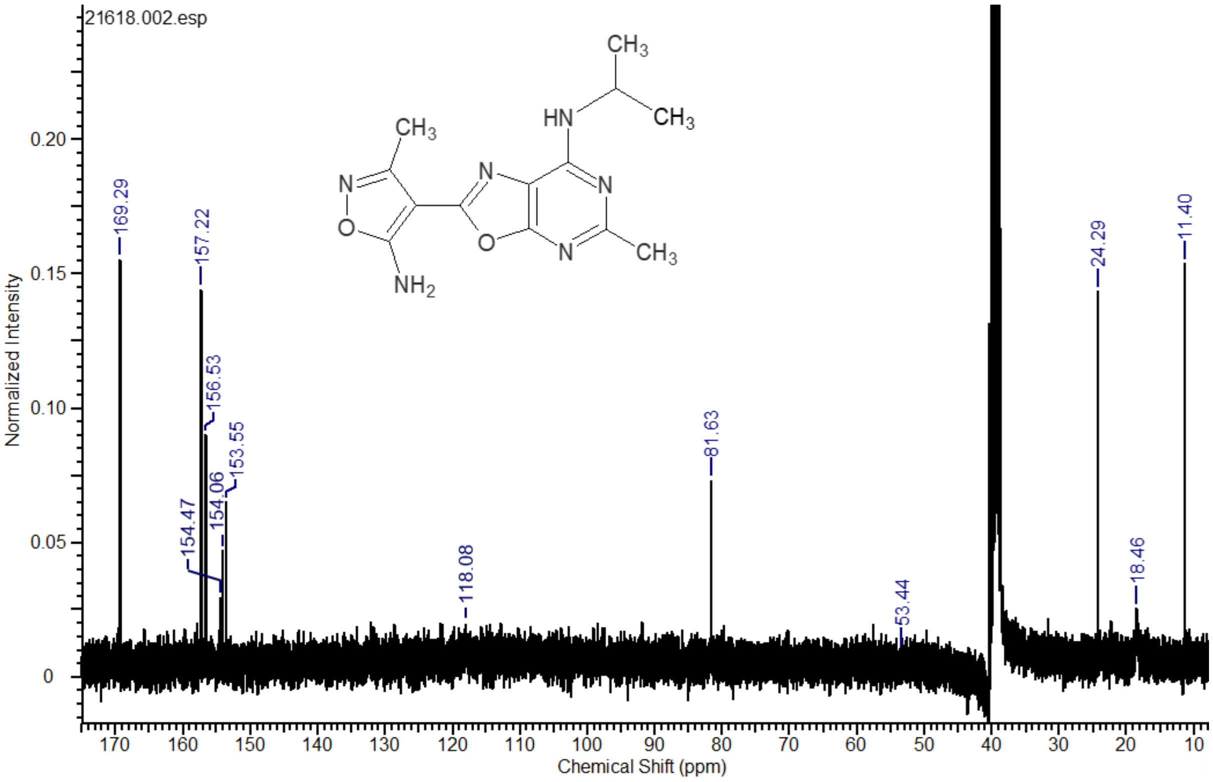

Figure S71. ^{13}C-NMR spectrum of compound **5k** in DMSO-d6.

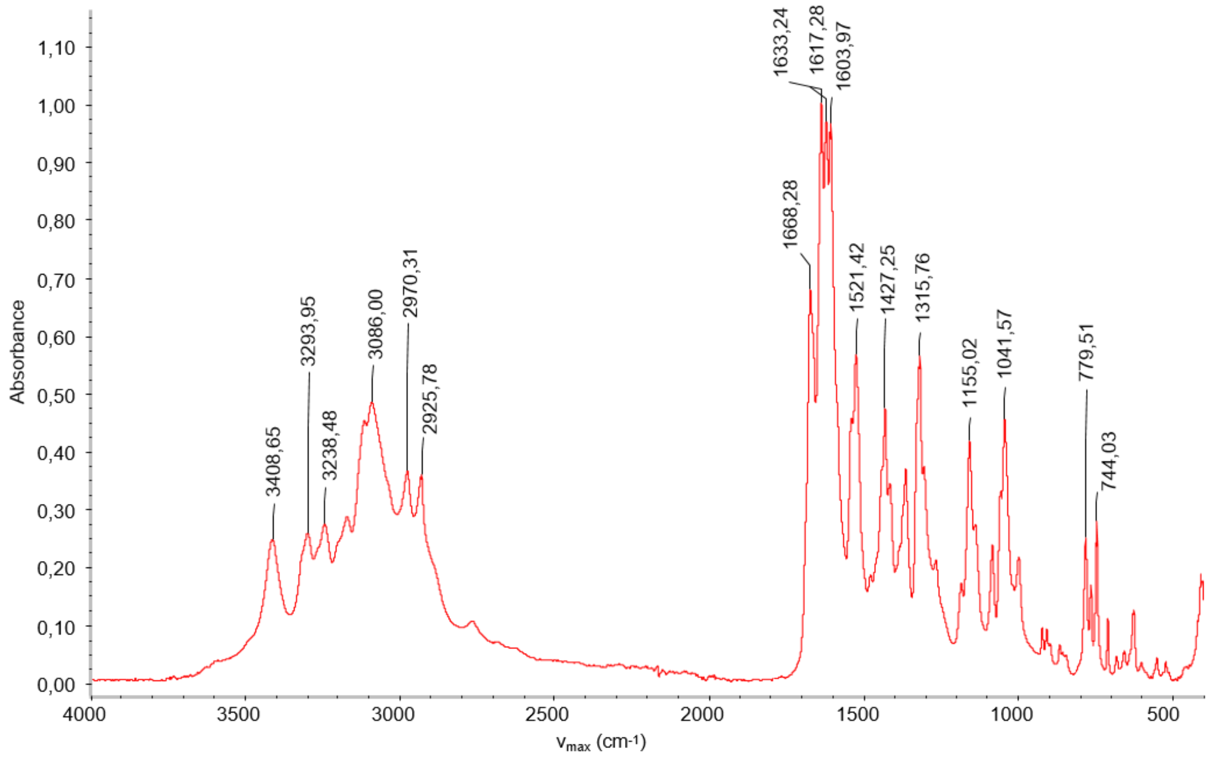

Figure S72. ATR-FTIR spectrum of compound **5k**.

Visualizations of Nuclear magnetic resonance (NMR) and Infrared (IR) spectra of compound 5l

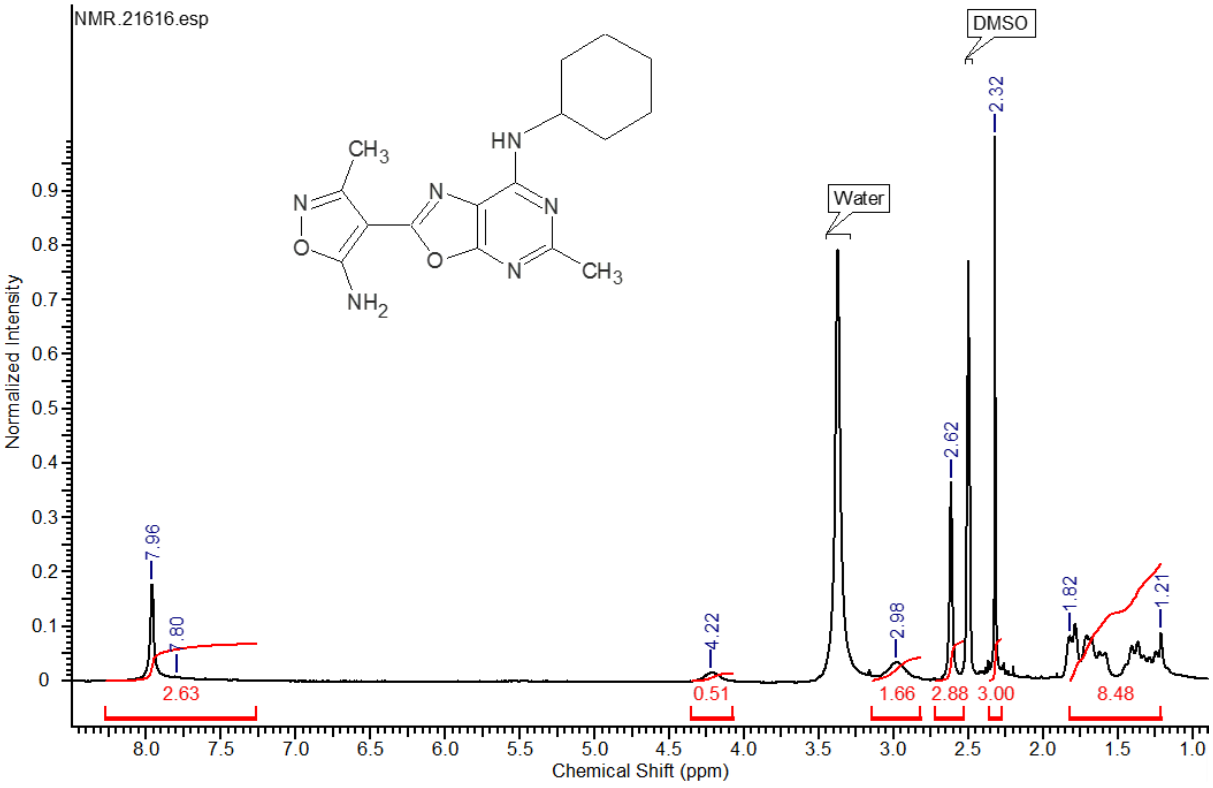

Figure S73. ^{1}H-NMR spectrum of compound **5l** in DMSO-d6.

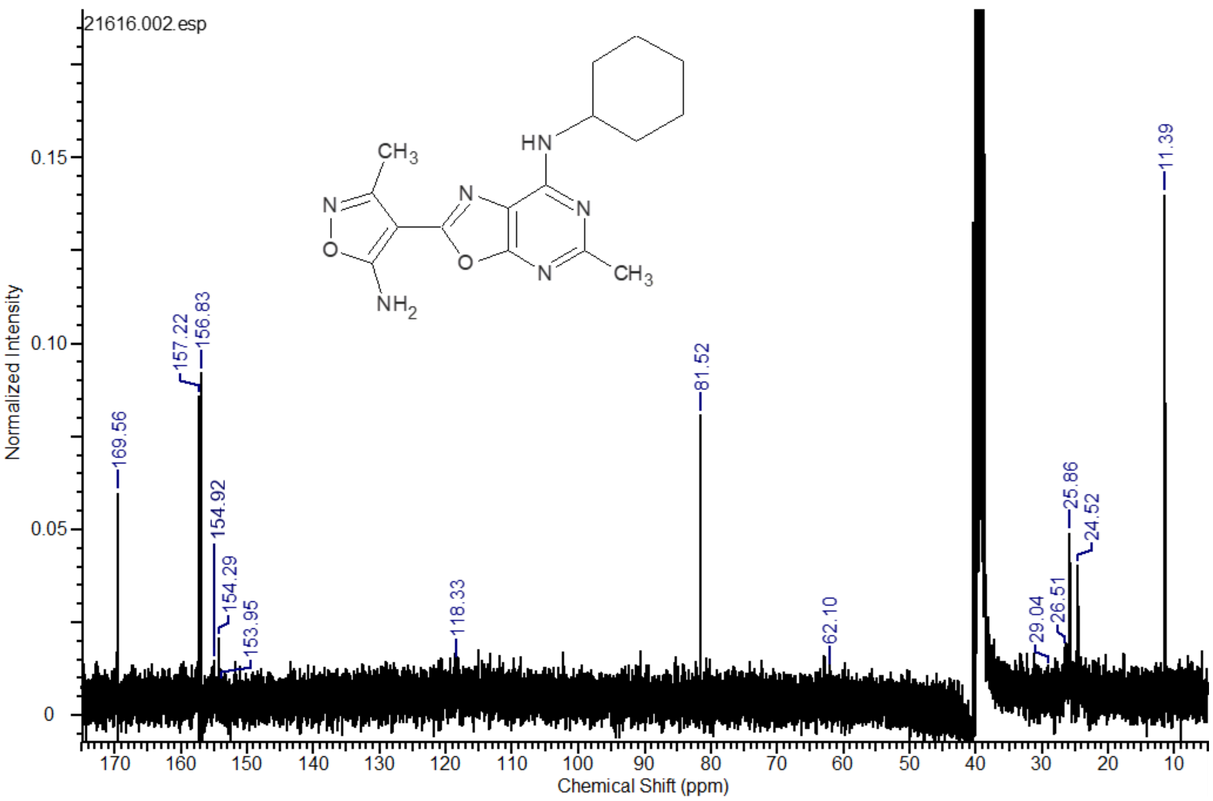

Figure S74. ^{13}C-NMR spectrum of compound **5l** in DMSO-d6.

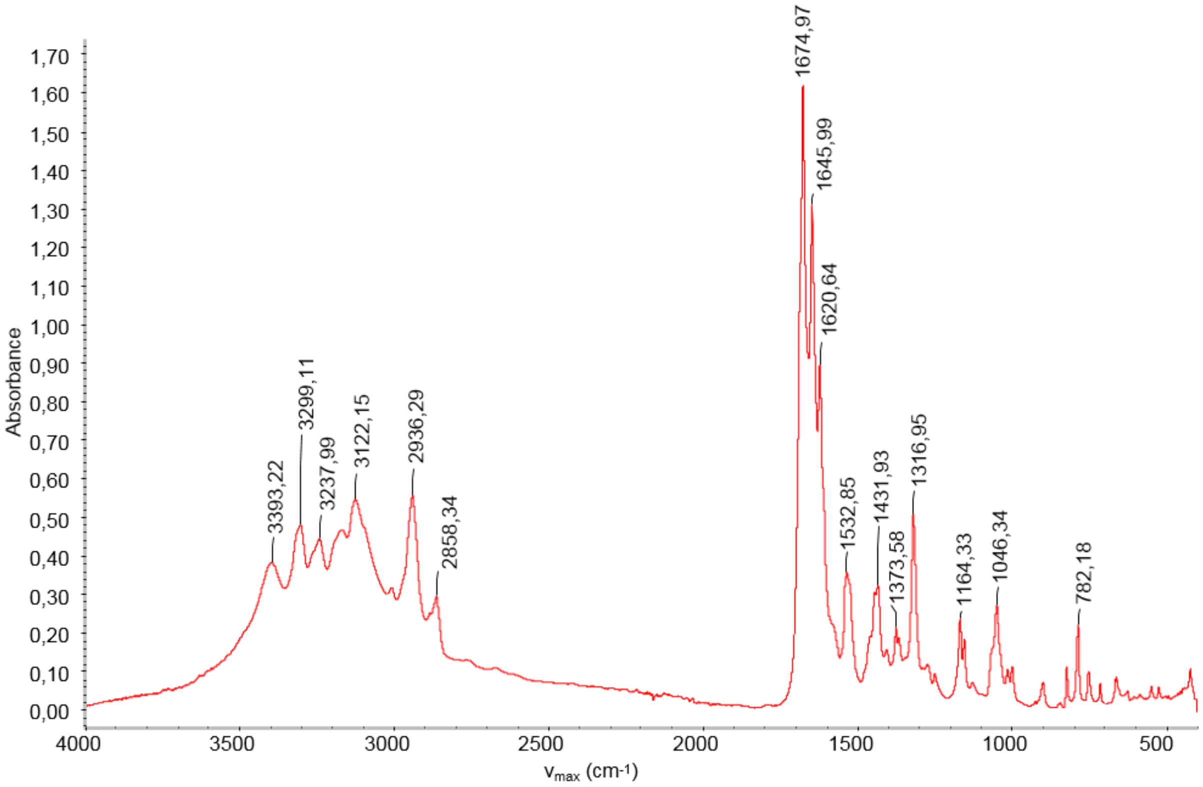

Figure S75. ATR-FTIR spectrum of compound **5l**.

Visualizations of Nuclear magnetic resonance (NMR) and Infrared (IR) spectra of compound 7m

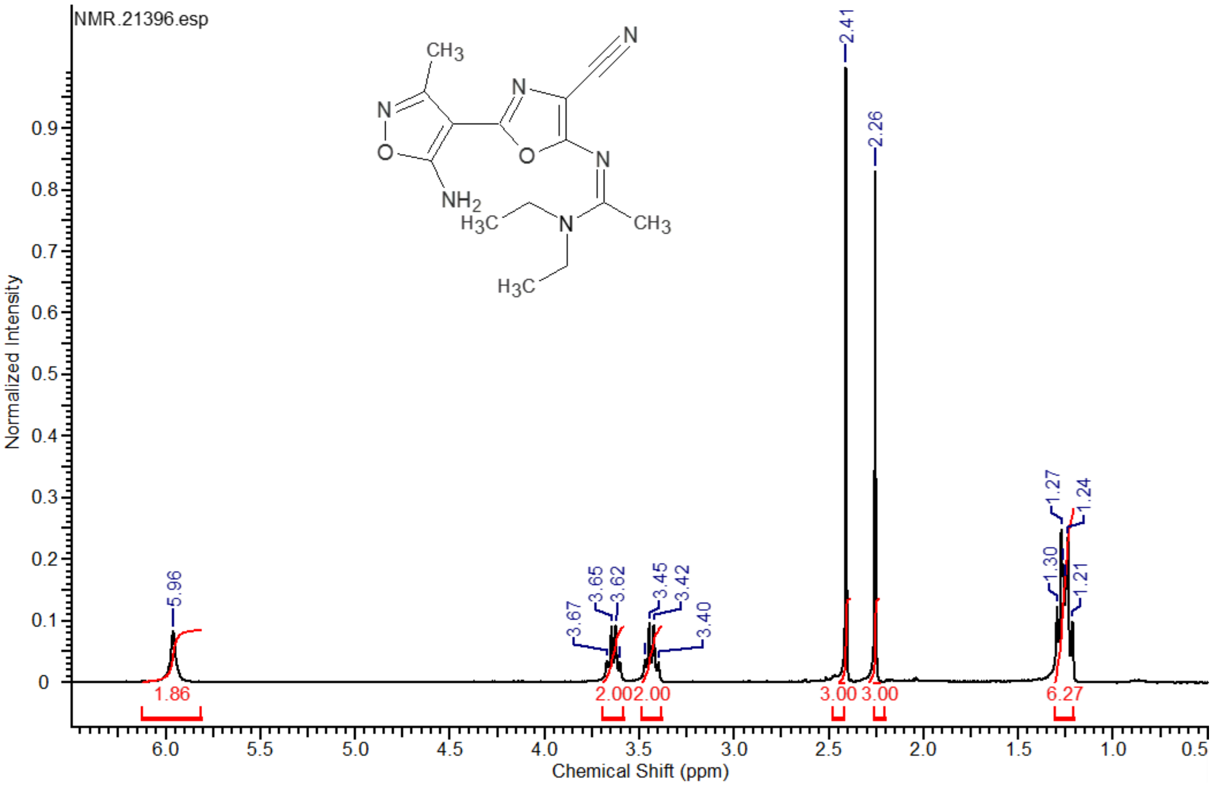

Figure S76. ^{1}H-NMR spectrum of compound **7m** in $CDCl_3$.

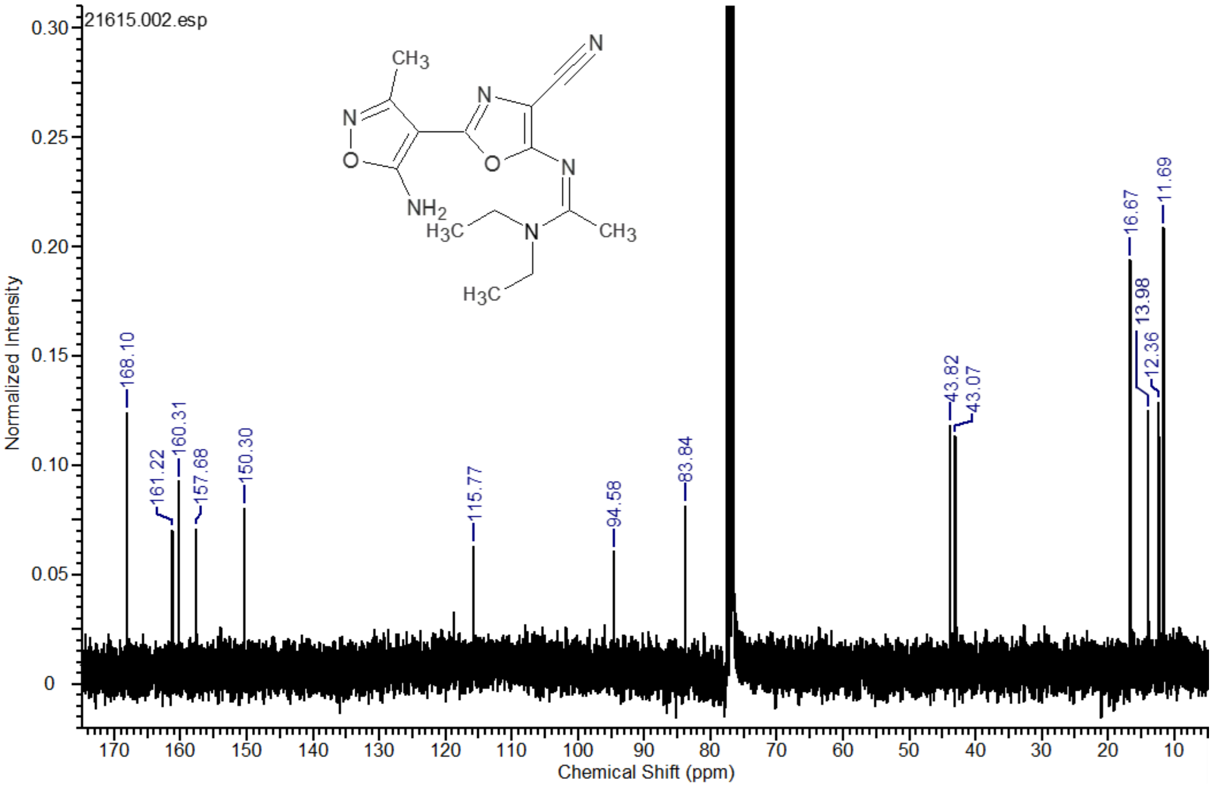

Figure S77. ^{13}C-NMR spectrum of compound **7m** in $CDCl_3$.

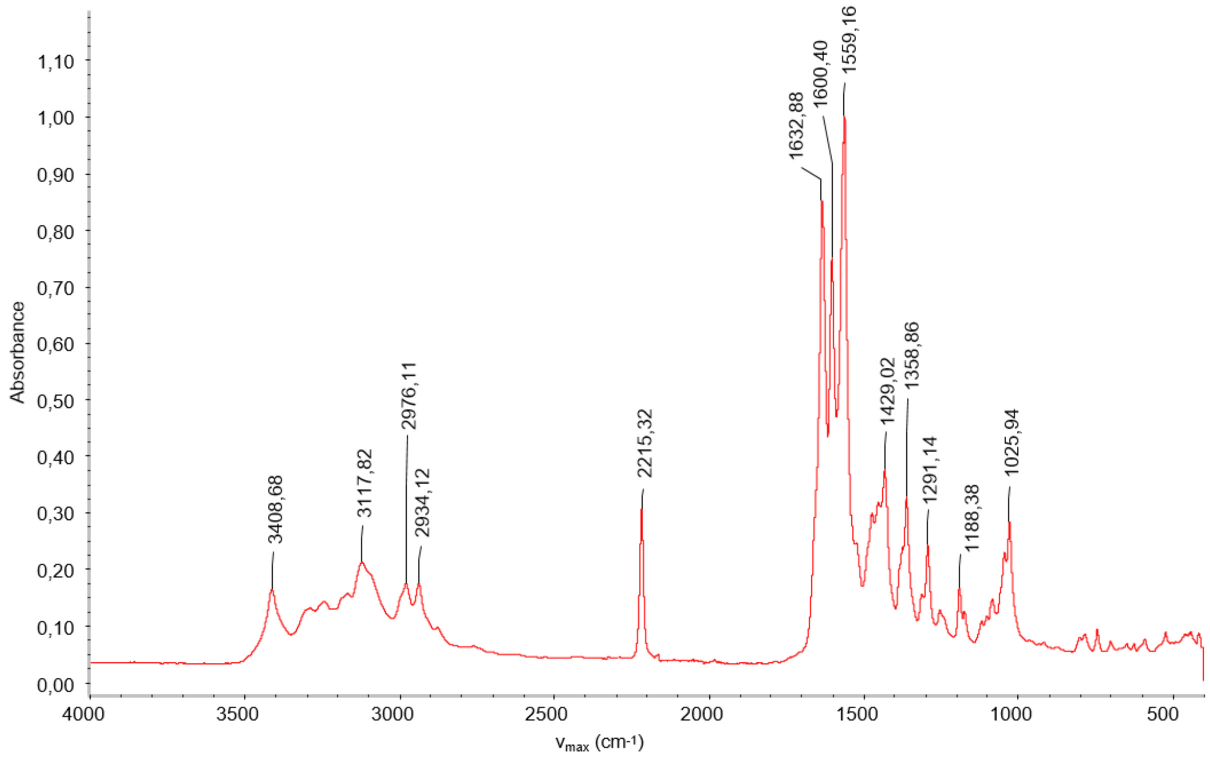

Figure S78. ATR-FTIR spectrum of compound **7m**.

Single crystal X-ray diffraction measurement of compound 5h

Table S25. Selected bond lengths (Å), bond and torsion angles (°) in **5h**.

bond lengths			
O1—C5	1.375 (2)	N6—C11	1.453 (2)
O1—C1	1.3860 (19)	N7—C12	1.460 (2)
O2—C8	1.343 (2)	N7—C16	1.463 (2)
O2—N4	1.436 (2)	N7—C13	1.465 (2)
O3—C15	1.425 (3)	C1—C6	1.426 (2)
O3—C14	1.426 (2)	C2—C10	1.506 (2)
N1—C1	1.295 (2)	C3—C4	1.407 (2)
N1—C4	1.399 (2)	C4—C5	1.372 (2)
N2—C5	1.322 (2)	C6—C8	1.381 (2)
N2—C2	1.347 (2)	C6—C7	1.418 (2)
N3—C2	1.336 (2)	C7—C9	1.485 (3)
N3—C3	1.358 (2)	C11—C12	1.519 (2)

N4—C7	1.307 (2)	C13—C14	1.506 (3)
N5—C8	1.327 (2)	C15—C16	1.512 (3)
N6—C3	1.341 (2)		
bond angles			
C5—O1—C1	103.53 (13)	C5—C4—C3	115.82 (15)
C8—O2—N4	107.87 (13)	N1—C4—C3	134.60 (15)
C15—O3—C14	111.50 (15)	N2—C5—C4	129.10 (15)
C1—N1—C4	103.86 (13)	N2—C5—O1	122.99 (15)
C5—N2—C2	110.47 (14)	C4—C5—O1	107.90 (14)
C2—N3—C3	119.46 (14)	C8—C6—C7	104.49 (15)
C7—N4—O2	106.20 (13)	C8—C6—C1	122.96 (16)
C3—N6—C11	124.69 (14)	C7—C6—C1	132.54 (16)
C12—N7—C16	112.34 (14)	N4—C7—C6	111.57 (16)
C12—N7—C13	109.50 (14)	N4—C7—C9	119.38 (16)
C16—N7—C13	108.34 (14)	C6—C7—C9	129.04 (16)
N1—C1—O1	115.14 (14)	N5—C8—O2	117.43 (15)
N1—C1—C6	125.72 (15)	N5—C8—C6	132.71 (15)
O1—C1—C6	119.14 (14)	O2—C8—C6	109.86 (15)
N3—C2—N2	127.64 (15)	N6—C11—C12	112.64 (15)
N3—C2—C10	116.16 (15)	N7—C12—C11	112.02 (14)
N2—C2—C10	116.19 (15)	N7—C13—C14	110.12 (15)
N6—C3—N3	117.36 (14)	O3—C14—C13	112.02 (16)
N6—C3—C4	125.15 (15)	O3—C15—C16	111.97 (18)
N3—C3—C4	117.49 (15)	N7—C16—C15	109.50 (17)
C5—C4—N1	109.57 (14)		
torsion angles			
C8—O2—N4—C7	0.20 (19)	O1—C1—C6—C8	−179.98 (15)
C4—N1—C1—O1	0.39 (18)	N1—C1—C6—C7	179.03 (18)
C4—N1—C1—C6	−179.94 (16)	O1—C1—C6—C7	−1.3 (3)
C5—O1—C1—N1	−0.08 (18)	O2—N4—C7—C6	0.2 (2)
C5—O1—C1—C6	−179.77 (14)	O2—N4—C7—C9	−178.91 (16)
C3—N3—C2—N2	1.3 (3)	C8—C6—C7—N4	−0.4 (2)
C3—N3—C2—C10	−179.82 (15)	C1—C6—C7—N4	−179.29 (17)
C5—N2—C2—N3	−0.2 (2)	C8—C6—C7—C9	178.51 (19)

C5—N2—C2—C10	−179.11 (15)	C1—C6—C7—C9	−0.3 (3)
C11—N6—C3—N3	170.98 (15)	N4—O2—C8—N5	179.59 (15)
C11—N6—C3—C4	−9.0 (3)	N4—O2—C8—C6	−0.49 (19)
C2—N3—C3—N6	178.86 (15)	C7—C6—C8—N5	−179.53 (19)
C2—N3—C3—C4	−1.2 (2)	C1—C6—C8—N5	−0.5 (3)
C1—N1—C4—C5	−0.55 (18)	C7—C6—C8—O2	0.57 (19)
C1—N1—C4—C3	−179.63 (18)	C1—C6—C8—O2	179.55 (15)
N6—C3—C4—C5	−179.83 (16)	C3—N6—C11—C12	86.5 (2)
N3—C3—C4—C5	0.2 (2)	C16—N7—C12—C11	−65.50 (19)
N6—C3—C4—N1	−0.8 (3)	C13—N7—C12—C11	174.08 (13)
N3—C3—C4—N1	179.23 (17)	N6—C11—C12—N7	−164.13 (13)
C2—N2—C5—C4	−1.0 (2)	C12—N7—C13—C14	−177.90 (14)
C2—N2—C5—O1	−179.64 (15)	C16—N7—C13—C14	59.27 (19)
N1—C4—C5—N2	−178.29 (16)	C15—O3—C14—C13	54.1 (2)
C3—C4—C5—N2	1.0 (3)	N7—C13—C14—O3	−57.0 (2)
N1—C4—C5—O1	0.53 (18)	C14—O3—C15—C16	−54.7 (2)
C3—C4—C5—O1	179.79 (14)	C12—N7—C16—C15	179.45 (16)
C1—O1—C5—N2	178.63 (15)	C13—N7—C16—C15	−59.5 (2)
C1—O1—C5—C4	−0.28 (17)	O3—C15—C16—N7	58.0 (2)
N1—C1—C6—C8	0.4 (3)		

Table S26. Geometry of proposed hydrogen bonds in **5h**.

D—H···*A*	*D*—H (Å)	H···*A* (Å)	*D*···*A* (Å)	*D*—H···*A* (°)
N5—H5*A*···O3^{i}	0.88	2.02	2.889 (2)	167
N5—H5*B*···N1	0.88	2.40	2.955 (3)	122
N6—H6···N4^{ii}	0.88	2.10	2.959 (3)	166
C9—H9*A*···N3^{iii}	0.98	2.68	3.650 (3)	172
C11—H11*A*···N1	0.99	2.54	3.050 (3)	112
C13—H13*B*···N2^{iv}	0.99	2.63	3.615 (3)	172

Symmetry codes: (i) −*x*, −*y*+1, −*z*; (ii) *x*+1, *y*−1, *z*; (iii) *x*−1, *y*+1, *z*; (iv) −*x*+1, −*y*+1, −*z*+1.

BIOLOGY

Table S27. Primer sequences used in his study.

Gen	5' - 3' sequence	
	mouse	human
ERK1	TACCTGGACCAGCTCAACCACATT	GTGGCCCCAGTTCAATCTC
	AGCAGGTCAAGAGCTTTGGAGTCA	GGGTTTGAATGAGATGAGGGG
ERK2	GGAACAGGTTGTTCCCAAATGCTGAC	TATTACGACCCGAGTGACGA
	CTCCTTAGGTAAGTCGTCCAACTCC	AAGAACACCGATGTCTGAGC
p38α	GAAGTCATCAGCTTTGTGCCACC	TATGCGTCTGACAGGAACAC
	CCACAAAGATAGGTGGACAGACG	GGGCCGCTGTAATTCTCTTA
p38β	TGGCACCCATGAAATTGAGCAGTG	ACAGTGGATATCTGGTCCGT
	GGCATATGTACACATCCGTGCATTCC	ATATATGTCCGGGCGTGTTC
p38γ	GGACATTTGGTCCGTTGGCTGCAT	GGCACATGGCCTGTGTAATA
	CCATGTAGTTCTTGGCCTCTGC	TAGGAAATGTCCCCCACCTT
p38δ	AGAAGGTTGGCCATCAAGAAGCTGA	CTGAGGTATATCCACGCTGC
	TCCAGAAGCCCAATGACGTTCTCA	TGTAGCGCATCCAATTCAAGA
JNK	AGCTCGGAACACCTTGTCCTGAAT	GCCTTCTCCTTCAGCACAG
	GGAGAGCTTCATCTACGGAGATCC	AGGCAGGCGGCTAGTCAC
Casp3	TGTCATCTCGCTCTGGTACG	TTCAGAGGGGATCGTTGTAGAAGTC
	AAATGACCCCTTCATCACCA	CAAGCTTGTCGGCATACTGTTTCAG
Casp8	ACAATGCCCAGATTTCTCCCTAC	GCAAAAGCACGGGAGAAAG
	CAGACAGTATCCCCGAGGTTTG	GGATACAGCAGATGAAGCAG
Casp9	AGCCAGATGCTGTCCCATAC	GAGTCAGGCTCTTCCTTTG
	CAGGAGACAAAACCTGGGAA	CCTCAAACTCTCAAGAGCAC
BCL2	CTCGTCGCTACCGTCGTGACTTCG	TATAAGCTGTCGCAGAGGGGCTA
	CAGATGCCGGTTCAGGTACTCAGTC	GTACTCAGTCATCCACAGGGCGAT
NF-κB	GAAATTCCTGATCCAGACAAAAAC	GAGTTACCTACCAGGGCTATTC
	ATCACTTCAATGGCCTCTGTGTAG	CTCTCCTCATCCTCACTCTCT
FAS	GCGATGAAGAGCATGGTTTAG	GACAAAGCCCATTTTTCTTCC
	GGCTCAAGGGTTCCATGTT	ATTTATTGCCACTGTTTCAGG
GADPH	CTCCACTCACGGCAAATTCAACGG	AGTCAGCCGCATCTTCTTTT
	GGTGAAGACACCAGTAGACTCCA	TGAGGTCAATGAAGGGGTCA
